# *In silico* analysis of the effect of HCV genotype-specific polymorphisms in Core, NS3, NS5A, and NS5B proteins on T-cell epitope processing and presentation

**DOI:** 10.3389/fmicb.2024.1498069

**Published:** 2025-01-15

**Authors:** Samina Baig, Assel Berikkara, Ramsha Khalid, Syed A. Subhan, Tanveer Abbas, Syed Hani Abidi

**Affiliations:** ^1^Department of Microbiology, University of Karachi, Karachi, Pakistan; ^2^Dow Institute of Medical Technology, Dow University of Health Sciences, Karachi, Pakistan; ^3^Department of Biomedical Sciences, Nazarbayev University School of Medicine, Astana, Kazakhstan; ^4^Department of Biochemistry, University of Karachi, Karachi, Pakistan

**Keywords:** HCV, CTL, polymorphism, genotype, subtype, adaptive immune system

## Abstract

**Background:**

HCV genotypes are 30–35% polymorphic at the nucleotide level, while subtypes within the same genotype differ by nearly 20%. Although previous studies have shown the immune escape potential of several mutations within the HCV proteins, little is known about the effect of genotype/subtype-specific gene polymorphism on T-cell immunity. Therefore, this study employed several *in silico* methods to examine the impact of genotype/subtype-specific polymorphisms in Core, NS3, NS5A, and NS5B sequences on T cell epitope processing and HLA-epitope interactions.

**Methods:**

For this study, 8,942, 17,700, 14,645, and 3,277 HCV Core, NS3, NS5A, and NS5B sequences, respectively, from eight genotypes and 21 subtypes were retrieved from the Los Alamos HCV Database. Next, the NetCTL tool was employed to predict Cytotoxic T Lymphocyte (CTL) epitopes based on combined proteasomal cleavage, TAP efficacy, and HLA class I receptor binding scores. PEP-FOLD was used to model selected epitopes, followed by peptide-HLA docking using HPEPDOCK. Finally, molecular dynamics simulations were conducted for 200 ns using Desmond software to analyze differences in HLA-epitope (from different HCV genotypes) interaction kinetics and dynamics.

**Results:**

A total of 3,410, 8,054, 6,532, and 14,015 CTL epitopes were observed in the HCV Core, NS3, NS5A, and NS5B sequences, respectively. Significant genotype/subtype-specific variations in CTL values and docking scores were observed among NS3, NS5A, and NS5B proteins. *In silico* results reveal that epitopes from genotype 6b (NS3), 6d/r (NS5B), 6o and 6 k (NS5A) exhibit higher immunogenicity than other genotypes, forming more energetically stable complexes with host receptors. These epitopes, compared to those from the same positions but different genotypes, showed binding energies of −144.24 kcal/mol, −85.30 kcal/mol, and − 43 kcal/mol, respectively. Over a 200 ns MD simulation, GT 6b and 6d/r epitopes displayed up to a 40% stronger binding energy with the HLA receptor. These findings suggest that patients infected with GT 6 may experience enhanced T cell responsiveness and broader immunogenicity.

**Conclusion:**

Our study suggests that genotype/subtype-specific polymorphism in HCV may result in altered immune responses by modulating T-cell epitope processing and interaction with HLA receptors. Further experimental studies can be performed to confirm the effect of genotype/subtype-specific polymorphisms on T cell-mediated immune response.

## Introduction

Hepatitis C Virus (HCV) is one of the major public health challenges worldwide (Gokhale et al., 2014). According to the 2023 WHO report, 58 million people globally live with chronic HCV infection (World Health Organization, 2024). HCV can establish acute and chronic infections (Sevvana et al., 2021). Acute infections tend to be asymptomatic, and the host system can clear the virus within six months of infection through the coordinated action of the innate and adaptive immune response (Grebely et al., 2012). However, the majority (70%) of HCV-infected individuals develop chronic infections, which can manifest as liver cirrhosis, fibrosis, and hepatocellular carcinoma (Chigbu et al., 2019). One reason for the progression from acute to chronic infection is the viral escape, attributed to immune-escape mutations, from CD8+ T cell response, especially in the acute infection stage (Cox et al., 2005a; Cox et al., 2005b; Neumann-Haefelin et al., 2008b). HCV epitope mutations occur in a targeted manner, with approximately half of class I epitopes acquiring immune escape mutations during acute infection, particularly in non-structural proteins (NS3, NS5B, NS2, and E1), though the pattern and stability of these mutations depend on both viral fitness requirements and host immune responses (Timm and Walker, 2015; Walker et al., 2024; Zhang et al., 2023a). These epitope variants facilitate viral persistence by reducing their binding affinity to Major Histocompatibility Complex (MHC), also known as Human Leukocyte Antigen (HLA) in humans, which subsequently can affect presentation to and recognition by the CD8+ cytotoxic T lymphocytes (CTLs) (Mei et al., 2022).

CTLs are major effector cells that produce cytokines, facilitate B cell maturation, and kill HCV-infected cells (Hofmann et al., 2021). However, under immune response pressure, HCV epitopes undergo mutations to evade recognition by CTLs, and these escape variants can even replace the wild-type sequence, adding new consensus sequences to the existing pool of variable viral antigens (Neumann-Haefelin et al., 2008a). *De novo* T cell response against mutated epitopes can be incomplete and might not even occur in chronic HCV infection due to the high viral load during persistent infection (Kemming et al., 2020).

Numerous studies have shown that HCV can amplify mutations under the selection pressure from the host immune system (Walker et al., 2015; Ulsenheimer et al., 2013), leading to increased genomic diversity observed at the level of genotypes, subtypes, and quasispecies (Walker et al., 2015). To date, about 8 genotypes and 86 subtypes of HCV have been identified (Hedskog et al., 2019). Genotypes (GT) are approximately 30–35% polymorphic at the nucleotide level, and subtypes can differ by approximately 20% within the same genotype (Galal et al., 2014; Simmonds et al., 1994).

The variations/mutations in the HCV genome can affect the virus epitope recognition by the host immune system, leading to failed viral clearance (Wölfl et al., 2008; Zhang et al., 2017; Merani et al., 2011). For example, Walker et al. (2015) showed differences in CD8+ T-cell responses due to a single amino acid substitution in a conserved epitope observed in HCV GT 3a compared to a wild-type epitope observed in 1a. Similarly, a cohort study of people reinfected with HCV demonstrated that host immune responses to a specific HCV genotype did not guarantee protection against heterogeneous HCV genotypes. These responses were associated with a 49% reduction in viral rebound compared to the initial viral clearance (Islam et al., 2017).

To our knowledge, most existing studies on HCV vaccine design focus on targeting specific HCV proteins (Walker et al., 2022; Dazert et al., 2009) or a set of epitopes without considering genotype specificity (Ikram et al., 2018). Our hypothesis is that *in silico* methods can demonstrate how epitopes from different genotypes/subtypes at the same sequence position may exhibit varying binding energies, potentially reflecting differences in T cell responsiveness.

Therefore, this study employed sequence analysis, immunoinformatics, molecular docking, and molecular dynamics simulation approaches to examine the effect of genotype/subtype-specific polymorphisms within the Core, NS3, NS5A, and NS5B sequences on HLA binding and CTL epitopes.

## Methodology

### Retrieval of HCV Core, NS3, NS5A, and NS5B sequences

A total of 8,942 sequences for HCV *core*, 17,700 sequences for nonstructural 3 (*NS3*) *protein*, 14,645 sequences for *NS5A*, and 3,277 sequences for *NS5B*, belonging to different genotypes (Gokhale et al., 2014; World Health Organization, 2024; Sevvana et al., 2021; Grebely et al., 2012; Chigbu et al., 2019; Cox et al., 2005a; Cox et al., 2005b) and subtypes (a, b, c, d, e, f, g, h, i, j, k, l, m, n, o, p, q, r, s, t, and u), were retrieved in FASTA format from the Los Alamos HCV Database^1^ (Kuiken et al., 2005).

### Sequence alignment, consensus sequence generation, translation, and identification of conserved and variable sites

The sequences corresponding to each genotype were aligned using MEGA 7.0 software with the ClustalW algorithm (Kumar et al., 2016). The sequence alignment for each gene was used to generate genotype-specific consensus sequences for each gene using the ‘Consensus maker’ tool^2^ (Kuiken et al., 2005). These consensus sequences were then translated into amino acid sequences using the ExPASy translate tool (Gasteiger et al., 2003). Subsequently, the genotype-specific consensus sequences of HCV Core, NS3, NS5A, and NS5B proteins were aligned using the ESPript 3.0 online web tool to identify conserved and variable regions, including genotype-specific amino acid variations (Robert and Gouet, 2014).

### Prediction of cytotoxic T lymphocyte (CTL) epitopes

The CD8+ T cell epitopes within genotype-specific Core, NS3, NS5A, and NS5B HCV sequences were predicted using the NetCTL 1.2 tool (Larsen et al., 2007), using default thresholds for proteasome (0.15), TAP (0.05), and epitope prediction (0.75). The default thresholds were based on the study by Larsen et al., which yielded prediction quality comparable to *in vitro* data (Larsen et al., 2005). The predicted epitopes were evaluated based on their combined score across various human leukocyte antigen (HLA) class I receptor supertypes, namely A1, A2, A3, A24, A26, B7, B8, B27, B39, B44, B58, and B62, along with their peptide binding affinities (CTL scores).

### Peptide modeling

*De novo* peptide structure prediction tool PEP-FOLD v2.0 (Thévenet et al., 2012) was used to generate three-dimensional models for the 22 genotype-specific epitopes selected according to their combined CTL scores (Table 1). From the ten models predicted by the PEP-FOLD tool, ‘model 1’ was selected due to its superior performance, including the highest global distance test score (GDT-TS), qualitative model energy analysis (Qmean) score, and template modeling score (TM-score), along with the lowest sOPEP (Optimized Potential for Efficient structure Prediction) energy.

**Table 1:** Genotype-specific CTL epitopes within HCV Core, NS3, NS5A, and NS5B protein sequences.

Protein	HLA-I	Starting position	Genotypes (GT)	Peptide	C. Score	PDB ID	PDB ID resolution	HPEPDOCK docking score (kcal/mol)
Core	A26	156	1b/c/e/e2/g/h/l/2a/l2/5a	R**VL**EDG**V**NY	0.77	1DUY	2.15 Å	−209.986
			6r/r2	R**TI**EDG**I**NY	1.76	1DUY	2.15 Å	−201.178
	B8	35	3c	YVLPRRGP**L**	1.82	1 M05	1.9 Å	−246.553
			3 h	YVLPRRGP**T**	0.79	1 M05	1.9 Å	−243.722
NS3	A1	1,618	1c	L**T**G**A**TPLLY	3.18	1DUY	2.15 Å	−225.364
			4d/l	L**R**G**P**TPLLY	0.77	1DUY	2.15 Å	−246.886
	A3	1,262	2f	L**G**FGAYM**A**K	0.83	1TMC	2.30 Å	−236.678
			6b	L**S**FGAYM**S**K	1.59	1TMC	2.30 Å	−264.334
	A26	1,368	1a/l/6 t	**ST**TGE**I**PFY	2.31	1DUY	2.15 Å	−222.975
			6d/k	**PS**TGE**V**PFY	0.77	1DUY	2.15 Å	−241.448
	B7	1,376	1a/l	YGKAIP**LEV**	0.76	1XH3	1.48 Å	−242.123
			3a	YGKAIP**IAL**	1.72	1XH3	1.48 Å	−251.624
	B39	1,032	1b	Y**S**QQTRGLL	0.76	4O2E	1.98 Å	−246.478
			6f/p/t	Y**H**QQTRGLL	2.05	4O2E	1.98 Å	−242.139
NS5A	A1	2,380	6 k	D**AG**SDAGSY	0.82	1DUY	2.15 Å	−183.443
			6o	D**TA**SDAGSY	2.51	1DUY	2.15 Å	−219.685
	B44	2,357	3a	**E**E**NS**S**S**SG**V**	0.96	1SYV	1.7 Å	−157.091
			3 h	**S**E**AP**S**T**SG**L**	1.75	1SYV	1.7 Å	−157.09
NS5B	A26	2,603	1e	**PI**A**V**MG**SS**Y	0.79	1DUY	2.15 Å	−242.464
			3b3/i	**ST**A**T**MG**AA**Y	2.20	1DUY	2.15 Å	−226.717
	B8	2,725	6d/r	AAKL**K**D**FDM**	0.76	1 M05	1.9 Å	−207.79
			5a	AAKL**R**D**CTL**	1.70	1 M05	1.9 Å	−176.316

The table shows data for different HCV proteins (column 1), restricting HLA-I molecules (column 2), the starting position of the epitope relative to the HCV genome (column 3), HCV genotypes harboring the epitope (column 4), the peptide sequence of the epitope with amino acid variations highlighted in red (column 5), CTL score (column 6), Protein Data Bank ID number of the HLA receptor and its resolution score in angstrom (Å) (columns 7 and 8), and binding energy score (in kcal/mol) for each receptor-peptide interaction predicted by the HPEPDOCK tool (column 9). The light-yellow shaded rows show epitopes restricted by the same HLA-I receptor but exhibit a difference in HPEPDOCK docking scores exceeding by 27.66 points (cut-off based on 1-standard deviation method).

### Peptide-HLA docking

To study peptide-HLA interactions, predicted HLA binders were first classified into supertypes based on the similarity of their peptide binding motifs and relevant immunological characteristics (Shen et al., 2023). Subsequently, the three-dimensional structures of HLA supertype A1 and A26 (PDB ID: 1DUY), supertype A3 (PDB ID: 1TMC), supertype B7 (PDB ID: 1XH3), supertype B8 (PDB ID: 1 M05), supertype B39 (PDB ID: 4O2E), and supertype B44 (PDB ID: 1SYV) were retrieved from the Protein Data Bank in the PDB format. The protein structures were processed to remove heteroatoms and water molecules using Discovery Studio Visualizer v4.5 (Dassault Systèmes, 2019).

Subsequently, each selected peptide was individually docked to the HLA-I molecule using the HPEPDOCK v2.0 web server (Zhou et al., 2018). This server employs a hierarchical algorithm for blind peptide-protein docking. For the docking, the HLA-I and peptide molecules were, respectively, used as the receptor and ligand. The binding values (docking scores) were determined using the same software, and the top ten poses for each peptide were retrieved and further analyzed using Discovery Studio Visualizer software.

### MD simulation

To select peptides for MD simulations, in the first step, the docking scores were analyzed using the one-standard deviation method to determine the cut-off (27.66 kcal/mol) (Luciani et al., 2005). The cut-off score was used to identify epitopes impacted by the genotype-specific mutations based on docking scores (based on the one-standard deviation method) of 27.66 points. HCV epitopes, which displayed a difference in HPEPDOCK docking scores exceeding 27.66 kcal/mol when bound to their respective HLA-I receptors, underwent a 200 ns molecular dynamics (MD) simulation using Desmond (Schrödinger LLC) software (Bowers et al., 2006). Prior to the simulations, the HLA-epitope complexes were optimized and minimized by applying Protein Preparation Wizard in Maestro. All systems were set up via the System Builder tool, utilizing the TIP3P solvent model in an orthorhombic box. The OPLS 2005 force field was chosen in the simulations, and counter ions were introduced to neutralize the models (Shivakumar et al., 2010). To replicate physiological conditions, sodium chloride (NaCl) was added to achieve a final concentration of 0.15 M, providing Na + and Cl − ions. The NPT ensemble with a temperature of 300 K and pressure of 1 atm was chosen for the entire simulation period of 200 nsec. The models were equilibrated before starting the simulation. Trajectories were saved for analysis every 200 ps, and the stability of the simulation was monitored by comparing the root mean square deviation (RMSD) of the protein over time (Maiorov and Crippen, 1994).

## Results

### Genotype-specific variations in Core, NS3, and NS5 proteins

The sequence analysis revealed a total of 66, 295, 329, and 322 genotype (GT)-specific amino acid variations in the Core, NS3 (peptidase and helicase), NS5A, and NS5B sequences, respectively (Supplementary Table S1). Additionally, we observed certain deletions in sequences from different genotypes. Specifically, deletions were found in NS3 at alignment positions 1–7 in GT in 6e only; in NS5A at alignment positions 261 to 264 in GTs 2a, 2b, 2c, 2i, 2j, 2 L, 2 l2, 2 m, and 2q, at positions 265–266 in GT 4a, at positions 263–266 in genotypes 4c, 4d, and 4f, at positions 263–366 in genotypes 4 L, 4 m, 4 m2, 4n, 4o, 4p, 4q, 4r, and 4v, and at position 293 in genotypes 4c, 4n, 4o, and 4p (Supplementary Table S1).

### Prediction of CTL epitopes and their restricting HLA receptors

To predict CD8+ T lymphocyte (CTL) epitopes within the Core, NS3, NS5A, and NS5B proteins, we used the NetCTL 1.2 tool, which employs combined proteasomal cleavage, transporter associated with antigen processing (TAP) efficacy, and HLA class I receptor binding scores to identify putative epitopes in a given sequence (Robert and Gouet, 2014). A total of 3,410, 8,054, 6,532, and 14,015 CTL epitopes were observed in the Core, NS3, NS5A, and NS5B sequences, respectively (Supplementary Tables S2–S5). Among these, 15, 39, 3, and 36 conserved epitopes were found within the Core, NS3, NS5A, and NS5B across all genotypes. Analysis of the conserved epitopes by region showed that most conserved epitopes were distributed in European and Asian countries such as France, Vietnam, China, Thailand, Portugal, Myanmar, Pakistan, etc. (Supplementary Figure S1). Additionally, 15, 146, 105, and 177 genotype-specific epitopes were identified in Core, NS3, NS5A, and NSB, respectively (Supplementary Tables S2–S5).

Genotypic variability within specific epitopes affected their binding affinity to the HLA class I receptor, particularly in regions where variations were observed (Table 1). For instance, an epitope at position 35 in the core domain in GT 3c had a sequence of YVLPRRGPL and exhibited a CTL score of 1.82 in complex with the HLA-B8 receptor. In contrast, in GT 3 h, the same epitope had a different sequence of YVLPRRGPT with a mutation at Y43T, resulting in a CTL score of 0.79 (Table 1). Similarly, the epitope LTGATPLLY, restricted by HLA-A1 in GT 1c at position 1,618 in the NS3 protein, had a binding score of 3.18. Conversely, in GTs 4d and 4 L, the same epitope had an altered sequence of LRGPTPLLY, with mutations at T1619R and A1621P, resulting in a score of 0.77 (Table 1). Another epitope, DTASDAGSY, restricted by HLA-A1 in GT 6o at position 2,380 in the NS5A protein, had a CTL score of 2.51. On the contrary, in genotype 6 k, the same epitope had a variant sequence of DAGSDAGSY with two amino acids changed at T2381A and A2382G, resulting in a differing CTL score of 0.82 (Table 1).

### Peptide modeling and peptide-HLA molecular docking

**Figure 1:**
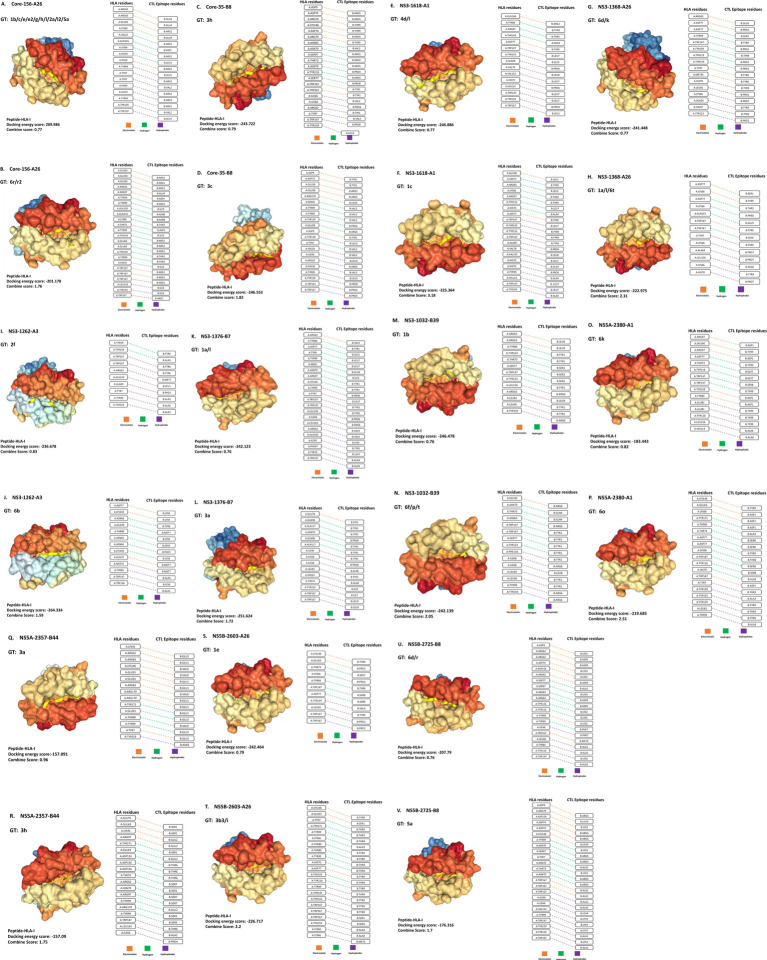
Interactions of HLA-I receptors with HCV CTL epitopes. The figure illustrates 22 HLA-epitope complexes **(A–V)** (left panel) and the amino acid interactions between HLA-I and CTL epitopes (right panel). Each pair is labeled with the HCV protein name, its position, and the HLA-I receptor name (e.g., Core-156-A26). The types of interactions are represented by orange, green, and violet colors, corresponding to electrostatic, hydrogen, and hydrophobic interactions, respectively.

In the next step, a molecular docking assay was performed to investigate the influence of genotype-specific variations on peptide-HLA binding. 11 pairs of CTL epitopes, each pair consisting of peptides with lower and higher CTL docking scores, were docked with an HLA class I receptor, using the HPEPDOCK tool (Table 1, Figures 1U–V). Among these, three pairs exhibited a difference in HPEPDOCK docking scores exceeding 27.66 (cut-off based on the 1-standard deviation method) points (Table 1, light-yellow shaded rows). These include peptides derived from HCV NS3, NS5A, and NS5B proteins, starting at positions 1,262, 2,380, and 2,725, respectively.

The first pair consisted of peptides from the NS3 protein restricted by HLA-A3 (Figures 1I,J). Peptide-1 ‘LGFGAYMAK’ was observed in GT 2 f. In contrast, peptide-2 ‘LSFGAYMSK’ observed in GT 6b exhibited two distinct amino acid variations: Peptide-1 had ‘G’ (Glycine) at the second position and ‘A’ (Alanine) at the eighth position. At the same time, Peptide-2 had ‘S’ (Serine) at the second and eighth positions, respectively. Peptide-2 showed a higher affinity (−264.33 kcal/mol) than the variant peptide-1 (−236.67 kcal/mol), indicating a stronger binding affinity toward HLA-A3.

The second pair comprised peptides from the NS5A domain, restricted by HLA-A1 (Figures 1O,P). Peptide-1 ‘DAGSDAGSY’ observed in GT 6 k and peptide-2 ‘DTASDAGSY’ observed in GT 6o exhibited two amino acid variations: Peptide-1 had ‘A’ (Alanine) and ‘G’ (Glycine) at the second and third positions, respectively. In contrast, Peptide-2 had ‘T’ (Threonine) and ‘A’ (Alanine) at the second and third positions, respectively. Peptide-1 exhibited a docking lower affinity (−183.44 kcal/mol) than peptide-2 (−219.68 kcal/mol), suggesting its weaker binding affinity for HLA-A1.

The third pair consisted of peptides from the NS5B domain, restricted by HLA-B8 (Figures 1U,V). Peptide-1 ‘AAKLRDCTL’ observed in GT 5a and peptide-2 ‘AAKLKDFDM’ observed in GTs 6d and 6r exhibited four amino acid variations: Peptide-1 had ‘K’ (Lysine), ‘F’ (Phenylalanine), ‘D’ (Aspartic acid), and ‘M’ (Methionine) at the fifth, seventh, eighth, and ninth positions, respectively, while Peptide-2 had ‘R’ (Arginine), ‘C’ (Cysteine), ‘T’ (Threonine), and ‘L’ (Leucine) at the same positions. Peptide-1 exhibited a lower affinity (−176.31 kcal/mol) than peptide-2 (−207.79 kcal/mol), indicative of a weaker binding affinity than peptide-2 when complexed with the HLA-B8 receptor.

### Molecular dynamics simulation

The molecular dynamics simulation analysis was performed on three pairs of HCV epitopes that showed a difference in HPEPDOCK docking scores exceeding 27.66 using the one standard deviation estimation (Table 1). Throughout the MD simulation, binding free energy (ΔG(bind)) and hydrogen-bond (HB) enthalpies (ΔH-bond) were measured for the six HLA-epitope complexes (Table 2). Negative values were attributed to ΔG(bind) in each complex.

**Table 2:** Energy components’ contributions to the MD simulation of receptor-epitope models.

Protein	Epitope starting position	Epitope	Genotype	Receptor	ΔG(bind) (kcal/mol)	ΔG HB (kcal/mol)
NS3	1,262	LGFGAYMAK	2f	HLA-A3	−85.30	−3.76
		LSFGAYMSK	6b		−144.24	−8.11
NS5A	2,380	DAGSDAGSY	6 k	HLA-A1	−43.03	−3.51
		DTASDAGSY	6o		−42.39	−5.65
NS5B	2,725	AAKLRDCTL	5a	HLA-B8	−51.38	−7.40
		AAKLKDFDM	6d/r		−87.78	−4.25

ΔG(bind), binding free energy; ΔH(bind), hydrogen-bond correction energy.

#### Subtype-specific variations in NS3 protein-derived peptides LGFGAYMAK (GT 2f) and LSFGAYMSK (GT 6b)

**Figure 2:**
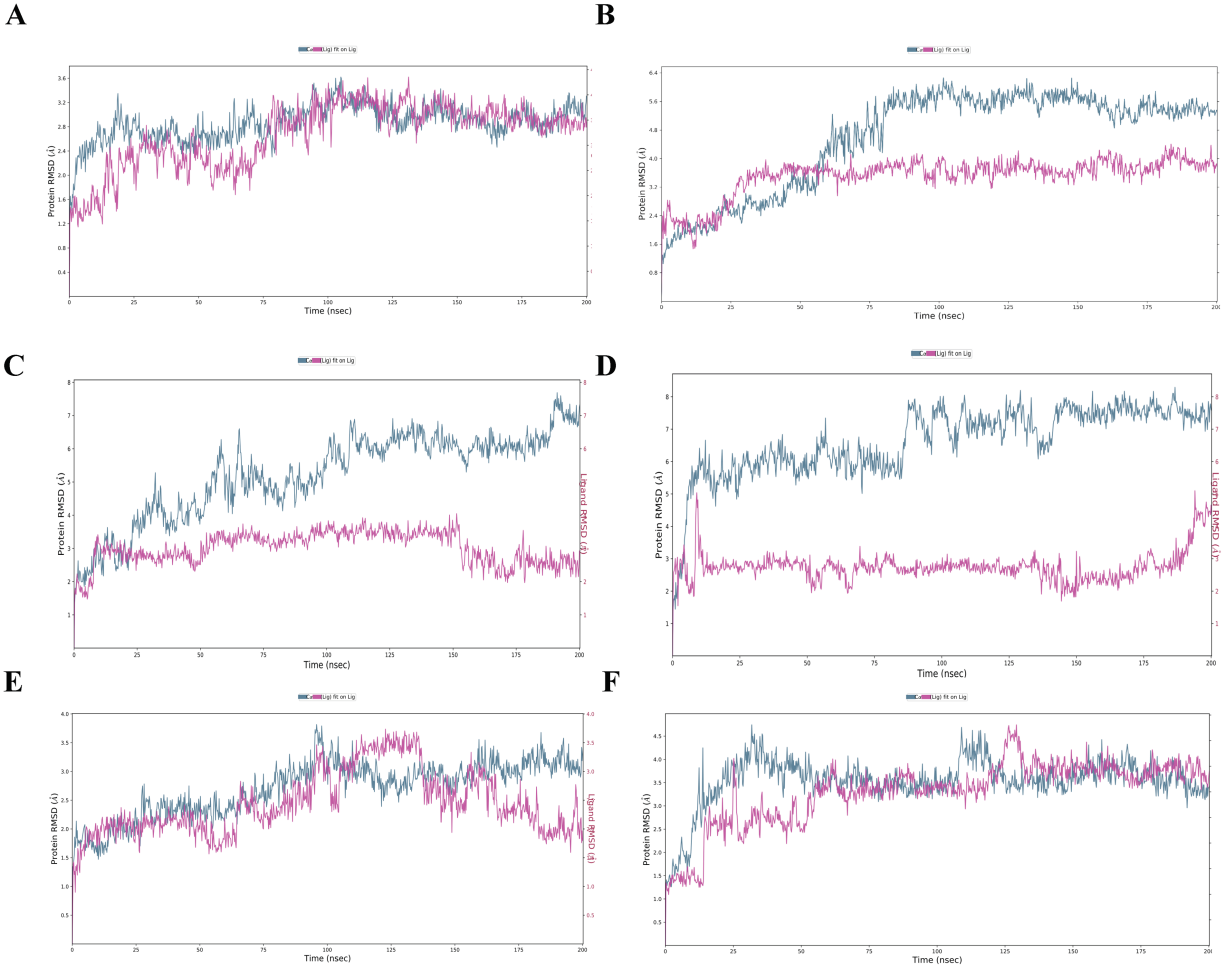
RMSD of the docking models. RMSD for the complexes. **(A)** A3-LGFGAYMAK, **(B)** A3-LSFGAYMSK, **(C)** A1-DAGSDAGSY, **(D)** A1-DTASDAGSY, **(E)** B8-AAKLKDFDM, and **(F)** B8-AAKLRDCTL. The blue line on the left Y-axis illustrates the RMSD of the protein atoms, while the red line represents the ligand RMSD, indicating the ligand’s stability to the protein and its binding pocket. The X-axis shows the timeline of the simulation in picoseconds over 200,000 ps (equivalent to 200 nanoseconds).

HCV NS3 protein-derived peptides LGFGAYMAK (GT 2f) and LSFGAYMSK (GT 6b) at position 1,260 formed complexed with the HLA-A3 receptor. Following a 200 ns molecular dynamics simulation, the peptide from GT 6b exhibited higher binding energy, with a ΔG(bind) value of −144.24 kcal/mol, compared to the peptide from GT 2f, which had a binding free energy of −85.30 kcal/mol (Table 2). While ΔH-bond value was more negative for GT6b epitope, the root mean square deviation (RMSD) graph for the peptide from GT 2f showed instability, with an increasing RMSD value for the peptide. In contrast, the RMSD values for the HLA receptor complexed with the peptide from GT 6b remained stable, with the peptide RMSD reaching a plateau at 3.3 Å early in the simulation (Figures 2A,B).

#### Subtype-specific variations in NS5A protein-derived peptides DAGSDAGSY (GT 6k) and DTASDAGSY (GT 6o)

HCV NS5A protein-derived peptides DAGSDAGSY (GT 6 k) and DTASDAGSY (GT 6o), both starting at position 2,380, were paired with the HLA-A1 receptor. Over the course of the MD simulation, both peptides from genotypes 6 k and 6o exhibited strong binding energies of −43.03 kcal/mol and − 42.39 kcal/mol, respectively. The ΔH-bond values were also comparable, with −3.51 kcal/mol for DAGSDAGSY (GT 6 k) and − 5.65 kcal/mol for DTASDAGSY (GT 6o) (Table 2). In the complexes HLA-A1-DAGSDAGSY and A1-DTASDAGSY, both peptides demonstrated stable and equilibrium binding, as indicated by RMSD values of 6 Å (GT 6 k) and 7 Å (GT 6o), with the ligands remaining superimposed within the receptor’s binding site (Figures 2C,D).

#### Subtype-specific variations in NS5B protein-derived peptides AAKLKDFDM (GT 6d/r) and AAKLRDCTL (GT 5a)

NS5B protein-derived peptides AAKLKDFDM (GT 6d/r) and AAKLRDCTL (GT 5a), both originating from position 2,725, were bound to the HLA-B8 receptor for a 200 ns MD simulation. The peptide from GT 6d/r exhibited stronger binding free energy, with a ΔG(bind) value of −87.78 kcal/mol, compared to the peptide from GT 5a, which had a ΔG(bind) of −51.38 kcal/mol. However, the ΔH-bond energy for the peptide from GT 5a (−7.40 kcal/mol) was twice as low as that for GT 6d/r (−4.25 kcal/mol) (Table 2). While the RMSD for the peptides oscillated around 3 Å for GT 6d/r and 2.5 Å for GT 5a, the RMSD graph for the receptor showed instability throughout the MD simulation (Figures 2E,F).

## Discussion

The adaptive immune system, primarily CD8+ T lymphocytes (CTL), is crucial in limiting the HCV viral load from 4 to 8 weeks after infection (Neumann-Haefelin et al., 2007). However, in 70% of individuals, the immune response fails to eliminate the virus, eventually leading to chronic viremia (Chigbu et al., 2019; Neumann-Haefelin et al., 2007). A previous study suggests genetic variability is one reason for this failure (Lapa et al., 2019). Genetic variation in the HCV genome is primarily driven by low-fidelity RNA polymerase (Neumann-Haefelin et al., 2007; Lapa et al., 2019), resulting in a mutation rate of 3.5 × 10^−5^ to 1.2 × 10^−4^ substitutions per round of replication (Gokhale et al., 2014; World Health Organization, 2024; Sevvana et al., 2021). HCV genetic diversity is evident at the genotype (GT) level (GTs 1–7), with a 25–35% difference in nucleotide sequence between genotypes, and at the subtype level (defined by letters such as 1a, 2b, 3c, etc.), with a 10–20% difference within each subtype (Hedskog et al., 2019; Galal et al., 2014; Simmonds et al., 1994; Nakamura et al., 2022). Additionally, these genotypes and subtypes differ in their epidemiology, drug response, and interaction with the host immune system (Wölfl et al., 2008; Zhang et al., 2017; Merani et al., 2011; Nakamura et al., 2022; Echeverría et al., 2015). Our study aimed to identify genotype/subtype-specific polymorphisms within the Core, NS3, NS5A, and NS5B sequences on HLA binding and CTL epitopes. We examined 15, 146, 105, and 177 genotype/subtype-specific epitopes in the Core, NS3, NS5A, and NS5B proteins, respectively.

Overall, we found 66, 295, 329, and 322 genotype-specific variations in the Core, NS3, NS5A, and NS5B protein sequences, respectively. Genotype-specific variations in HCV strains impact clinical outcomes and drug responsiveness. For instance, previous clinical studies have identified HCV genotype 3 (GT3) as a risk factor for rapid liver fibrosis and hepatocellular cancer (McMahon et al., 2017). Additionally, prior studies identified epistasis in the HCV sequence as a driver of drug resistance, suggesting that HCV genetic diversity can weaken host immune responses and contribute to resistance mechanisms (Zhang et al., 2023b). These findings underscore the importance of considering immunogenic variation among genotype-specific HCV strains in vaccine design and drug development. Polymorphisms, specifically in the NS3, NS5A, and NS5B non-structural proteins, exhibited the most significant genotype/subtype-specific alterations. The NS3 protein has helicase and serine protease activity. The latter can cleave and inactivate host proteins essential for the innate immune system (Raney et al., 2010). NS5A is crucial for viral RNA replication and modulation, and it can suppress both innate and adaptive immunity, leading to chronic infection (Raney et al., 2010; Kriegs et al., 2009). NS5B, part of the HCV replication complex, can harbor mutations associated with resistance to direct-acting antivirals (Nguyen and Van Le, 2022). Therefore, we hypothesize that genotype/subtype-specific polymorphisms, especially in the non-structural proteins, might be responsible for downregulating CTL-mediated immune responses.

Our analysis showed that 11 pairs of epitopes (predicted using NetCTL) were affected by genotype/subtype-specific polymorphisms at both sequence level and in CTL scores (Table 1). For example, mutations Y43T in GT 3 h, T1619R and A1621P in GT 4d/l, and T2381A and A2382G in GT 6 k resulted in altered CTL scores compared to their paired epitopes from other genotypes/subtypes. NetCTL tool has been employed with similar settings applied in studies of pathogens such as *M. tuberculosis* (Bibi et al., 2021), SARS-CoV-2, and the Omicron variant (Khairkhah et al., 2020; Aasim et al., 2022). Among various prediction tools, NetCTL has demonstrated superior predictive performance for HIV epitopes compared to alternative tools such MAPP, EpiJen, WAPP, and MHC-pathway (Larsen et al., 2005), which significantly increases the confidence in the findings.

In the next step, we employed immunoinformatic techniques, namely HPEPDOCK and MD simulation, to examine interactions between subtype-specific epitopes and the restricting HLA molecules. *In silico* methods have widely been employed in vaccine design for their ability to screen numerous epitopes simultaneously and narrow down candidates for further *in vitro* and *in vivo* validation (Elshafei et al., 2024; Jyotisha and Qureshi, 2020). This approach enhances cost and time efficiency in the preclinical stages of vaccine development.

The HLA-peptide docking analysis identified three pairs of epitopes within NS3, NS5A, and NS5B proteins that exhibited a difference in HPEPDOCK scores exceeding one standard deviation (27.66 kcal/mol). Using standard deviation thresholds to define cutoffs is an accepted practice in structural bioinformatics and docking analyses, and has been used in previous studies (Kuhn et al., 2020; Cheng et al., 2010). Our results are supported by a previous study, showing that the CTL-mediated response in GT 3a, unlike in GT 1a, exclusively targeted non-structural HCV proteins during chronic infection (Keikha et al., 2020). In our study, the epitope LGFGAYMAK (GT 2f) in the NS3 protein exhibited a CTL score of 0.83 and a docking score of −236.67 kcal/mol, whereas the epitope LSFGAYMSK (GT 2b) had higher CTL and lower docking scores of 1.59 and − 264.33 kcal/mol, respectively. However, the opposite trend was observed for the NS5B protein, where the epitope AAKLKDFDM (GT 6d/r) resulted in a lower CTL score (0.76) and higher docking score (−207.79 kcal/mol) compared to the epitope AAKLRDCTL (GT 5a) with CTL score of 1.70, docking score of −176.31 kcal/mol. A similar phenomenon was reported by Wang et al. (2010), where variant NS3 epitopes showed a diminished CTL response compared to wild-type sequences. However, none of the variant sequences abolished HLA receptor binding, and the difference in HLA-docking complexes with either variant or wild-type epitopes was insignificant. Nonetheless, a previous study on HCV GT 1b sequences from a single-source outbreak in Irish women, using an HLA-epitope binding prediction model, reported a contrary trend (Kim et al., 2011). The study showed a 100-fold shorter dissociation time with the HLA-B*57 receptor compared to a reference sequence from GT 1a. Additionally, the interferon-*γ* staining assay showed no T cell response to variant epitopes, leading to chronic viremia. The authors claimed that HCV sequence variations, predominantly in the E2 and NS5 proteins, diminished virologic control by the HLA-B*57-mediated T-cell response (Kim et al., 2011).

In the next step, we used molecular dynamic (MD) simulation throughout 200 ns to explore the dynamics of the epitope-receptor complex over time. An NPT ensemble was employed to maintain constant temperature and pressure throughout the simulation. These conditions (300 K or 27°C and 1 atm or 760 mmHg) were selected to align with similar studies (Ren et al., 2022) and to reflect physiological conditions, as well as standard *in vitro* settings for HCV experiments (Song et al., 2010). The addition of NaCl stabilizes electrostatic interactions and ensures a neutral charge in the simulation box (Ribeiro et al., 2012). Also, a concentration of 0.15 M NaCl is a physiological salt level within mammalian cells, which more closely replicates *in vitro* conditions.

To highlight differences in binding energy among epitopes positioned at the same HCV sequence but originating from different genotypes, we analyzed binding energy (ΔG). Generally, more negative binding energy indicates a stronger interaction within the epitope-HLA complex, enhancing the likelihood of T-cell activation (Corse et al., 2011). There is a growing body of research linking negative ΔG values to strong protein interactions, confirmed through *in vitro* methods such as ELISA (Jain and Baranwal, 2019), X-ray crystallography (Li et al., 2023), and cryo-EM (Sušac et al., 2022), as well as in combination with other immunoinformatics tools (Morgan et al., 2024).

In our study, ΔG differences observed in MD simulations followed the trend seen in the docking results. For example, the epitope LSFGAYMSK in GT 6b resulted in a twice as low ΔG of −144.24 kcal/mol compared to the LGFGAYMAK epitope in GT 2f (−85.30 kcal/mol). Similarly, AAKLRDCTL from HCV GT 5a resulted in a weaker ΔG of −52.38 kcal/mol compared to AAKLKDFDM in HCV GT 6d/r (−87.78 kcal/mol). Finally, both DTASDAGSY (GT 6o) and DAGSDAGSY (GT 6 k) exhibited a similar binding energy value of −42.39 kcal/mol and 43.03 kcal/mol, respectively.

We analyzed binding energy in percentage terms to highlight differences in binding energy among epitopes located at the same HCV sequence position but from different genotypes. In MD simulation, epitopes from GT 6b, and 6d/r exhibited up to a 40% variation in binding energy when interacting with the HLA receptor. In previous studies, epitopes with binding energy (ΔG) to HLA receptors ranging from 18 to 43% lower were identified as having strong binding potential, which is critical for vaccine design (Mei et al., 2022; Kyobe et al., 2024; Chieochansin et al., 2024). In contrast, a 40% decrease in binding energy can weaken the interaction, potentially diminishing or even eliminating the T-cell response to the virus. These results also follow the same trend observed by Khairkhah et al. (2020), suggesting that immune-resistance mutations, capable of escaping both CTL responses and HLA recognition, can generate at the nucleotide level, leading to novel quasispecies and subtypes circulating within the population (Lapa et al., 2019).

*In vitro* study of HCV-derived T cell epitopes showed that sequence variations in HCV can modulate NK cell functions, creating potential pathways for viral immune escape (Lunemann et al., 2016). These findings underscore the importance of considering immunogenic variation among genotype-specific HCV strains in vaccine design and drug development. As we mentioned in the introduction, one primary reason for the progression from acute to chronic infection is the ability of HCV to evade the CD8+ T cell response, through immune-escape mutations, during the acute infection stage (Cox et al., 2005a; Cox et al., 2005b; Neumann-Haefelin et al., 2008b). Research by Bulteel et al. (2016) showed that spontaneous clearance of HCV is rare, occurring at a rate of 0.36 per 100 person-years, while 60–80% of infected individuals develop chronic infection (Saraceni and Birk, 2021). This indicates that even a robust immune response may not always prevent disease progression. Moreover, around 20% of patients with chronic HCV may develop liver cirrhosis within 25 years (Saraceni and Birk, 2021; Rustgi, 2007), and 30% of chronically diseased individuals are at risk of hepatocellular carcinoma (Khatun et al., 2021). Therefore, understanding how HCV’s high genetic variability affects immune recognition is crucial for predicting disease progression and determining treatment approaches (Saraceni and Birk, 2021).

Our results indicate that epitopes from genotype 6b (NS3), 6o and 6 k (NS5A), 6d/r(NS5B) exhibit higher immunogenicity compared to other genotypes, forming more energetically stable complexes with the host receptor (Table 1, light-yellow shaded rows). These findings suggest that patients infected with GT 6 may have better T cell responsiveness and broad immunogenicity. While 46.2% of all HCV cases in the world are attributed to GT1; GTs 2, 4, and 6 together make up 22.8% of cases (Messina et al., 2015). HCV genotype 6 emerged in the 2000s and has become the third most prevalent genotype in Southern Asia and surrounding regions (including China, Thailand, Indonesia, Cambodia, Malaysia, Myanmar, and Vietnam) (Irekeola et al., 2021). Here GT 6 represents 30–40% of HCV infections (Nguyen and Nguyen, 2015). According to the World Bank data, these regions are low, and low-middle-income countries (World Bank Country and Lending Groups, 2024), where access to medical treatments is limited, and many people rely on traditional remedies (Nguyen and Nguyen, 2015). While genotype 6 is less common in Western countries than GTs 1, 2, and 3, the limited preclinical and clinical data from these areas aids further investigation into GT 6 (Nguyen and Nguyen, 2015). HCV GT 6 also has distinct endemic subtypes, and its genetic diversity raises concerns about resistance to pan-genotypic direct-acting antiviral treatments. Additionally, although individuals infected with HCV GT 6 exhibit clinical symptoms similar to those of other HCV genotypes (Chao et al., 2011), numerous studies have revealed striking differences in drug therapy responses specific to GT6 (Nguyen and Nguyen, 2015; Flower et al., 2021). Notably, a previous study found that patients infected with HCV GT 6 responded better to interferon therapy compared to patients infected with genotype 1 (Hui et al., 2003). Also, GT 6 patients have been shown to experience fewer severe clinical outcomes, such as cirrhosis (Yao et al., 2020). These findings highlight the importance of considering immunogenic variation among genotype-specific HCV strains in vaccine design and drug development.

Overall, this study used sequence analysis, immunoinformatic, molecular docking, and molecular dynamics simulation to analyze a large HCV sequence dataset to identify genotype- and subtype-specific polymorphisms that can impact CD8+ T cell epitope processing and HLA-epitope interactions. *In silico* techniques have widely been employed in vaccine design, offering the advantage of screening numerous epitopes simultaneously and narrowing down potential candidates for subsequent *in vitro* and *in vivo* validation (Elshafei et al., 2024; Jyotisha and Qureshi, 2020). This approach contributes to preclinical steps in vaccine development by enhancing cost and time efficiency. Our results indicate that epitopes from genotype 6b (NS3), 6o and 6 k (NS5A), 6d/r(NS5B) exhibit higher immunogenicity compared to other genotypes, forming more energetically stable complexes with the host receptor. These findings suggest that patients infected with GT 6 may have better T cell responsiveness and broad immunogenicity.

The emergence of personalized medicine has facilitated more targeted therapies that account for individual, cultural, and geographical diversity (Wang and Wang, 2023). Due to the global variation in HCV genotypes and subtypes, there has been an increasing discussion about designing a genotype/subtype-targeted vaccine (Zhang et al., 2023b). This strategy could be especially beneficial since HCV subtypes differ in binding affinity to HLA receptors, leading to varying TCR responses. Prior studies on other viral infections have highlighted the effectiveness of genotype-specific vaccines (Kim et al., 2024; Mathew et al., 2023; Haslwanter et al., 2022). We propose that HCV subtype/genotype-specific vaccines may help prevent the emergence of new quasispecies, which can arise from accumulating immune escape mutations.

We identified several limitations in our study. Firstly, the analysis was conducted using *in silico* tools. Although we tested the viral epitopes with multiple approaches, including epitope prediction, HLA-peptide docking models, and MD simulations, each step has its own limitations. For example, although MD simulations are much superior to docking, they are constrained by timescales, as longer timescales (which may be required for biological processes) may be highly computationally demanding and cannot be handled by conventional systems (Wang et al., 2019). Additionally, MD simulations may have limited ability to sufficiently sample peptide-protein interactions and/or capture rare events such as peptide unbinding without an enhanced sampling technique (Wang et al., 2019; Aghajani et al., 2022). Therefore, further *in vitro* and *in vivo* validation is necessary to fully establish the impact of genotype/subtype-specific polymorphisms on the adaptive immune response. For instance, T cells can be co-cultured with antigen-presenting cells displaying the epitopes and T-cell activation can then be measured using cytokine release assays like ELISPOT (for IFN-*γ*) and flow cytometry for activation markers (CD69, CD25) to confirm immune response (De Groot et al., 2001). Secondly, this analysis focused solely on CTL epitopes, given the significant role of CTL responses in HCV control (Hofmann et al., 2021; Larrubia et al., 2015; Nelson et al., 1997). Future studies should expand the analysis by including CD4 and B-cell epitopes to examine the impact of genotype/subtype-specific polymorphisms on other immune response components. Thirdly, the last major update to the Los Alamos HCV database was in 2007, which may exclude newer HCV sequences. Additionally, certain low-income regions remain underrepresented, potentially leading to biases that underestimate genetic diversity in these areas. Despite these limitations, research using sequences from the Los Alamos HCV database has continued to yield valuable insights into HCV genetics over the past five years (García-Crespo et al., 2020; Da Silva et al., 2019; García-Crespo et al., 2021). Finally, given the constraints of comparing single docking scores, statistical methods such as Z-scores or percentile-based cutoffs were not applicable. Therefore, the one-standard deviation method was selected as a simple, quantifiable criterion that streamlined the selection process. Although we acknowledge that this method may be less robust than standard statistical tests, our primary focus is on highlighting the biologically relevant differences in genotype-specific immunogenicity of HCV.

In conclusion, our study demonstrates that differential CTL responses to HCV can emerge due to genotype/subtype-specific variants, thereby challenging HCV control and altering disease dynamics in patients infected with different subtypes. In addition to CD8+ T cell escape, amino acid substitutions in HCV proteins of specific subtypes affect HLA binding, suggesting a dual antagonizing effect on the host adaptive immune system. In this light, along with previously reported results on the immune evasion of mutated HCV epitopes (Neumann-Haefelin et al., 2008b; Spear et al., 2016; Khan et al., 2022; Osuch et al., 2022), the immune escape mechanism of the virus appears to be facilitated by viral genetic polymorphism at the level of genotypes and subtypes. This insight might be valuable for designing vaccines considering HCV genotype/subtype-specific differences to optimize host immune responses.

## Data Availability

The original contributions presented in the study are included in the article/Supplementary material, further inquiries can be directed to the corresponding author.

## Footnotes

^1^ https://hcv.lanl.gov/content/index

^2^ http://www.hiv.lanl.gov/content/sequence/CONSENSUS/consensus.html
