# Supplementary figures and images for: In silico analysis of the effect of HCV genotype-specific polymorphisms in Core, NS3, NS5A, and NS5B proteins on T-cell epitope processing and presentation
Source: Front Microbiol. 2025 Jan 15;15:1498069. doi: 10.3389/fmicb.2024.1498069 (PMC11774985; doi:10.3389/fmicb.2024.1498069)

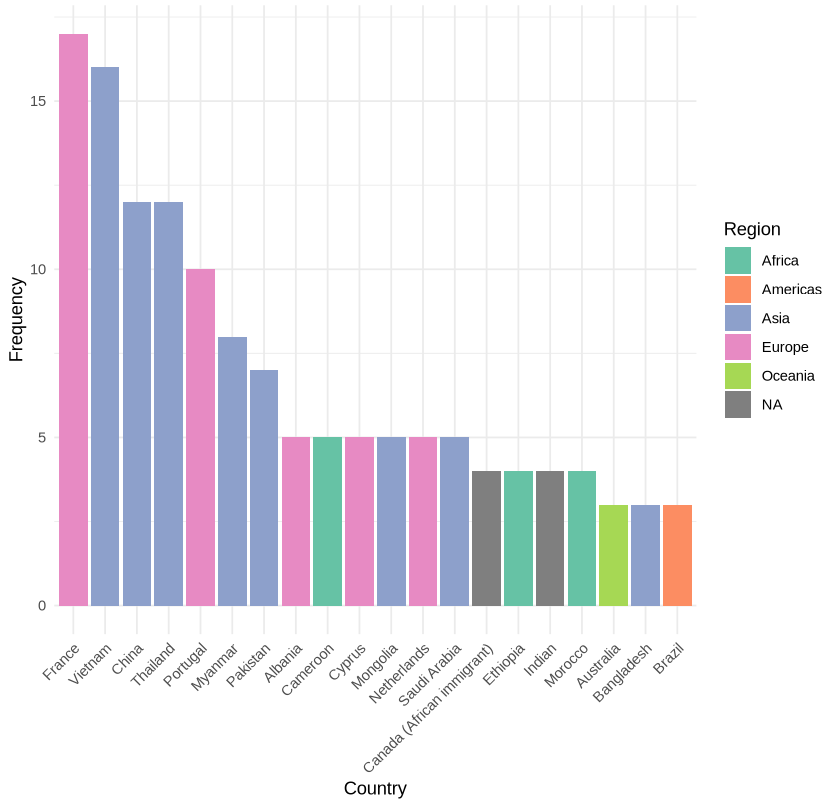

Supplement: Supplementary file 1 [file Image_1.png]
