# Supplementary material for: In silico analysis of the effect of HCV genotype-specific polymorphisms in Core, NS3, NS5A, and NS5B proteins on T-cell epitope processing and presentation
Source: Front Microbiol. 2025 Jan 15;15:1498069. doi: 10.3389/fmicb.2024.1498069 (PMC11774985; doi:10.3389/fmicb.2024.1498069)

Supplementary Table S1: Genotype-specific amino acid variation in HCV core, NS3, NS5A and NS5B proteins.
Fully conserved regions are shaded in red.

HCV Core

	1 · 10 · 20 · 30 · 40 · 50 · 60
**1aCON**	MSTNPKPQRKTKRNTNRRPQDVKFPGGGQIVGGVYLLPRRGPRLGVRATRKTSERSQPRGRRQP
**1bCON**	MSTNPKPQRKTKRNTNRRPQDVKFPGGGQIVGGVYLLPRRGPRLGVRATRKTSERSQPRGRRQP
**1cCON**	MSTNPKPQRKTKRNTNRRPQDVKFPGGGQIVGGVYLLPRRGPRVGVRATRKTSERSQPRGRRQP
**1eCON**	MSTNPKPQRKTKRNTNRRPQDVKFPGGGQIVGGVYVLPRRGPKLGVRAARKTSERSQPRGRRQP
**1e2CON**	MSTNPKPQRKTKRNTNRRPQDVKFPGGGQIVGGVYLLPRRGPRLGVRAARKTSERSQPRGRRQP
**1gCON**	MSTNPKPQRKTKRNTNRRPQDVKFPGGGQIVGGVYLLPRRGPRLGVRAARKTSERSQPRGRRQP
**1hCON**	MSTNPKPQRKTKRNTNRRPQDVKFPGGGQIVGGVYLLPRRGPRLGVRAARKTSERSQPRGRRQP
**1lCON**	MSTNPKPQRKTKRNTNRRPQDVKFPGGGQIVGGVYLLPRRGPRLGVRATRKTSERSQPRGRRQP
**2aCON**	MSTNPKPQRKTKRNTNRRPQDVKFPGGGQIVGGVYLLPRRGPRLGVRATRKTSERSQPRGRRQP
**2bCON**	MSTNPKPQRKTKRNTNRRPQDVKFPGGGQIVGGVYLLPRRGPRLGVRATRKTSERSQPRGRRQP
**2cCON**	MSTNPKPQRKTKRNTNRRPQDVKFPGGGQIVGGVYLLPRRGPRLGVRATRKTSERSQPRGRRQP
**2dCON**	MSTNPKPQRKTKRNTNRRPQDVKFPGGGQIVGGVYLLPRRGPRLGVRATRKTSERSQPRGRRQP
**2eCON**	MSTNPKPQRKTKRNTNRRPQDVKFPGGGQIVGGVYLLPRRGPRLGVRATRKTSERSQPRGRRQP
**2fCON**	MSTNPKPQRKTKRNTNRRPQDVKFPGGGQIVGGVYLLPRRGPRLGVRAARKTSERSQPRGRRQP
**2f2CON**	MSTNPKPQRITKRNTNRRPQDVKFPGGGQIVGGVYLLPRRGPRLGVRATRKTSERSQPRERRQP
**2iCON**	MSTNPKPQRKTKRNTNRRPQDVKFPGGGQIVGGVYLLPRRGPRLGVRATRKTSERSQPRGRRQP
**2jCON**	MSTNPKPQRKTKRNTNRRPQDVKFPGGGQIVGGVYLLPRRGPRLGVRATRKTSERSQPRGRRQP
**2kCON**	MSTNPKPQRKTKRNTNRRPQDVKFPGGGQIVGGVYLLPRRGPRLGVRATRKTSERSQPRGRRQP
**2lCON**	MSTNPKPQRQTKRNTNRRPQDIKFPGGGQIVGGVYLLPRRGPRVGVRATRKTSERSQPRGRRQP
**2l2CON**	MSTNPKPQRQTKRNTNRRPQDVKFPGGGQIVGGVYLLPRRGPRVGVRATRKTSERSQPRGRRQP
**2mCON**	MSTIPKPQRKTKRNTNRRPQDVKFPGGGQIVGGVYLLPRRGPRLGVRAARKTSERSQPRGRRQP
**2qCON**	MSTNPKPQRKTKRNTNRRPQDVKFPGGGQIVGGVYLLPRRGPRLGVRATRKTSERSQPRGRRQP
**2q2CON**	MSTNPKPQRKTKRNTNRRPQDVKFPGGGQIVGGVYLLPRRGPRLGVRATRKTSERSQPRGRRQP
**3aCON**	MSTLPKPQRKTKRNTIRRPQDVKFPGGGQIVGGVYVLPRRGPRLGVRATRKTSERSQPRGRRQP
**3bCON**	MSTLPKPQRQTKRNTPRRPQNVKFPGGGQIVGGVYVLPRRGPRLGVRAVRKTSERSQPRGRRQP
**3cCON**	MSTLPKPQRKTKRNTIRRPQDVKFPGGGQIVGGVYVLPRRGPLLGVRATRKTSERSQPRARRQP
**3dCON**	MSTLPKPQRKTKRNTIRRPQDVKFPGGGQIVGGVYLLPRRGPRLGVRAVRKTSERSEPRNRRQP
**3eCON**	MSTLPKPQRKTKRNTVRRPQDVKFPGGGQIVGGVYLLPRRGPRLGVRATRKTSERSQPRGRRQP
**3fCON**	MSTFPKPQRQPKRNTPRRPQDVKFPGGGQIVGGVYVLPRRGPRLGVRAVRKSSERSQPRGRRQP
**3gCON**	MSTLPKPQRQTKRNTPRRPQNVKFPGGGQIVGGVYVLPRRGPRLGVRAVRKTSERSQPRGRRQP
**3g2CON**	MSTLPKPQRQTKRNTPRRPQNVKFPGGGQIVGGVYVLPRRGPRLGVRAVRKTSERSQPRGRRQP
**3hCON**	MSTLPKPQRKTKRNTIRRPQNVKFPGGGQIVGGVYVLPRRGPTLGVRAVRKTSERSQPRGRRQP
**3iCON**	MSTLPKPQRQTKRNTPRRPQNVKFPGGGQIVGGVYVLPRRGPRLGVRAVRKTSERSQPRGRRQP
**3kCON**	MSTLPKPQRKTKRNTNRRPQDVKFPGGGQIVGGVYVLPRRGPKLGVRAVRKTSERSQPRSRRQP
**4aCON**	MSTNPKPQRKTKRNTNRRPMDVKFPGGGQIVGGVYLLPRRGPRLGVRATRKTSERSQPRGRRQP
**4bCON**	MSTNPKPQRKTKRNTNRRPMDVKFPGGGQIVGGVYLLPRRGPRLGVRATRKTSERSQPRGRRQP
**4b2CON**	MSTNPKPQRKTKRNTNRRPMDVKFPGGGQIVGGVYLLPRRGPRLGVRAARKTSERSQPRGRRQP
**4cCON**	MSTNPKPQRKTKRNTNRRPMDVKFPGGGQIVGGVYLLPRRGPRLGVRATRKTSERSQPRGRRQP
**4dCON**	MSTNPKPQRKTKRNTNRRPMDVKFPGGGQIVGGVYLLPRRGPRLGVRATRKTSERSQPRGRRQP
**4eCON**	MSTNPKLQRKTKRNTNRRPMDVKFPGGGQIVGGVYLLPRRGPRLGVRATRKTSERSQPRGRRQP
**4fCON**	MSTNPKPQRKTKRNTNRRPMDVKFPGGGQIVGGVYLLPRRGPRLGVRATRKTSERSQPRGRRQP
**4gCON**	MSTNPKPQRKTKRNTNRRPMDVKFPGGGQIVGGVYLLPRRGPRLGVRATRKTSERSQPRGRRQP
**4g2CON**	MSTNPKPQRKTKRNTNRRPMDVKFPGGGQIVGGVYLLPRRGPRLGVRATRKTSERSQPRGRRQP
**4hCON**	MSTNPKPQRKTKRNTNRRPMDVKFPGGGQIVGGVYLLPRRGPRLGVRATRKTSERSQPRGRRQP
**4kCON**	MSTNPKPQRKTKRNTNRRPMDVKFPGGGQIVGGVYLLPRRGPRLGVRATRKTSERSQPRGRRQP
**4lCON**	MSMNPKPQRKTHRNTNRRPMDVKFPGGGQIVGGVYLLPRRGPRLGVRATRKTSERSQPRGRRQP
**4l2CON**	MSTNPKPQRKTKRNTNRRPMDVKFPGGGQIVGGVYLLPRRGPRLGVRATRKTSERSQPRGRRQP
**4mCON**	MSTNPKPQRKTKRNTNRRPMDVKFPGGGQIVGGVYLLPRRGPRLGVRATRKTSERSQPRGRRQP
**4nCON**	MSTNPKPQRKTKRNTNRRPMDVKFPGGGQIVGGVYLLPRRGPRLGVRATRKTSERSQPRGRRQP
**4oCON**	MSTNPKPQRKTKRNTNRRPMDVKFPGGGQIVGGVYLLPRRGPRLGVRATRKTSERSQPRGRRQP
**4qCON**	MSTNPKPQRKTKRNTNRRPMDVKFPGGGQIVGGVYLLPRRGPRLGVRATRKTSERSQPRGRRQP
**4rCON**	MSTNPKPQRKTKRNTNRRPMDVKFPGGGQIVGGVYLLPRRGPRLGVRATRKTSERSQPRGRRQP
**4sCON**	MSTNPKPQRKTKRNTNRRPMDVKFPGGGQIVGGVYLLPRRGPRLGVRATRKTSERSQPRGRRQP
**4tCON**	MSTNPKPQRKTKRNTNRRPMDVKFPGGGQIVGGVYLLPRRGPRLGVRATRKTSERSQPRGRRQP
**4vCON**	MSTNPKPQRKTKRNTNRRPMDVKFPGGGQIVGGVYLLPRRGPRLGVRATRKTSERSQPRGRRQP
**5aCON**	MSTNPKPQRKTKRNTNRRPQDVKFPGGGQIVGGVYLLPRRGPRLGVRATRKTSERSQPRGRRQP
**6aCON**	MSTLPKPQRKTKRNTNRRPMDVKFPGGGQIVGGVYLLPRRGPRLGVRATRKTSERSQPRGRRQP
**6bCON**	MSTLPKPQRKTKRNTNRRPMDVKFPGGGQIVGGVYLLPRRGPRLGVRATRKTSERSQPRGRRQP
**6cCON**	MSTLPKPQRKTKRNTNRRPQDVKFPGGGQIVGGVYLLPRRGPRLGVRATRKTSERSQPRGRRQP
**6dCON**	MSTLPKPQKRNQRNTNRRPQDVKFPGGGQIVGGVYLLPRRGPRLGVRATRKTSERSQPRGRRQP
**6eCON**	MSTLPKPQRKTKRNTNRRPQDVKFPGGGQIVGGVYLLPRRGPRLGVRATRKTSERSQPRGRRQP
**6fCON**	MSTLPKPQRKTKRNTNRRPQDVKFPGGGQIVGGVYLLPRRGPRLGVRATRKTSERSQPRGRRQP
**6gCON**	MSTNPKPQRQTKRNTNRRPQDVKFPGGGQIVGGVYLLPRRGPRLGVRATRKTSERSQPRGRRQP
**6hCON**	MSTLPKPQRKTKRNTNRRPMDVKFPGGGQIVGGVYLLPRRGPRLGVRATRKTSERSQPRGRRQP
**6iCON**	MSTLPKPQRKTKRNTNRRPMDVKFPGGGQIVGGVYLLPRRGPRLGVRATRKTSERSQPRGRRQP
**6jCON**	MSTLPKPQRKTKRNTNRRPMDVKFPGGGQIVGGVYLLPRRGPRLGVRATRKTSERSQPRGRRQP
**6kCON**	MSTLPKPQRKTKRNTNRRPMDVKFPGGGQIVGGVYLLPRRGPRLGVRATRKTSERSQPRGRRQP
**6lCON**	MSTLPKPQRKTKRNTNRRPMDVKFPGGGQIVGGVYLLPRRGPRLGVRATRKTSERSQPRGRRQP
**6l2CON**	MSTLPKPQRKTKRNTNRRPMDVKFPGGGQIVGGVYLLPRRGPRLGVRATRKTSERSQPRGRRQP
**6mCON**	MSTLPKPQRQTKRNTNRRPQDVKFPGGGQIVGGVYLLPRRGPRLGVRAVRKTSERSQPRGRRQP
**6nCON**	MSTLPKPQRKTKRNTNRRPMDVKFPGGGQIVGGVYLLPRRGPRLGVRATRKTSERSQPRGRRQP
**6oCON**	MSTLPKPQRKTKRNTTRRPQDVKFPGGGQIVGGVYLLPRRGPRLGVRATRKTSERSQPRGRRQP
**6pCON**	MSTNPKPQRKTKRNTNRRPQDVKFPGGGQIVGGVYLLPRRGPRLGVRATRKTSERSQPRGRRQP
**6qCON**	MSTLPKPQRKTKRNTNRRPQDVKFPGGGQIVGGVYLLPRRGPRLGVRATRKTSERSQPRGRRQP
**6rCON**	MSTLPKPQRKTNRNTNRRPQDVKFPGGGQIVGGVYLLPRRGPRLGVRATRKTSERSQPRGRRQP
**6r2CON**	MSTLPKPQRKTKRNTNRRPQDVKFPGGGQIVGGVYLLPRRGPRLGVRATRKTSERSQPRGRRQP
**6tCON**	MSTLPKPQRPTKRNTNRRPKDVKFPGGGQIVGGVYLLPRRGPRLGVRATRKTSERSQPRGRRQP
**6uCON**	MSTQPKPQRLTKRNTNRRPQNVKFPGGGQIVGGVYLLPRRGPRLGVRAVRKTSERSQPRGRRQP
**6vCON**	MSTSSKPQRQTKRNTNRRPQDVKFPGGGQIVGGVYLLPRRGPRLGVRAVRKTSERSQPRGRRQP
**6wCON**	MSTLPKPQRKTKRNTNRRPQDVKFPGGGQIVGGVYLLPRRGPRLGVRATRKTSERSQPRGRRQP
**6w2CON**	MSTLPKPQRKTKRNTNRRPQDVKFPGGGQIVGGVYLLPRRGPRLGVRATRKTSERSQPRGRRQP

```
             70        80        90       100       110       120
1aCON    IPKARRPEGRTWAQPGYPWPLYGNEGCGWAGWLLSPRGSRPSWGPTDPRRRSRNLGKVIDTLTC
1bCON    IPKARRPEGRAWAQPGYPWPLYGNEGMGWAGWLLSPRGSRPSWGPTDPRRRSRNLGKVIDTLTC
1cCON    IPKARRPEGRSWAQPGYPWPLYGNEGCGWAGWLLSPRGSRPSWGPSDPRRRSRNLGKVIDTLTC
1eCON    IPKARRPEGRSWAQPGYPWPLYGNEGCGWAGWLLSPRGSRPSWGPSDPRRRSRNLGKVIDTLTC
1e2CON   IPKARRPEGRSWAQPGYPWPLYGNEGCGWAGWLLSPRGSRPSWGPSDPRRRSRNLGKVIDTLTC
1gCON    IPKARRPEGRTWAQPGYPWPLYGNEGCGWAGWLLSPRGSRPSWGPSDPRRRSRNLGKVIDTLTC
1hCON    IPKARRPEGRTWAQPGYPWPLYGNEGCGWAGWLLSPRGSRPSWGPSDPRRRSRNLGKVIDTLTC
1lCON    IPKARQPEGRSWAQPGYPWPLYGNEGCGWAGWLLSPRGSRPSWGPTDPRRRSRNLGKVIDTLTC
2aCON    IPKDRRSTGKSWGKPGYPWPLYGNEGLGWAGWLLSPRGSRPSWGPNDPRHRSRNVGKVIDTLTC
2bCON    IPKDRRSTGKSWGKPGYPWPLYGNEGCGWAGWLLSPRGSRPTWGPTDPRHRSRNLGKVIDTITC
2cCON    IPKDRRTTGKSWGRPGYPWPLYGNEGLGWAGWLLSPRGSRPSWGPTDPRHRSRNLGKVIDTLTC
2dCON    IPKDRRPTGKSWGKPGYPWPLYGNEGLGWAGWLLSPRGSRPSWGPTDPRHRSRNLGKVIDTLTC
2eCON    IPKDRRNTGKSWGRPGYPWPLYGNEGLGWAGWLLSPRGSRPSWGPTDPRHKSRNLGKVIDTLTC
2fCON    IPKDRRSTGKSWGRPGYPWPLYGNEGLGWAGWLLSPRGSRPSWGPTDPRHKSRNLGKVIDTLTC
2f2CON   IPKDRRSTGKSWGRPGYPWPLYGNEGLGWAGWLLSPRGSRPSWGPTDPRHRSRNLGKVIDTLTC
2iCON    IPKDRRTTGKSWGRPGYPWPLYGNEGLGWAGWLLSPRGSRPSWGPTDPRHRSRNLGKVIDTLTC
2jCON    IPKDRRSTGKSWGRPGYPWPLYGNEGLGWAGWLLSPRGSRPSWGPTDPRHRSRNLGKVIDTLTC
2kCON    IPKDRRSTGKSWGRPGYPWPLYGNEGLGWAGWLLSPRGSRPSWGPTDPRHRSRNLGKVIDTLTC
2lCON    IPKVRRTTGRSWQQPGYPWPLYGNEGLGWAGWLLSPQGSRPSWGPTDPRRRSRNLGKVIDTLTC
2l2CON   IPKVRRSTGRSWQQPGYPWPLYGNEGLGWAGWLLSPQGSRPSWGPTDPRRRSRNLGKVIDTLTC
2mCON    IPKDRRSTGKSWGKPGYPWPLYGNEGLGWAGWLLSPRGSRPSWGPTDPRHKSRNLGKVIDTLTC
2qCON    IPKDRRATGRSWGRPGYPWPLYGNEGLGWAGWLLSPRGSRPSWGPTDPRHRSRNLGKVIDTLTC
2q2CON   IPKDRRSTGKSWGRPGYPWPLYGNEGLGWAGWLLSPRGSRPSWGPTDPRHRSRNLGKVIDTLTC
3aCON    IPKARRSEGRSWAQPGYPWPLYGNEGCGWAGWLLSPRGSRPSWGPNDPRRRSRNLGKVIDTLTC
3bCON    IPKARPREGRSWAQPGYPWPLYGNEGCGWAGWLLSPRGSRPSWGPNDPRRRSRNLGKVIDTLTC
3cCON    IPKARRGEGRSWAEPGYPWPLYGNEGCGWAGWLLSPRGSRPSWGPNDPRRRSRNLGKVIDTLTC
3dCON    IPKARRSEGRSWAQPGYPWPLYGNEGCGWAGWLLSPRGSRPSWGPNDPRRRSRNLGKVIDTLTC
3eCON    IPKARPSGGRSWAQPGYPWPLYGNEGCGWAGWLLSPRGSRPSWGPNDPRRRSRNLGKVIDTLTC
3fCON    IPKARRSEGRSWAQPGYPWPLYGNEGCGWAGWLLSPRGSRPSWGPNDPRRRSRNLGKVIDTLTC
3gCON    IPKARQREGRSWAQPGYPWPLYGNEGCGWAGWLLSPRGSRPSWGPNDPRRRSRNLGKVIDTLTC
3g2CON   IPKARQREGRSWAQPGYPWPLYGNEGCGWAGWLLSPRGSRPSWGPNDPRRRSRNLGKVIDTLTC
3hCON    IPKARRNEGRTWAQPGYPWPLYGNEGCGWAGWLLSPRGSRPHWGPNDPRRRSRNLGKIIDTLTC
3iCON    IPKARQSEGRSWAQPGYPWPLYGNEGCGWAGWLLSPRGSRPSWGPNDPRRRSRNLGKVIDTLTC
3kCON    IPRARRTEGRSWAQPGYPWPLYGNEGCGWAGWLLSPRGSRPSWGPNDPRRRSRNLGKVIDTLTC
4aCON    IPKARRSEGRSWAQPGYPWPLYGNEGCGWAGWLLSPRGSRPSWGPNDPRRRSRNLGKVIDTLTC
4bCON    IPKARRSEGRSWAQPGYPWPLYGNEGCGWAGWLLSPRGSRPSWGPNDPRRRSRNLGKVIDTLTC
4b2CON   IPKARRSEGRSWAQPGYPWPLYGNEGCGWAGWLLSPRGSRPSWGPNDPRRRSRNLGKVIDTLTC
4cCON    IPKARRSEGRSWAQPGYPWPLYGNEGCGWAGWLLSPRGSRPSWGPNDPRRRSRNLGKVIDTLTC
4dCON    IPKARQPEGRSWAQPGYPWPLYGNEGCGWAGWLLSPRGSRPSWGPNDPRRRSRNLGKVIDTLTC
4eCON    IPKARRSEGRSWAQPGYPWPLYGNEGCGWAGWLLSPRGSRPSWGPNDPRRRSRNLGKVIDTLTC
4fCON    IPKARRTEGRSWAQPGYPWPLYGNEGCGWAGWLLSPRGSRPSWGPNDPRRRSRNLGKVIDTLTC
4gCON    IPKARRSEGRAWAQPGYPWPLYGNEGCGWAGWLLSPRGSRPSWGPNDPRRRSRNLGKVIDTLTC
4g2CON   IPKARPSEGRSWAQPGYPWPLYGNEGCGWAGWLLSPRGSRPSWGPNDPRRRSRNLGKVIDTLTC
4hCON    IPKARPSEGRSWAQPGYPWPLYGNEGCGWAGWLLSPRGSRPSWGPNDPRRRSRNLGKVIDTLTC
4kCON    IPKARRSEGRSWAQPGYPWPLYGNEGCGWAGWLLSPRGSRPSWGPNDPRRRSRNLGKVIDTLTC
4lCON    IPKARRPEGRSWAQPGYPWPLYGNEGCGWAGWLLSPRGSRPSWGPNDPRRRSRNLGKVIDTLTC
4l2CON   IPKARQPEGRSWAQPGYPWPLYGNEGCGWAGWLLSPRGSRPSWGPNDPRRRSRNLGKVIDTLTC
4mCON    IPKARRPEGRTWAQPGYPWPLYGNEGCGWAGWLLSPRGSRPSWGPNDPRRRSRNLGKVIDTLTC
4nCON    IPKARRSEGRSWAQPGYPWPLYGNEGCGWAGWLLSPRGSRPSWGPNDPRRRSRNLGKVIDTLTC
4oCON    IPKARRPEGRTWAQPGYPWPLYGNEGCGWAGWLLSPRGSRPSWGPNDPRRRSRNLGKVIDTLTC
4qCON    IPKARRPEGRSWAQPGYPWPLYGNEGCGWAGWLLSPRGSRPSWGPNDPRRRSRNLGKVIDTLTC
4rCON    IPKARRPEGRSWAQPGYPWPLYGNEGCGWAGWLLSPRGSRPSWGPNDPRRRSRNLGKVIDTLTC
4sCON    IPKARRPEGRSWAQPGYPWPLYGNEGCGWAGWLLSPRGSRPSWGPNDPRRRSRNLGKVIDTLTC
4tCON    IPKARRPEGRSWAQPGYPWPLYGNEGCGWAGWLLSPRGSRPSWGPNDPRRRSRNLGKVIDTLTC
4vCON    IPKARRPEGRSWAQPGYPWPLYGNEGCGWAGWLLSPRGSRPSWGPNDPRRRSRNLGKVIDTLTC
5aCON    IPKARQPTGRSWGQPGYPWPLYANEGLGWAGWLLSPRGSRPNWGPNDPRRKSRNLGKVIDTLTC
6aCON    IPKARQPQGRHWAQPGYPWPLYGNEGCGWAGWLLSPRGSRPHWGPNDPRRRSRNLGKVIDTLTC
6bCON    IPKARPSQGRTWGQPGYPWPLYGNEGCGWAGWLMSPRGSRPSWGPNDPRRRSRNLGKVIDTLTC
6cCON    IPKARHQTGRAWAQPGYPWPLYGNEGCGWAGWLLSPRGSRPNWGPNDPRRRSRNLGKVIDTLTC
6dCON    IPKARRQTGRTWAQPGYPWPLYGNEGCGWMGWLLSPRGSRPHWGPNDPRRRSRNLGKVIDTLTC
6eCON    IPKVRHQTGRTWAQPGYPWPLYGNEGCGWAGWLLSPRGSRPNWGPNDPRRRSRNLGKVIDTLTC
6fCON    IPKARHQTGRSWAQPGYPWPLYGNEGCGWAGWLLSPRGSRPHWGPNDPRRRSRNLGKVIDTLTC
6gCON    IPKARRQTGRAWGQPGYAWPLYGNEGCGWAGWLLSPRGSRPTWGPNDPRRRSRNLGKVIDTLTC
6hCON    IPKARQPIGRSWGQPGYPWPLYGNEGCGWAGWLLSPRGSRPNWGPNDPRRRSRNLGKVIDTLTC
6iCON    IPKARPPTGRSWGQPGYPWPLYGNEGCGWAGWLLSPRGSRPNWGPNDPRRRSRNLGKVIDTLTC
6jCON    IPKARRPTGRSWAQPGYPWPLYGNEGCGWAGWLLSPRGSRPSWGPNDPRRRSRNLGKVIDTLTC
6kCON    IPKARQSQGRHWAQPGYPWPLYGNEGCGWAGWLLSPRGSRPNWGPNDPRRRSRNMGKVIDSLTC
6lCON    IPKARQPTGRHWAQPGYPWPLYGNEGCGWAGWLLSPRGSRPNWGPNDPRRRSRNLGKVIDTITC
6l2CON   IPKARQPTGRHWAQPGYPWPLYGNEGCGWAGWLLSPRGSRPNWGPNDPRRRSRNLGKVIDTITC
6mCON    IPKARQPTGRHWAQPGYPWPLYGNEGCGWAGWLLSPRGSRPNWGPNDPRRRSRNLGKVIDTLTC
6nCON    IPKARQPTGRHWAQPGYPWPLYGNEGCGWAGWLLSPRGSRPNWGPNDPRRRSRNLGKVIDTITC
6oCON    IPKVRHQTGRTWAQPGYPWPLYGNEGCGWAGWLLSPRGSRPNWGPNDPRRRSRNLGKVIDTLTC
6pCON    IPKDRRATGKSWGRPGYPWPLYGNEGLGWAGWLLSPRGSRPSWGPTDPRHRSRNLGKVIDTLTC
6qCON    IPTARHQSGRSWAQPGYPWPLYGNEGCGWAGWLLSPRGSRPHWGPNDPRRRSRNLGKVIDTLTC
6rCON    IPKARHQTGRSWAQPGYPWPLYGNEGCGWAGWLLSPRGSRPHWGPNDPRRRSRNLGKVIDTLTC
6r2CON   IPKARHQTGRSWAQPGYPWPLYGNEGCGWAGWLLSPRGSRPHWGPNDPRRRSRNLGKVIDTLTC
6tCON    IPKARRQTGRTWAQPGYPWPLYGNEGCGWAGWLLSPRGSRPHWGPNDPRRRSRNLGKVIDTLTC
6uCON    IPKQRPPTGRHWAQPGYPWPLYGNEGCGWAGWLLSPRGSRPHWGPNDPRRRSRNLGKVIDTLTC
6vCON    IPKARQPTGRRWAQPGYPWPLYGNEGCGWAGWLLSPRGSRPHWGPNDPRRRSRNLGKVIDTFTC
6wCON    IPKARQPAGRTWGQPGYPWPLYGNEGCGWAGWLLSPRGSRPTWGPNDPRRRSRNLGKVIDTFTC
6w2CON   IPKVRHATGRTWGQPGYPWPLYGNEGCGWAGWLLSPRGSRPTWGPNDPRRRSRNLGKVIDTFTC
```

```
             130       140       150       160       170       180       190
1aCON     GFADLMGYIPLVGAPLGGAARALAHGVRVLEDGVNYATGNLPGCSFSIFLLALLSCLTVPASA
1bCON     GFADLMGYIPLVGAPLGGAARALAHGVRVLEDGVNYATGNLPGCSFSIFLLALLSCLTIPASA
1cCON     GFADLMGYIPLVGAPLGGAARALAHGVRVLEDGVNYATGNLPGCSFSIFLLALLSCLTVPASA
1eCON     GFADLMGYIPLVGAPLGGAARALAHGVRVLEDGVNYATGNLPGCSFSIFLLALLSCLTVPASA
1e2CON    GFADLMGYIPLVGAPLGGAARALAHGVRVLEDGVNYATGNLPGCSFSIFLLALLSCLTVPASA
1gCON     GFADLMGYIPLVGAPLGGAARALAHGVRVLEDGVNYATGNLPGCSFSIFLLALLSCLTVPASA
1hCON     GFADLMGYIPLVGAPLGGAARALAHGVRVLEDGVNYATGNLPGCSFSIFLLALLSCLTVPASA
1lCON     GFADLMGYIPLVGAPLGGAARALAHGVRVLEDGVNYATGNLPGCSFSIFLLALLSCLTVPASA
2aCON     GFADLMGYIPVVGAPLGGVARALAHGVRVLEDGVNYATGNLPGCSFSIFLLALLSCITVPVSA
2bCON     GFADLMGYIPVVGAPVGGVARALAHGVRVLEDGINYATGNLPGCSFSIFLLALLSCVTVPVSA
2cCON     GFADLMGYIPVVGAPVGGVARALAHGVRVLEDGINYATGNLPGCSFSIFLLALLSCISVPVSA
2dCON     GFADLMGYIPVVGAPVGGVARALAHGVRVLEDGINYATGNLPGCSFSIFLLALLSCITVPVSG
2eCON     GFADLMGYIPVVGAPVGGVARALAHGVRVLEDGINYATGNLPGCSFSIFLLALLSCITVPTSA
2fCON     GFADLMGYIPVVGAPVGGVARALAHGVRVLEDGINYATGNLPGCSFSIFLLTLLSCITVPVSA
2f2CON    GFADLMGYIPVVGAPVGGVARALAHGVRVLEDGINYATGNLPGCSFSIFLLALLSCITAPVSA
2iCON     GFADLMGYIPVVGAPVGGVARALAHGVRVLEDGINYATGNLPGCSFSIFLLALLSCITVPVSA
2jCON     GFADLMGYIPVVGAPVGGVARALAHGVRVLEDGINYATGNLPGCSFSIFLLALLSCISIPVSA
2kCON     GFADLMGYIPVVGAPVGGVARALAHGVRVLEDGINYATGNLPGCSFSIFLLALLSCISVPVSA
2lCON     GFADLVGYIPVVGGPVGGLARALAHGVRVVEDGINYATGNLPGCSFSIFLLALFSCITVPASA
2l2CON    GFADLMGYIPVVGGPVGGLARALAHGVRVLEDGVNYATGNLPGCSFSIFLLALFSCITVPASA
2mCON     GFADLMGYIPVVGAPVGGVARALAHGVRVLEDGINYATGNLPGCSFSIFLLALLSCLTVPVSS
2qCON     GFADLMGYIPVVGAPVGGVARALAHGVRVLEDGINYATGNLPGCSFSIFLLALLSCMSVPVSA
2q2CON    GFADLMGYIPVVGAPVGGVARALAHGVRVLEDGINYATGNLPGCSFSIFLLALLSCMSVPVSA
3aCON     GFADLMGYIPLVGAPVGGVARALAHGVRALEDGINFATGNLPGCSFSIFLLALFSCLIHPAAS
3bCON     GFADLMGYIPLIGAPVGGVARALAHGVRALEDGVNYATGNLPGCSFSIFLLALFSCLTCPASG
3cCON     GFADLMGYIPLVGAPVGGVARALAHGVRALEDGINFATGNLPGCSFSIFLLALFSCLVCPAAG
3dCON     GFADLMGYIPLVGAPVGGVARALAHGVRALEDGINFATGNLPGCSFSIFLLALLSCLVCPAAG
3eCON     GFADLMGYIPLVGAPVGGVARALAHGVRALEDGINFATGNLPGCSFSIFLLALLSCLLCPAVG
3fCON     GFADLMGYIPLVGAPVGGVARALAHGVRALEDGINFATGNLPGCSFSIFLLALFSCLVSPAAG
3gCON     GFADLMGYIPLVGAPVGGVARALAHGVRALEDGINFATGNLPGCSFSIFLLALLSCLTCPAAG
3g2CON    GFADLMGYIPLVGAPAGGVARALAHGVRALEDGINFATGNLPGCSFSIFLLALFSCLTCPTAA
3hCON     GFADLMGYIPLVGAPVGGVARALAHGVRAVEDGINYATGNLPGCSFSIFLLAILSCLTVPASG
3iCON     GFADLMGYIPLVGAPVGGVARALAHGVRALEDGINFATGNLPGCAFSIFLLALFSCLTCPATG
3kCON     GFADLMGYIPLVGAPVGGVARALAHGVRALEDGINFATGNLPGCSFSIFLLALLSCLLTPTAG
4aCON     GFADLMGYIPLVGAPVGGVARALAHGVRAVEDGINYATGNLPGCSFSIFLLALLSCLTVPASA
4bCON     GFADLMGYIPLVGAPVGGVARALAHGVRAVEDGINYATGNLPGCSFSIFLLALLSCLTTPASA
4b2CON    GFADLMGYIPLVGAPVGGVARALAHGVRAVEDGINYATGNLPGCSFSIFLLALLSCLTTPASA
4cCON     GFADLMGYIPLVGAPVGGVARALAHGVRAVEDGINYATGNLPGCSFSIFLLALLSCLTVPTSA
4dCON     GFADLMGYIPVVGAPVGGVARALAHGVRLLEDGVNYATGNLPGCSFSIFLLALLSCLTVPASA
4eCON     GFADLMGYIPLVGAPVGGVARALAHGVRAVEDGINYATGNLPGCSFSIFLLALLSCLTVPASG
4fCON     GFADLMGYIPLVGAPVGGVARALAHGVRAVEDGINYATGNLPGCSFSIFLLALLSCLTVPASA
4gCON     GFADLMGYIPLVGAPVGGVARALAHGVRAVEDGINYATGNLPGCSFSIFLLALLSCLTVPASA
4g2CON    GFADLMGYIPLVGAPVGGVARALAHGVRAVEDGINYATGNLPGCAFSIFLLALLSCLTVPASA
4hCON     GFADLMGYIPLVGAPVGGVARALAHGVRALEDGINYATGNLPGCAFSIFLLALLSCLTVPASA
4kCON     GFADLMGYIPLVGAPVGGVARALAHGVRALEDGINYATGNLPGCSFSIFILALLSCLTVPASA
4lCON     GFADLMGYIPLVGAPVGGVARALAHGVRALEDGINYATGNLPGCSFSIFLLALLSCLTVPASA
4l2CON    GFADLMGYIPVVGAPVGGVARALAHGVRALEDGINYATGNLPGCSFSIFLLALLSCLTVPASA
4mCON     GFADLMGYIPLVGAPVGGVARALAHGVRALEDGVNYATGNLPGCSFSIFLLALLSCLTVPASA
4nCON     GFADLMGYIPLVGAPVGGVARALAHGVRAVEDGINYATGNLPGCSFSIFLLALLSCLTVPASA
4oCON     GFADLMGYIPLVGAPVGGVARALAHGVRALEDGINYATGNLPGCSFSIFLLALLSCLTVPTSA
4qCON     GFADLMGYIPLVGAPVGGVARALAHGVRALEDGINYATGNLPGCSFSIFLLALLSCLTVPASA
4rCON     GFADLMGYIPLVGAPVGGVARALAHGVRAVEDGINYATGNLPGCSFSIFLLALLSCLTVPASA
4sCON     GFADLMGYIPIVGAPVGGVARALAHGVRAVEDGINYATGNLPGCSFSIFLLALLSCLTVPASA
4tCON     GFADLMGYIPLVGAPVGGVARALAHGVRAVEDGINYATGNLPGCSFSIFLLALLSCLTVPTSA
4vCON     GFADLMGYIPLVGAPVGGVARALAHGVRALEDGINYATGNLPGCSFSIFLLALLSCLTVPASA
5aCON     GFADLMGYIPLVGGPVGGVARALAHGVRVLEDGVNYATGNLPGCSFSIFILALLSCLTVPASA
6aCON     GFADLMGYIPVVGAPLGGVAAALAHGVRAIEDGINYATGNLPGCSFSIFLLALLSCLTTPASA
6bCON     GLADLMGYIPVVGGPLGGVAAALAHGVRAIEDGINYATGNLPGCSFSIFILALLSCLTTPASA
6cCON     GFADLMGYIPVVGAPLGGVAAALAHGVRAIEDGINYATGNLPGCSFSIFLLALLSCLTTPASA
6dCON     GFADLMGYIPVVGAPLGGVAAALAHGVRAVEDGINYATGNLPGCSFSIFLLALLSCLTTPASA
6eCON     GFADLMGYIPVVGAPLGGIAAALAHGVRAVEDGINYATGNLPGCSFSIFLLALLSCLTTPASA
6fCON     GFADLMGYIPVVGAPLGGVAAALAHGVRAIEDGINYATGNLPGCSFSIFLLALLSCLTTPASA
6gCON     GLADLMGYIPVIGGPLGGVAAALAHGVRAVEDGVNYATGNLPGCSFSIFLLALLSCLTVPASA
6hCON     GLADLMGYIPVLGGPLGGVAAALAHGVRAIEDGVNYATGNLPGCSFSIFLLALLSCLTTPASA
6iCON     GLADLMGYIPVLGGPLGGVAAALAHGVRAIEDGVNYATGNLPGCSFSIFILALLSCLTTPVSA
6jCON     GLADLMGYIPVLGGPLGGVAAALAHGVRAIEDGVNYATGNLPGCSFSIFILALLSCLTTPASA
6kCON     GFADLMGYIPVVGAPLGGVAAALAHGVRAIEDGINYATGNLPGCSFSIFLLALLSCLTTPASA
6lCON     GVADLMGYIPVLGAPLGGVAAALAHGVRAIEDGINYATGNLPGCSFSIFLLALLSCLTTPASA
6l2CON    GFADLMGYIPVLGAPLGGVAAALAHGVRAIEDGINYATGNLPGCSFSIFLLALLSCLTTPASA
6mCON     DLADLMGYIPVLGAPLGGVAAALAHGVRAIEDGINYATGNLPGCSFSIFLLALLSCLTTPASA
6nCON     GLADLMGYIPVLGAPLGGVAAALAHGVRAVEDGINYATGNLPGCSFSIFLLALLSCLTTPASA
6oCON     GFADLMGYIPVVGAPLGGVAAALAHGVRAIEDGINYATGNLPGCSFSIFLLALLSCLTTPASA
6pCON     GFADLMGYIPVVGAPVGGVARALAHGVRVLEDGINYATGNLPGCSFSIFLLALLSCITVPVSA
6qCON     GFADLMGYIPVVGAPLGGVAAALAHGVRALEDGINYATGNLPGCSFSIFLLALLSCLTVPASA
6rCON     GFADLMGYIPVVGAPLGGVAAALAHGVRTIEDGINYATGNLPGCSFSIFLLALLSCLTTPASA
6r2CON    GFADLMGYIPVVGAPLGGVAAALAHGVRTIEDGINYATGNLPGCSFSIFLLALLSCLTTPASA
6tCON     GFADLMGYIPVVGAPLGGVAAALAHGVRAIEDGINYATGNLPGCSFSIFLLALLSCLTTPASA
6uCON     GFADLMGYIPVIGAPLGGVAAALAHGVRAIEDGINYATGNLPGCSFSIFLLALLSCLTVPASA
6vCON     GFADLMGYIPVVGAPLGGVAAALAHGVRAIEDGINYATGNLPGCSFSIFLLALLSCLTTPASA
6wCON     GLADLMGYIPVLGGPLGGVAAALAHGVRVVEDGINYATGNLPGCSFSILLLALLSCLTVPTSA
6w2CON    GLADLMGYIPVLGGPLGGVAAAFAHGVRAVEDGINYATGNLPGCSFSIFLLALLSCLTVPTSA
```

HCV NS3

```
        1        10        20        30        40        50        60        70        80        90
1aCON   APITAYAQQTRGLLGCIITSLTGRDKNQVEGEVQIVSTAAQTFLATCINGVCWTVYHGAGTRTIASPKGPVIQMYTNVDQDLVGWPAPQGA
1bCON   APITAYSQQTRGLLGCIITSLTGRDKNQVEGEVQVVSTATQSFLATCVNGVCWTVYHGAGSKTLAGPKGPITQMYTNVDQDLVGWQAPPGA
1cCON   APITAYAQQTRGLLGCIITSLTGRDKNQVEGEVQIVSTATQTFLATCVNGVCWTVYHGAGSRTIASASGPVVQMYTNVDQDLVGWPAPQGA
1eCON   APITAYAQQTRGLLGCIVTSLTGRDRNTVEGEIQILSTATQTFLGTTVNGVVWSVYHGAGTKFLAGLKGPVTQMYTNVDQDLVGWQAPSGA
1gCON   APITAYAQQTRGLMGCIVTSLTGRDKNTVEGEVQIVSTANQTFLATCVNGVLWSVYHGAGTRFLAARKGPVAQMYTNVDQDLVGWQAPAGA
1lCON   APITAYAQQTRGLLGCIITSLTGRDKNQVEGEVQIVSTATQTFLATCVNGACWTVYHGAGSRNLAGPKGPIIQMYTNVDLDLVGWQAPQGA
2aCON   APITAYAQQTRGLLGAIVVSMTGRDKTEQAGEIQVLSTVTQSFLGTSISGVLWTVYHGAGNKTLAGSRGPVTQMYSSAEGDLVGWPSPPGT
2bCON   APITAYTQQTRGLLGAIVVSLTGRDKNEQAGQVQVLSSVTQSFLGTSISGVLWTVYHGAGNKTLAGPKGPVTQMYTSAEGDLVGWPSPPGT
2cCON   APITAYAQQTRGLLGAIVVSMTGRDKTDQAGEIQVLSTVTQSFLGTSISGVLWTVFHGAGNKTLAGSRGPVTQMYSSAEGDLVGWPSPPGT
2fCON   APITAYTQQTRGLLGTIVVSLTGRDRTAQAGEVQVLSTVTQTFLGTTISGVLWTVFHGAGNKTLAGPRGPVTQMYTSAEGDLVGWSSPPGT
2iCON   APITAYAQQTRGLLGAIVVSLTGRDKTEQAGEVQVLSTITQSFLGTTISGVLWTVFHGAGNKTLAGSRGPVTQMYTSAEGDLVGWPSPPGT
2jCON   APITAYAQQTRGLLGTIVVSLTGRDKTECAGEVQVLSTVTQSFLGTSISGVLWTVFHGAGNKTLAGSRGPVTQMYTSAEGDLVGWPSPPGT
2kCON   APITAYAQQTRGLLGTIVVSLTGRDRTEVEGEIQVLSTATQSFLGTTISGVLWTVYHGAGNKTLAGPKGPITQMYTSAEGDLVGWPAPPGA
2lCON   APITAYAQQTRGVLGAIVVSLTGRDKTDHAGEVQILSTVTQTFLGTCVSGVMWTVYHGAGSKTLAGPKGPILQMYTGAEGDLAGWPAPPGA
2mCON   APITAYTQQTRGVLGAIVVSLTGRDKTEQAGEIQVLSTVTQSFLGTTISGVLWTVFHGAGNKTLAGSRGPVTQMYSSAEGDLVGWPSPPGT
3aCON   APITAYAQQTRGLLGTIVTSLTGRDKNVVTGEVQVLSTATQTFLGTTVGGVMWTVYHGAGSRTLAGAKHPALQMYTNVDQDLVGWPAPPGA
3bCON   SPISAYAQQTRGLFGTIVTSLTGRDKNVVTGEVQVLSTATQTFLGTTVGGVMWTVYHGAGSRTLAGNKRPALQMYTNVDQDLVGWPAPAGT
3gCON   SPITAYAQQTRGLFGTIVTSLTGRDKNVVTGEVQVLSTATQTFLGTTVGGVMWTVYHGAGSRTLASSKRPALQMYTNVDQDLVGWPAPAGA
3iCON   SPITAYAQQTRGLLGTIVTSLTGRDNNIVTGEVQVLSTTTQTFLGTTVGGVMWTVYHGAGSRSLAASKRPALQMYTNVEQDLVGWPAPAGT
3kCON   APITAHAQQTRGLFGTIVTSLTGRDKNIVTGEIQVLSTSTQTFLGTSVGGVMWTVYHGAGSRTLAGNKRPALQMYTNVDQDLVGWPSPPGA
4aCON   APITAYAQQTRGLFSTIITSLTGRDTNENCGEVQVLSTATQSFLGTAVNGVMWTVYHGAGSKTISGPKGPVNQMYTNVDQDLVGWPAPPGV
4dCON   APITAYAQQTRGMLGTIITSLTGRDTNENCGEIQVLSTATQSFLGTAINGVMWTVYHGAGSKTISGPKGPVNQMYTNVDQDLVGWPAPPGV
4fCON   APITAYAQQTRGLLGTIVTSLTGRDTNENCGEIQVLSTATQSFLGTAVNGVMWTVYHGAGSKTLSGPKGPVNQMYTNVDQDLVGWPAPPGV
4gCON   APITAYAQQTRGLLGTIVTSLTGRDTNENCGEVQVLSTATQTFLGTGISGVMWTVYHGAGSKTICGPKGPVCQMYTNVDQDLVGWPAPPGV
4kCON   APITAYAQQTRGLLSTIVTSLTGRDTNENRGEIQVLSTATQTFLGTGINGVMWTVYHGAGSKTICGPKGPVCQMYTNVDQDLVGWPAPPGV
4lCON   APITAYAQQTRGLLGTIVTSLTGRDTNENCGEVQVLSTTTQSFLGTAVNGVMWTVYHGAGSKTISGPKGPVNQMYTNVDQDLVGWPAPPGV
4mCON   APITAYAQQTRGLLSTIVTSLTGKDTNENCGEVQVLSTATQSFLGTAVNGVMWTVYHGAGGKTISGPKGPVCQMYTNVDQDLVGWPAPPGV
4nCON   APITAYAQQTRGLCSTIVTSLTGRDTNDNCGEVQVLSTATQSFLGTAVNGVMWTVYHGAGSKTLCGPKGPVCQMYTNVDQDLVGWPAPPGV
4oCON   APITAYAQQTRGLFSTIVTSLTGRDTNANCGEVQVLSTATQSFLGTAINGVMWTVYHGAGSKTICGPKGPVNQMYTNVDQDLVGWPAPPGV
4pCON   APITAYAQQTRGLLSTIVTSLTGRDTNENCGEVQVLSTATQSFLGTAVNGVMWTVYHGAGSKTISGPKGPVNQMYTNVDQDLVGWPAPPGV
4qCON   APITAYAQQTRGLLGTIVTSLTGRDKNENCGEVQVLSTATQSFLGTAVNGVMWTVYHGAGSKTICGPKGPVNQMYTNVDQDLVGWPAPPGV
4rCON   APITAYAQQTRGLLGTIVTSLTGRDKNENCGEVQVLSTATQTFLGTGINGVMWTVYHGAGSKTICGPKGPVSQMYTNVDQDLVGWPAPPGV
4sCON   APITAYAQQTRGLFSTIVTSLTGRDTNENRGEVQVLSTATQSFLGTAINGVMWTVYHGAGSKTISGPRGPVSQMYTNVDQDLVGWPAPSGV
4vCON   APITAYAQQTRGLVSTIVTSLTGRDTNENCGEVQVLSTATQSFLGTAVNGVMWTVYHGAGSKTICGPKGPVNQMYTNVDQDLVGWPAPPGV
5aCON   APITAYAQQTRGVLGAIVVSLTGRDKNEAEGEVQVLSTATQTFLGTCINGVMWTVFHGAGSKTLAGPKGPVVQMYTNVDKDLVGWPSPPGT
6aCON   APITAYAQQTRGLVGTIVTSLTGRDKNEVEGEVQVVSTATQSFLATSINGVMWTVYHGAGSKTLAGPKGPVCQMYTNVDKDLVGWPSPPGA
6bCON   APITAYAQQTRGLVGTIVTSLTGRDKNEAEGEVQVVSTATQSFLATTINGVLWTVYHGAGSKNLAGPKGPVCQMYTNVDQDLVGWPAPLGA
6dCON   APITAYCQQTRGLLGTIVTSLTGRDRNVVEGEIQVLSTATQSFLGTAINGVMWTVYHGAGSKTLAGPKGPVCQMYTNVDQDMVGWPAPPGT
6eCON   .......QQTRGLLGTIVTSLTGRDKNEVEGEIQVVSTATQTFLATAINGVLWTVYHGAGSKTLAGPKGPVCQMYTNVDQDMVGWPAPPGA
6fCON   APITAYHQQTRGLLGTIVTSLTGRDKNEAEGEVQVLSTATQTFLASCINGVLWTVYHGAGSKTLAGPKGPVCQMYTNVDQDMVGWPAPPGS
6gCON   APITAYAQQTRGLLGTIVTSLTGRDKNEAAGEIQILSTATQTFLATCVNGVCWTVYHGAGSKTLAGPRGPVCQMYTNVDQDMVGWPAPAGT
6hCON   APITAYAQQTRGIVGTIVTSLTGRDKNEVEGEIQVVSTATQSFLATAVNGVLWTVYYGAGSKTLAGPKGPVCQMYTNVDQDLVGWPAPAGA
6iCON   APITAYAQQTRGLMGTIVTSLTGRDKNVVEGEIQVVSTATQSFLATTLNGVLWTVYHGAGSKTLAGPKGPICQMYTNVEQDLVGWPAPSGA
6kCON   APITAYAQQTRGLVGTIVTSLTGRDKNEVEGEIQVVSTATQSFLATTVNGVLWTVYHGAGSKTLAGPKGPICQMYTNVDQDLVGWPAPPGA
6lCON   APITAYAQQTRGVVGTIVTSLTGRDKNEVEGEIQVVSTATQSFLATAVNGVLWTVYHGAGSKTLAGPKGPVCQMYTNVDQDLVGWPAPPGA
6nCON   APITAYTQQTRGLLGTIVTSLTGRDKNEVEGEIQVVSTATQTFLATSVNGVLWTVYHGAGSKTLAGPKGPVCQMYTNVDQDLVGWPAPPGS
6oCON   APITAYAQQTRGLLGTIVTSLTGRDKNEVEGEVQVISTATQTFLASTINGVLWTVYHGAGSKTLAGPKGPVCQMYTNVDQDMVGWPAPAGA
6pCON   APITAYHQQTRGLLGTIVTSLTGRDKNEVEGEIQVVSTATQTFLATAVNGVLWTVYHGAGSKTLAGPKGPVCQMYTNVDQDMVGWPAPPGA
6rCON   APITAYCQKTRGLLGTIVTSLTGRDKNEVEGEVQVVSTATQTFLATTINGVLWTVFHGAGSKTLAGPKGPICQMYTNVDQDMVGWPAPPGS
6tCON   APITAYHQQTRGLLGTIVTSLTGRDKNEVEGEVQVLSTATQTFLASTINGVMWTVYHGAGSKTLAGPKGPVCQMYTNVDQDLVGWPAPPGA
6uCON   APITAYAQQTRGMLGTIVTSLTGRDKNEVEGEVQVVSTATQTFLATAINGVLWTVFHGAGSKTLAGPKGPVCQMYTNVDQDLVGWPAPPGC
6vCON   APITAYAQQTRGLLGTIVTSLTGRDRNEVEGEIQIVSTATQTFLATAVNGVLWTVYHGAGSKTLSGPKGPVCQMYTNVDQDLVGWPAPAGS
6wCON   APITAYVQQTRGLLGTIVTSLTGRDKNQVEGEVQVLSTATQTFLGTTINGVLWTVYHGAGSKTLAGPKKPICQMYTNVDQDLVGWPAPAGA
```

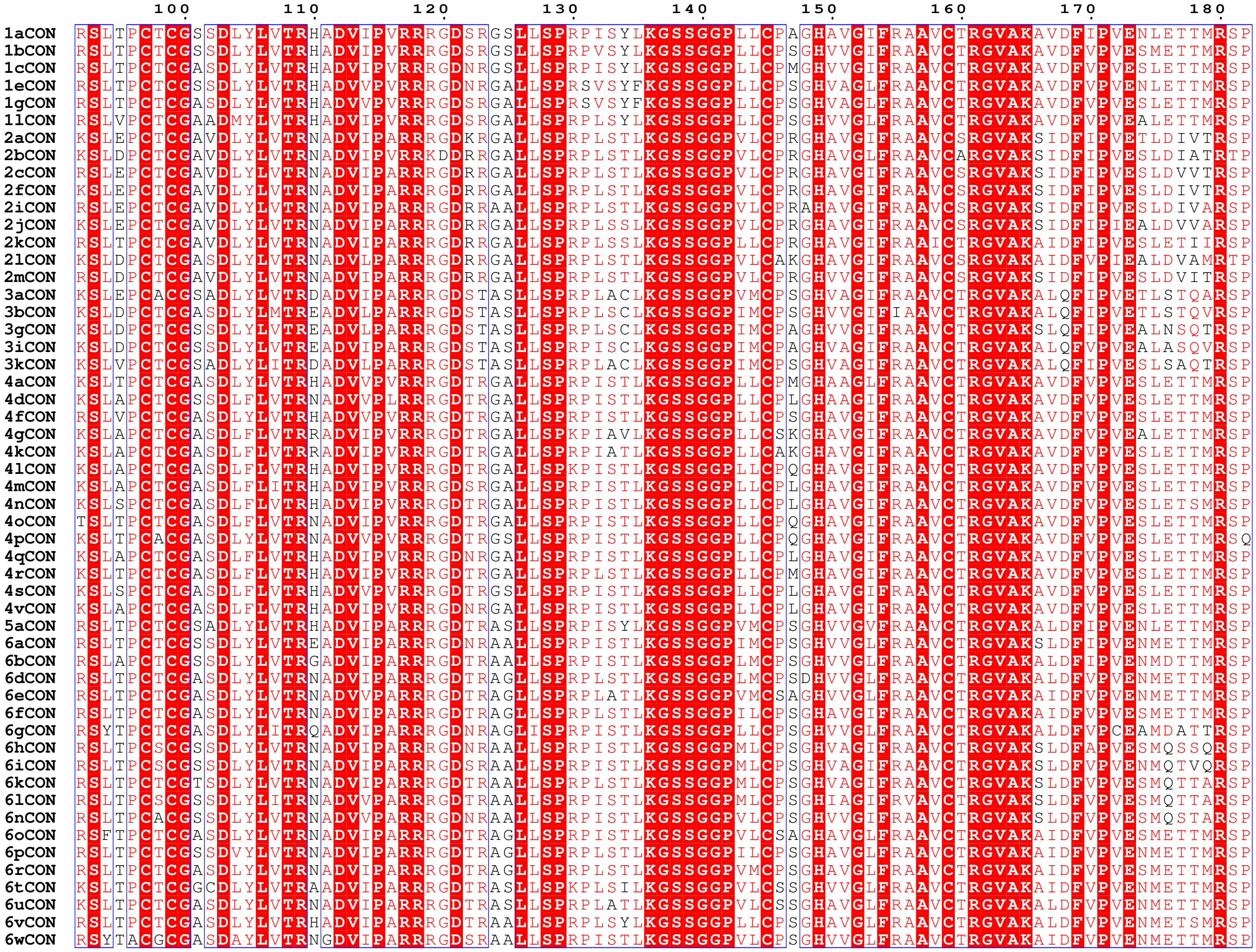
100
110
120
130
140
150
160
170
180
1aCON
1bCON
1cCON
1eCON
1gCON
1lCON
2aCON
2bCON
2cCON
2fCON
2iCON
2jCON
2kCON
2lCON
2mCON
3aCON
3bCON
3gCON
3iCON
3kCON
4aCON
4dCON
4fCON
4gCON
4kCON
4lCON
4mCON
4nCON
4oCON
4pCON
4qCON
4rCON
4sCON
4vCON
5aCON
6aCON
6bCON
6dCON
6eCON
6fCON
6gCON
6hCON
6iCON
6kCON
6lCON
6nCON
6oCON
6pCON
6rCON
6tCON
6uCON
6vCON
6wCON

```
            190       200       210       220       230       240       250       260       270
1aCON VFTDNSSPPAVPQSFQVAHLHAPTGSGKSTKVPAAYAAQGYKVLVLNPSVAATLGFGAYMSKAHGIDPNIRTGVRTITTGSPITYSTYGKF
1bCON VFTDNSSPPAVPQTFQVAHLHAPTGSGKSTKVPAAYAAQGYKVLVLNPSVAATLGFGAYMSKAHGVDPNIRTGVRTITTGAPITYSTYGKF
1cCON VFTDNSSPPTVPQSYQVAHLHAPTGSGKSTKVPAAYAAQGYKVLVLNPSVAATLGFGAYMSKAHGIDPNVRTGVRTITTGSPITYSTYGKF
1eCON VFTDNSTPPAVPQTYQVAHLHAPTGSGKSTKVPAAYAAQGYKVLVLNPSVAATLGFGAYMSKAHGIDPNIRTGVRTITTGSPITYSTYGKF
1gCON VFTDNSTPPAVPQTFQVAHLHAPTGSGKSTKVPAAYAAQGYKVLVLNPSVAATLGFGAYMSKAHGIDPNIRTGVRTITTGSPITYSTYGKF
1lCON VFTDNSTPPAVPQTFQVAHLHAPTGSGKSTKVPSAYAAQGYKVLVLNPSVAATLGFGAYMSKAYGIDPNIRTGVRTVTTGAAITYSTYGKF
2aCON TFSDNSTPPAVPQTYQVGYLHAPTGSGKSTKVPVAYAAQGYKVLVLNPSVAATLGFGAYLSKAHGINPNIRTGVRTVTTGEAITYSTYGKF
2bCON SFSDNSTPPAVPQTYQVGYLHAPTGSGKSTKVPAAYASQGYKVLVLNPSVAATLGFGAYMSKAHGINPNIRTGVRTVTTGDPITYSTYGKF
2cCON NFTDNSTPPAVPQTYQVGYLHAPTGSGKSTKVPAAYAAQGYKVLVLNPSVAATLGFGAYMSKAHGINPNIRTGVRTVTTGDAITYSTYGKF
2fCON SFSDNSTPPAVPQAYQVGYLHAPTGIGKSTKVPAAYAAQGYKVLVLNPSVAATLGFGAYMAKAYGINPNIRTGVRTVTTGESVTYSTYGKF
2iCON SFTDNGTPPAVPQTYQVGYLHAPTGSGKSTKVPAAYAAQGYKVLVLNPSVAATLGFGAYMSKAHGINPNIRTGVRTVTTGESITYSTYGKF
2jCON SFTDNSTPPAVPQTYQVGYLHAPTGSGKSTKVPAAYAAQGYKVLVLNPSVAATLGFGAYMSKAHGINPNIRTGVRTVTTGDSITYSTYGKF
2kCON VFTDNSSPPAVPQTYQVGYLHAPTGSGKSTKVPAAYAAQGYKVLVLNPSVAATLGFGAYMSKAHGINPNIRTGVRTITTGESITYSTYGKF
2lCON SFTDNGTPPAVPQTYQVGYLHAPTGSGKSTRVPAAYAAQGYKVLVLNPSVAATLGFGAYMSKAHGINPNIGTGVRTITTGDAITYSTYGKF
2mCON SFTDNSTPPAVPQTYQVGYLHAPTGSGKSTKVPAAYAAQGYKVLVLNPSVAATLGFGAYMSKAHGINPNVRTGVRTVTTGESITYSTYGKF
3aCON SFSDNSTPPAVPQSYQVGYLHAPTGSGKSTKVPAAYVAQGYNVLVLNPSVAATLGFGSFMSRAYGIDPNIRTGNRTVTTGAKLTYSTYGKF
3bCON SFSDNSTPPAVPESYQVGYLHAPTGSGKSTKVPAAYVAQGYSVLVLNPSVAATLGFGTYMSKAYGIDPNIRTGTRTITTGAKLTYSTYGKF
3gCON SFSDNSTPPAVPESYQVGYLHAPTGSGKSTKVPAAYVAQGYSVLVLNPSVAATQGFGMYMSKAYGIDPNIRTGNRTITTGAKLTYSTYGKF
3iCON SFSDNSVPPAVPEAYQVGYLHAPTGSGKSTKVPAAYVAQGYTVLVLNPSVAATLGFGMYMSKAYGIDPNIRTGTRTVTTGAKLTYSTYGKF
3kCON SFSDNSTPPAVPQTFQVGYLHAPTGSGESTKVPASYVAQGYTVLVLNPSVAATLGFGRFMSHAYGIDPNVRTGTRTITTGAKLTYSTYGKF
4aCON VFTDNSTPPAVPQTYQVAHLHAPTGSGKSTKVPAAYAAQGYKVLVLNPSVAATLGFGAYMSKAYGIDPNIRSGVRTITTGAPITYSTYGKF
4dCON VFTDNSTPPAVPQTYQVAHLHAPTGSGKSTKVPAAYAAQGYKVLVLNPSVAATLGFGAYMSKAHGIDPNIRSGVRTITTGAPITYSTYGKF
4fCON VFSDNSTPPAVPQTYQVAHLHAPTGSGKSTKVPAAYAAQGYKVLVLNPSVAATLGFGAYMSKAHGIDPNIRSGVRTISTGAPITYSTYGKF
4gCON VFTDNSTPPAVPQTYQVAHLHAPTGSGKSTKVPAAYAAQGYKVLVLNPSVAATLGFGNYMSKAYGIDPNIRSGVRTITTGAPITYSTYGKF
4kCON VFSDNSTPPAVPQTYQVAHLHAPTGSGKSTRVPAAYAAQGYKVLVLNPSVAATLGFGSYMSKAHGIDPNIRSGVRTITTGAPITYSTYGKF
4lCON VFTDNSTPPAVPQTYQVAHLHAPTGSGKSTKVPAAYAAQGYKVLVLNPSVAATLGFGAYMSKAYGIDPNIRSGVRTINTGASITYSTYGKF
4mCON VFTDNSTPPAVPQTYQVAHLHAPTGSGKSTKVPAAYAAQGYKVLVLNPSVAATLGFGAYMSKAYGIDPNIRSGVRTITTGAPLTYSTYGKF
4nCON VFSDNSTPPAVPQTYQVAHLHAPTGSGKSTKVPAAYAAQGYKVLVLNPSVAATLGFGAYMSKAYGIDPNIRSGVRTITTGAPITYSTYGKF
4oCON VFTDNSTPPAVPQTYQVAHLHAPTGSGKSTKVPAAYAAQGYKVLVLNPSVAATLGFGAYMSKAHGIDPNIRSGVRTITTGAPITYSTYGKF
4pCON VFTDNSTPPAVPQTYQVAHLHAPTGSGKSTRVPAAYAAQGYKVLVLNPSVAATLGFGAYMSKAHGIDPNIRSGVRTITTGAPITYSTYRKF
4qCON VFTDNSTPPAVPQTYQVAHLHAPTGSGKSTKVPAAYAAQGYKVLVLNPSVAATLGFGAYMSKAYGIDPNIRSGARTITTGAAITYSTYGKF
4rCON VFTDNSTPPAVPQTYQVAHLHAPTGSGKSTKVPAAYAAQGYKVLVLNPSVAATLGFGSYMSKAHGIDPNIRSGVRTITTGAPITYSTYGKF
4sCON VFTDNSTPPAVPQSYQVAHLHAPTGSGKSTKVPAAYAAQGYKVLVLNPSVAATLGFGAYMSKAYGIDPNIRSGVRTITTGAPITYSTYGKF
4vCON VFTDNSTPPAVPQSYQVAHLHAPTGSGKSTKVPAAYAAQGYKVLVLNPSVAATLGFGAYMSKAHGIDPNIRSGVRTITTGAPITYSTYGKF
5aCON VFTDNSTPPAVPHEFQVGHLHAPTGSGKSTKVPAAYAAQGYKVLVLNPSVAATLGFGAYMSKAYGVDPNIRTGVRTVTTGAAITYSTYGKF
6aCON SFTDNSTPPAVPQTYQVGYLHAPTGSGKSTRVPAAYASQGYKVLVLNPSVAATLSFGSYMRQAYGVEPNVRTGVRTVTTGGAITYSTYGKF
6bCON VFTDNSSPPAVPQTYQVGYLHAPTGSGKSTRVPAAYATQGYKVLVLNPSVAATLSFGSYMSKAHGIDPNIRTGVRTITTGGPVTYSTYGKF
6dCON VFTDNSTPPAVPQTYQVGYLHAPTGSGKSTKVPAAYASQGYKVLVLNPSVAATLGFGSYMSTAHGIDPNIRTGVRTITTGGPITYSTYGKF
6eCON VFSDNSSPPAVPQTYQVGYLHAPTGSGKSTKVPAAYASQGYKVLVLNPSVAATLGFGSYMSKAYGIDPNIRTGVRTVTTGGPITYSTYGKF
6fCON VFTDNSTPPAVPQTYQVGYLHAPTGSGKSTKVPAAYASQGYKVLVLNPSVAATLAFGQYMSKAHGIDPNIRTGVRTTTTGGAITYSTYGKF
6gCON TFTDNSTPPAVPQAYQVGYLHAPTGSGKSTKVPVAYASQGYKVLVLNPSVAATLSFGSYLSRAHGIDPNIRTGVRTITTGAPITYSTYGKF
6hCON SFSDNTSPPAVPQTYQVGYLHAPTGSGKSTKVPAAYAAQGYKVLVLNPSVAATLGFGSYMSTSHGIDPNIRTGVRTITTGGAITYSTYGKF
6iCON SFSDNSTPPAVPQTYQVGYLHAPTGSGKSTKVPAAYAAQGYKVLVLNPSVAATLGFGAYMSKAHGIDPNIRTGVRTITTGGPITYSTYGKF
6kCON SFSDNTTPPAVPQTYQVGYLHAPTGSGKSTKVPAAYAAQGYKVLVLNPSVAATLGFGAYMSTAHGIDPNIRTGVRTITTGGAITYSTYGKF
6lCON SFSDNSTPPAVPQTYQVGYLHAPTGSGKSTKVPAAYAAQGYKVLVLNPSVAATLGFGAYMSKAHGIDPNIRTGVRTITTGGAITYSTYGKF
6nCON SFSDNTSPPAVPQAYQVGYLHAPTGSGKSTKVPAAYAAQGYKVLVLNPSVAATLGFGAYMSQAHGIDPNIRTGVRTITTGGAITYSTYGKF
6oCON VFTDNSTPPAVPQTYQVGYLHAPTGSGKSTKVPAAYASQGYKVLVLNPSVAATLAFGAYMSKAYGVDPNIRTGVRTITTGGPITYSTYGKF
6pCON VFTDNSSPPAVPSTYQVGYLHAPTGSGKSTKVPAAYASQGYKVLVLNPSVAATLAFGSYMSKAHGVDPNIRTGVRTITTGGPITYSTYGKF
6rCON VFTDNSTPPAVPQSYQVGYLHAPTGSGKSTKVPAAYASQGYKVLVLNPSVAATLAFGQYMSKAYGIDPNIRTGVRTITTGGAITYSTYGKF
6tCON AFTDNSSPPAVPQTYQVGYLHAPTGSGKSTKVPAAYAAQGYKVLVLNPSVAATLGFGAYMSKAHGIDPNIRTGVRTITTGGAITYSTYGKF
6uCON VFTDNSSPPAVPSTYQVGYLHAPTGSGKSTKVPAAYAAQGYKVLVLNPSVAATLGFGAYMSKAHGIEPNIRTGVRTITTGGAITYSTYGKF
6vCON TFTDNSTPPAVPQTYQVGYLHAPTGSGKSTKVPASYASQGYKVLVLNPSVAATLGFGAYMRTAYGIEPNIRTGVRTLTTGGPVTYSTYGKF
6wCON TFSDNSTPPAVPQTYQVGYLHAPTGSGKSTKVPAAYASQGYKVLVLNPSVAATLSFGSFMSRAYGIDPNIRTGVRTITTGGPITYSTYGKF
```

```
               280       290       300       310       320       330       340       350       360
1aCON  LADGGCSGGAYDIIICDECHSTDATSILGIGTVLDQAETAGARLVVLATATPPGSVTVPHPNIEEVALSTTGEIPFYGKAIPLEVIKGGRH
1bCON  LADGGCSGGAYDIIICDECHSTDSTSILGIGTVLDQAETAGARLVVLATATPPGSVTVPHPNIEEVALSNTGEIPFYGKAIPIETIKGGRH
1cCON  LADGGCSGGAYDIIICDECHSVDATSILGIGTVLDQAETAGVRLTVLATATPPGSVTVPHSNIEEVALSAEGEIPFYGKAIPLNYIKGGRH
1eCON  LADGGCSGGAYDIIICDECHSTDATSILGIGTVLDQAETAGARLVVLATATPPGSVTVPHPNIEEVGLTNDGEIPFYGKAIPLAVVKGGRH
1gCON  LADGGCSGGAYDIIICDECHSTDSTSILGIGTVLDQAETAGARLVVLATATPPGSVTVPHPNIEEVALTNEGEIPFYGKAIPLAAIKGGRH
1lCON  LADGGCGAGAYDIIICDECHSTDSTTILGIGTVLDQAETAGARLVVLATATPPGSITVPHPNIEEVALSTTGEIPFYGKAIPLEVIKGGRH
2aCON  LADGGCAGGAYDIIICDECHAVDATTILGIGTVLDQAETAGVRLTVLATATPPGSVTTPHPNIEEVALGQEGEIPFYGRAIPLSYIKGGRH
2bCON  LADGGCSAGAYDVIICDECHSVDATTILGIGTVLDQAETAGARLVVLATATPPGTVTTPHSNIEEVALGHEGEIPFYGKAIPLAFIKGGRH
2cCON  LADGGCSGGAYDIIICDECHSVDSTTILGIGTVLDQAETAGVRLVVLATATPPGSVTTPHPNIEEVALGHEGEIPFYGKAIPLSAIKGGRH
2fCON  LADGGCSGGAYDIIICDECHSVDSTTILGIGTVLDQAETAGVRLVVLATATPPGSVTTPHPNIEEIALGHEGEIPFYGKAIPLANIKGGRH
2iCON  LADGGCSGGAYDIIICDECHSVDSTTILGIGTVLDQAETAGVRLTVLATATPPGSVTTPHPNIEEVALGHEGEIPFYGKAIPLANIKGGRH
2jCON  LADGGCSGGAYDIIICDECHSVDSTTILGIGTVLDQAETAGARLVVLATATPPGSVTTPHPNIEEVALGHEGEIPFYGKAIPLANIKGGRH
2kCON  LADGGCSGGAYDIIICDECHSTDSTTILGIGTVLDQAETAGARLVVLATATPPGSVTVPHPNIEEVALGNEGEIPFYGKAIPLEQIKGGRH
2lCON  LADGGCSGGAYDIIICDECHSIDATTILGIGTVLDQAETAGVRLVVLATATPPGSVTTPHPNIEEVALGHEGEIPFYGKAIPLSQLKGGRH
2mCON  LADGGCAGGAYDIIICDECHSVDATTILGIGTVLDQAETAGVRLVVLATATPPGSVTTPHPNIEEVSLGHEGEIPFYGKAIPLAHIRGGRH
3aCON  LADGGCSGGAYDVIICDECHAQDATSILGIGTVLDQAETAGVRLTVLATATPPGSITVPHSNIEEVALGSEGEIPFYGKAIPIALLKGGRH
3bCON  LADGGCSGGAYDVIICDECHAQDATSILGIGTVLDQAETAGVRLTVLATATPPGSITVPHPNIEEVALTSDGEIPFYGKALPLAMIKGGRH
3gCON  LADGGCSGGAYDVIICDECHAQDATSILGIGTVLDQAETAGVRLTVLATATPPGSVTVPHSNIEEVALTNDGEIPFYGKAIPLAVLKGGRH
3iCON  LADGGCSGGAYDVIICDECHAQDATSILGIGTVLDQAETAGVRLTVLATATPPGSVTVPHSNIEEVALTNDGEIPFYGKAIPIAQLKGGRH
3kCON  LADGGCSGGAYDVIICDECHAQDATSILGIGTVLDQAETAGARLVVLATATPPGSITVPHSNIEEVALTGEGEIPFYGRAIPLGVIKGGRH
4aCON  LADGGCSGGAYDIIICDECHSTDSTTILGIGTVLDQAETAGVRLVVLATATPPGSVTTPHSNIEEVALPTTGEVPFYGKAIPLELIKGGRH
4dCON  LADGGCAGGAYDIIICDECHSTDATTVLGIGTVLDQAETAGARLVVLATATPPGSVTTPHSNIEEVALPTTGEIPFYGKAIPLSLVKGGRH
4fCON  LADGGCSGGAYDIIICDECHSTDSTTILGIGTVLDQAETAGVRLVVLATATPPGSVTTPHPNIEEVALPTTGEIPFYGRAIPLAVIKGGRH
4gCON  LADGGCGGGAYDIIICDECHSTDATTILGIGTVLDQAETAGARLVVLATATPPGSVTTPHANIEEIALPTTGEVPFYGRAIPLEVLKGGRH
4kCON  LADGGCGGGAYDIIICDECHSTDSTTILGIGTVLDQAETAGVRLTVLATATPPGSVTTPHANIEEVALPTTGEIPFYGRAIPLELIKGGRH
4lCON  LADGGCGAGAYDIIICDECHSTDATTILGIGTVLDQAETAGARLVVLATATPPGSVTTPHPNIEEVALPTTGEIPFYGRAIPLDLVKGGRH
4mCON  LADGGCGGGAYDIIICDECHSTDATTILGIGTVLDQAETAGARLVVLATATPPGSITTPHANIEEVALPTTGEIPFYGRAIPLAVIKGGRH
4nCON  LADGGCGGGAYDIIICDECHSTDSTTILGIGTVLDQAETAGARLVVLATATPPGSVTTPHSNIEEVALPTTGEIPFYGKAIPLDVIKGGRH
4oCON  LADGGCGGGAYDIIICDECHSTDSTTILGIGTVLDQAETAGARLVVLATATPPGSITTPHSNIEEVALPTTGEIPFYGKAIPLACIKGGRH
4pCON  LADGGCGGGAYDIIICDECHSTDSTTILGIGTVLDQAETAGVRLVVLATATPPGSITTPHPNIEEVALPTTGEIPFYGKAIPLTLIKGGRH
4qCON  LADGGCGGGAYDIIICDECHSTDSTTILGIGTVLDQAETAGARLVVLATATPPGSVTTPHANIEEVALPTTGEIPFYGRAIPLALIKGGRH
4rCON  LADGGCGGGAYDIIICDECHSTDATTVLGIGTVLDQAETAGARLVVLATATPPGSVTTPHANIEEVALPTTGEIPFYGRAIPLEVIKGGRH
4sCON  LADGGCSGGAYDIIICDECHSTDATTILGIGTVLDQAETAGARLVVLATATPPGSVTTPHSNIEEIALPTTGEVPFYGRAIPLDYIKGGRH
4vCON  LADGGCGGGAYDIIICDECHSTDATTILGIGTVLDQAETAGARLVVLATATPPGSVTTPHANIEEVALPTTGEIPFYGRAIPLALVKGGRH
5aCON  LADGGCSGGAYDVIICDECHSQDATTILGIGTVLDQAETAGARLVVLATATPPGSVTTPHPNIEEVALPSEGEIPFYGRAIPLALIKGGRH
6aCON  LADGGCSGGAYDIIICDECHSTDPTTVLGIGTVLDQAETAGVRLTVLATATPPGSVTVPHPNITETALPTTGEIPFYGKAIPLEYIKGGRH
6bCON  LADGGCSGGAYDIIICDECHSTDPTTVLGIGTVLDQAETAGVRLTVLATATPPGSVTVPHPNITETALPTTGEIPFYGKCIPLEFIKGGRH
6dCON  LADGGCSGGAYDIIICDECHSTDPTTVLGIGTVLDQAETAGVRLTVLATATPPGSVTVPHPNITETALPSTGEVPFYGKAIPLECIKGGRH
6eCON  LADGGCSGGAYDIIICDECHSTDATTVLGIGTVLDQAETAGVRLTVLATATPPGSVTVPHPNITEVALPTTGEIPFYGKGIPLEYIKGGRH
6fCON  LADGGCSGGAYDIIICDECHSTDATTVLGIGTVLDQAETAGVRLTVLATATPPGSVTVPHPNITETALPLTGEVPFYGKAIPLEYIKGGRH
6gCON  LADGGCSGGAYDVIICDECHSTDPTTVLGIGTVLDQAETAGCRLTVLATATPPGSVTVPHPNIQETALPLTGEVPFYGKAIPLEYIKGGRH
6hCON  LADGGCSGGAYDVIICDECHSTDPTTVSGIGTVLDQAETSGVRLTVLATATPPGSVTVPHPNITESALPTTGEIPFYGKAVPLEYIKGGRH
6iCON  LADGGCSGGAYDIIICDECHSTDPTTILGIGTVLDQAETSGARLTVLATATPPGSVTVPHPNITEEALPTTGEIPFYGKGIPLDFIKGGRH
6kCON  LADGGCSGGAYDIIICDECHSTDPTTVLGIGTVLDQAESAGVRLTVLATATPPGSVTVPHPNITETALPSTGEVPFYGKAIPLEYIRGGRH
6lCON  LADGGCSGGAYDVIICDECHSTDPTTILGIGTVLDQAETAGARLTVLATATPPGSVTVPHPNITESALPTTGEIPFYGKAIPLELIKGGRH
6nCON  LADGGCSGGAYDVIICDECHSTDPTTVLGIGTVLDQAETAGVRLTVLATATPPGSITVPHPNITESALPTTGEIPFYGKAIPLEFIKGGRH
6oCON  LADGGCSGGAYDIIICDECHSTDPTTVLGIGTVLDQAETAGVRLTVLATATPPGSVTVPHPNISEVALPTTGEIPFYGRAIPLEYIKGGRH
6pCON  LADGGCSGGAYDIIICDECHSTDPTTILGIGTVLDQAETAGVRLTVLATATPPGSVTVPHPNISEVALPTTGEVPFYGRGIPLEYIKGGRH
6rCON  LADGGCSGGAYDIIICDECHSTDATTVLGIGTVLDQAETAGVRLTVLATATPPGSVTVPHPNITETALPLTGEVPFYGKGIPMEYIKGGRH
6tCON  LADGGCSGGAYDIIICDECHSTDPTTVLGIGTVLDQAETAGVRLTVLATATPPGSVTVPHPNITETALSTTGEIPFYGKAIPLEYIKGGRH
6uCON  LADGGCSGGAYDIIICDECHSTDPTTVLGIGTVLDQAETAGVRLTVLATATPPGSVTVPHPNITETALPTTGEVPFYGRAIPLDFIKGGRH
6vCON  LADGGCSGGAYDIIICDECHSTDPTTVLGIGTVLDQAETSGARLTVLATATPPGSVTVPHPNITETALPTTGEVPFYGKAIPLEYIKGGRP
6wCON  LADGGCSGGAYDIIICDECHSTDPTTVLGIGTVLDQAETAGVRLTVLATATPPGSVTVPHPNIQEIALPTTGEVPFYGKAIPLEYIKGGRH
```

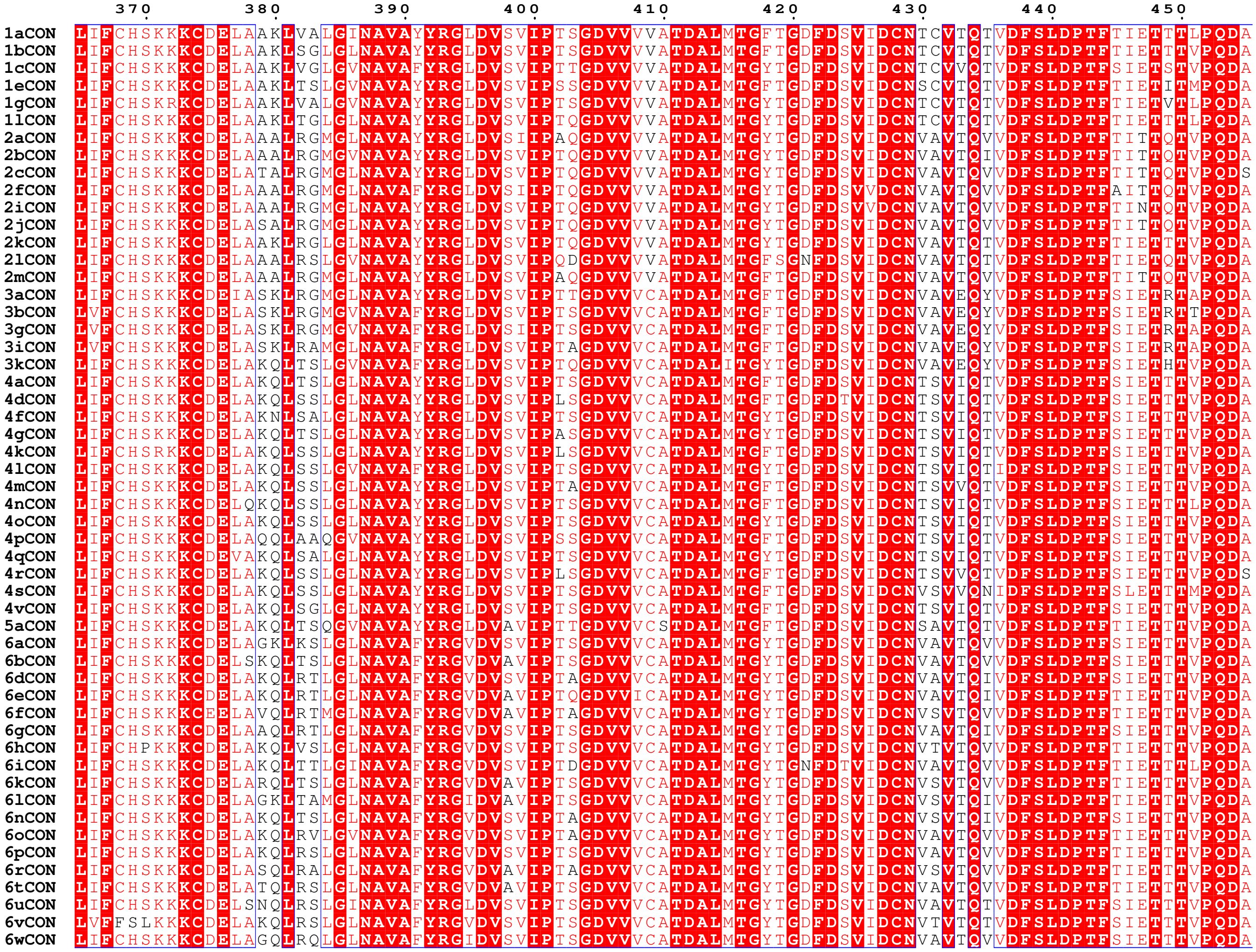

370 380 390 400 410 420 430 440 450
1aCON LIFCHSKKKCDELAAKLVALGLGINAVAYYRGLDVSVIPTSGDVVVVATDALMTGFTGDFDSVIDCNTCVTQTVDFSLDPTFTIETTTLPQDA
1bCON LIFCHSKKKCDELAAKLSGLGLNAVAYYRGLDVSVIPTSGDVVVVATDALMTGFTGDFDSVIDCNTCVTQTVDFSLDPTFTIETTTVPQDA
1cCON LIFCHSKKKCDELAAKLVGLGVNAVAFYRGLDVSVIPTTGDVVVVATDALMTGYTGDFDSVIDCNTCVVQTVDFSLDPTFSIETSTVPQDA
1eCON LIFCHSKKKCDELAAKLTSLGVNAVAYYRGLDVSVIPSSGDVVVVATDALMTGFTGDFDSVIDCNSCVTQTVDFSLDPTFTIETITMPQDA
1gCON LIFCHSKRKCDELAAKLVALGVNAVAYYRGLDVSVIPTSGDVVVVATDALMTGYTGDFDSVIDCNTCVTQTVDFSLDPTFTIETVTLPQDA
1lCON LIFCHSKKKCDELAAKLTGLGLNAVAYYRGLDVSVIPTQGDVVVVATDALMTGYTGDFDSVIDCNTCVTQTVDFSLDPTFTIETTTLPQDA
2aCON LIFCHSKKKCDELAAALRGMGLNAVAYYRGLDVSIIPAQGDVVVVATDALMTGYTGDFDSVIDCNVAVTQVVDFSLDPTFTITTQTVPQDA
2bCON LIFCHSKKKCDELAAALRGMGVNAVAYYRGLDVSVIPTQGDVVVVATDALMTGYTGDFDSVIDCNVAVTQIVDFSLDPTFTITTQTVPQDA
2cCON LIFCHSKKKCDELATALRGMGLNAVAYYRGLDVSVIPTQGDVVVVATDALMTGYTGDFDSVIDCNVAVTQVVDFSLDPTFTITTQTVPQDS
2fCON LIFCHSKKKCDELAAALRGMGLNAVAFYRGLDVSIIPTQGDVVVVATDALMTGYTGDFDSVVDCNVAVTQVVDFSLDPTFAITTQTVPQDA
2iCON LIFCHSKKKCDELAAALRGMGLNAVAYYRGLDVSVIPTQGDVVVVATDALMTGYTGDFDSVVDCNVAVTQVVDFSLDPTFTINTQTVPQDA
2jCON LIFCHSKKKCDELASALRGMGLNAVAYYRGLDVSVIPTQGDVVVVATDALMTGYTGDFDSVIDCNVAVTQVVDFSLDPTFTITTQTVPQDA
2kCON LIFCHSKKKCDELAAKLRGLGLNAVAYYRGLDVSVIPTQGDVVVVATDALMTGYTGDFDSVIDCNVAVTQTVDFSLDPTFTIETTTVPQDA
2lCON LIFCHSKKKCDELAAALRSLGVNAVAYYRGLDVSVIPQDGDVVVVATDALMTGFSGNFDSVIDCNVAVTQTVDFSLDPTFTIETQTVPQDA
2mCON LIFCHSKKKCDELAAALRGMGLNAVAYYRGLDVSVIPAQGDVVVVATDALMTGFTGDFDSVIDCNVAVTQVVDFSLDPTFTITTQTVPQDA
3aCON LIFCHSKKKCDEIASKLRGMGLNAVAYYRGLDVSVIPTTGDVVVCATDALMTGFTGDFDSVIDCNVAVEQYVDFSLDPTFSIETRTAPQDA
3bCON LVFCHSKKKCDELASKLRGMGVNAVAFYRGLDVSVIPTSGDVVVCATDALMTGFTGDFDSVIDCNVAVEQYVDFSLDPTFSIETRTTPQDA
3gCON LVFCHSKKKCDELASKLRGMGVNAVAFYRGLDVSIIPTSGDVVVCATDALMTGFTGDFDSVIDCNVAVEQYVDFSLDPTFSIETRTAPQDA
3iCON LVFCHSKKKCDELASKLRAMGLNAVAFYRGLDVSVIPTAGDVVVCATDALMTGFTGDFDSVIDCNVAVEQYVDFSLDPTFSIETRTAPQDA
3kCON LIFCHSKKKCDELAKQLTSLGVNAVAFYRGLDVSVIPTQGDVVVCATDALITGYTGDFDSVIDCNVAVEQYVDFSLDPTFSIETHTVPQDA
4aCON LIFCHSKKKCDELAKQLTSLGLNAVAYYRGLDVSVIPTSGDVVVCATDALMTGFTGDFDSVIDCNTSVIQTVDFSLDPTFSIETTTVPQDA
4dCON LIFCHSKKKCDELAKQLSSLGLNAVAYYRGLDVSVIPLSGDVVVCATDALMTGFTGDFDTVIDCNTSVIQTVDFSLDPTFSIETTTVPQDA
4fCON LIFCHSKKKCDELAKNLSALGLNAVAYYRGLDVSVIPTSGDVVVCATDALMTGYTGDFDSVIDCNTSVIQTVDFSLDPTFSIETTTVPQDA
4gCON LIFCHSKKKCDELAKQLTSLGLNAVAYYRGLDVSVIPASGDVVVCATDALMTGYTGDFDSVIDCNTSVIQTVDFSLDPTFSIETTTVPQDA
4kCON LIFCHSRKKCDELAKQLSSLGLNAVAYYRGLDVSVIPLSGDVVVCATDALMTGFTGDFDSVIDCNTSVIQTVDFSLDPTFSIETTTVPQDA
4lCON LIFCHSKKKCDELAKQLSSLGVNAVAFYRGLDVSVIPTSGDVVVCATDALMTGYTGDFDSVIDCNTSVIQTIDFSLDPTFSIETTTVPQDA
4mCON LIFCHSKKKCDELAKQLSSLGLNAVAYYRGLDVSVIPTAGDVVVCATDALMTGFTGDFDSVIDCNTSVVQTVDFSLDPTFSIETTTVPQDA
4nCON LIFCHSKKKCDELQKQLSSLGLNAVAYYRGLDVSVIPTSGDVVVCATDALMTGFTGDFDSVIDCNTSVIQTVDFSLDPTFSIETTTLPQDA
4oCON LIFCHSKKKCDELAKQLSSLGLNAVAYYRGLDVSVIPTSGDVVVCATDALMTGFTGDFDSVIDCNTSVIQTVDFSLDPTFSIETTTVPQDA
4pCON LIFCHSKKKCDELAQQLAAQGVNAVAYYRGLDVSVIPSSGDVVVCATDALMTGFTGDFDSVIDCNTSVIQTVDFSLDPTFSIETTTVPQDA
4qCON LIFCHSKKKCDEVAKQLSALGLNAVAYYRGLDVSVIPTSGDVVVCATDALMTGYTGDFDSVIDCNTSVIQTVDFSLDPTFSIETTTVPQDA
4rCON LIFCHSKKKCDELAKQLSSLGLNAVAYYRGLDVSVIPLSGDVVVCATDALMTGFTGDFDSVIDCNTSVVQTVDFSLDPTFSIETTTVPQDS
4sCON LIFCHSKKKCDELAKQLSSLGLNAVAYYRGLDVSVIPTSGDVVVCATDALMTGFTGDFDSVIDCNVSVVQNIDFSLDPTFSLETTTMPQDA
4vCON LIFCHSKKKCDELAKQLSGLGLNAVAYYRGLDVSVIPTSGDVVVCATDALMTGFTGDFDSVIDCNTSVIQTVDFSLDPTFSIETTTVPQDA
5aCON LIFCHSKKKCDELAKQLTSQGVNAVAYYRGLDVAVIPTTGDVVVCSTDALMTGFTGDFDSVIDCNSAVTQTVDFSLDPTFTIETTTVPQDA
6aCON LIFCHSKKKCDELAGKLKSLGLNAVAFYRGVDVSVIPTSGDVVVCATDALMTGYTGDFDSVIDCNVAVTQVVDFSLDPTFSIETTTVPQDA
6bCON LIFCHSKKKCDELSKQLTSLGLNAVAFYRGVDVAVIPTSGDVVVCATDALMTGYTGDFDSVIDCNVAVTQVVDFSLDPTFSIETTTVPQDA
6dCON LIFCHSKKKCDELAKQLRTLGLNAVAFYRGVDVSVIPTAGDVVVCATDALMTGYTGDFDSVIDCNVAVTQIVDFSLDPTFSIETTTVPQDA
6eCON LIFCHSKKKCDELAKQLRTLGLNAVAFYRGVDVAVIPTQGDVVICATDALMTGYTGDFDSVIDCNVAVTQIVDFSLDPTFTIETTTVPQDA
6fCON LIFCHSKKKCEELAVQLRTMGLNAVAFYRGVDVAVIPTAGDVVVCATDALMTGYTGDFDSVIDCNVSVTQVVDFSLDPTFTIETTTVPQDA
6gCON LIFCHSKKKCDELAAQLRTLGLNAVAFYRGVDVSVIPTSGDVVVCATDALMTGYTGDFDSVIDCNVAVTQIVDFSLDPTFSIETTTVPQDA
6hCON LIFCHPKKKCDELAKQLVSLGLNAVAFYRGVDVSVIPTSGDVVVCATDALMTGYTGDFDSVIDCNVTVTQVVDFSLDPTFTIETTTVPQDA
6iCON LIFCHSKKKCDELAKQLTTLGINAVAFYRGVDVSVIPTDGDVVVCATDALMTGYTGNFDTVIDCNVAVTQVVDFSLDPTFTIETTTLPQDA
6kCON LIFCHSKKKCDELARQLTSLGLNAVAFYRGVDVAVIPTSGDVVVCATDALMTGYTGDFDSVIDCNVSVTQVVDFSLDPTFTIETTTVPQDA
6lCON LIFCHSKKKCDELAGKLTAMGLNAVAFYRGIDVAVIPTSGDVVVCATDALMTGYTGDFDSVIDCNVSVTQIVDFSLDPTFTIETTTVPQDA
6nCON LIFCHSKKKCDELAKQLTSLGLNAVAFYRGVDVSVIPTAGDVVVCATDALMTGYTGDFDSVIDCNVSVTQIVDFSLDPTFSIETTTVPQDA
6oCON LIFCHSKKKCDELAKQLRVLGVNAVAFYRGVDVSVIPTSGDVVVCATDALMTGYTGDFDSVIDCNVAVTQVVDFSLDPTFTIETTTVPQDA
6pCON LIFCHSKKKCDELAKQLRSLGLNAVAFYRGVDVSVIPTSGDVVVCATDALMTGYTGDFDSVIDCNVAVTQVVDFSLDPTFTIETTTVPQDA
6rCON LIFCHSKKKCDELASQLRALGLNAVAFYRGVDVAVIPTAGDVVVCATDALMTGYTGDFDSVIDCNVSVTQVVDFSLDPTFTIETTTVPQDA
6tCON LIFCHSKKKCDELATQLRSLGLNAVAFYRGVDVAVIPTSGDVVVCATDALMTGYTGDFDSVIDCNVAVTQVVDFSLDPTFTIETTTVPQDA
6uCON LIFCHSKKKCDELSNQLRSLGINAVAFYRGLDVSVIPTSGDVVVCATDALMTGYTGDFDSVIDCNVAVTQTVDFSLDPTFSIETTTVPQDA
6vCON LVFFSLKKKCDELAKQLRSLGLNAVAFYRGVDVSVIPTSGDVVVCATDALMTGYTGDFDSVIDCNVTVTQTVDFSLDPTFSIETTTVPQDA
6wCON LIFCHSKKKCDELAGQLRQLGLNAVAFYRGIDVSVIPTSGDVVVCATDALMTGYTGDFDSVIDCNVAVTQVVDFSLDPTFSIETTTVPQDA

```
           460       470       480       490       500       510       520       530       540
1aCON VSRTQRRGRTGRGKPGIYRFVAPGERPSGMFDSSVLCECYDAGCAWYELTPAETTVRLRAYMNTPGLPVCQDHLEFWEGVFTGLTHIDAHF
1bCON VSRSQRRGRTGRGRRGIYRFVTPGERPSGMFDSSVLCECYDAGCAWYELTPAETSVRLRAYLNTPGLPVCQDHLEFWESVFTGLTHIDAHF
1cCON VSRSQRRGRTGRGKHGIYRYVSPGERPSGMFDSVVLCECYDAGCAWYELTPAETTVRLRAYLNTPGLPVCQDHLEFWESVFTGLTHIDAHF
1eCON VSRSQRRGRTGRGRHGIYRFVSPGERPSGMFDSSVLCECYDAGCAWYELTPAETTVRLRAYLNTPGLPVCQDHLEFWEGVFTGLTHIDAHF
1gCON VSRAQRRGRTGRGRHGVYRFVSPGERPSGMFDSSVLCECYDAGCAWYELTPAETTVRLRAYLNTPGLPVCQDHLEFWEGVFTGLTHIDAHF
1lCON VSRTQRRGRTGRGRQGIYRFVAPGERPSGMFDSSVLCECYDAGCAWYELTPAETTVRLRAYLNTPGLPVCQDHLEFWEGVFTGLTHIDAHF
2aCON VSRSQRRGRTGRGRLGIYRYVSTGERASGMFDSVVLCECYDAGAAWYELTPAETTVRLRAYFNTPGLPVCQDHLEFWEAVFTGLTHIDAHF
2bCON VSRSQRRGRTGRGRLGIYRYVSSGERPSGMFDSVVLCECYDAGAAWYELTPAETTVRLRAYFNTPGLPVCQDHLEFWEAVFTGLTHIDAHF
2cCON VSRSQRRGRTGRGKLGIYRYVSSGERASGMFDSVVLCECYDAGAAWYELTPAETTVRLRAYFNTPGLPVCQDHLEFWEAVFTGLTHIDAHF
2fCON VSRSQRRGRTGRGRSGTYRYVASGERPSGMFDSVVLCECYDAGAAWYELTPAETTVRLRAYFNTPGLPVCQDHLEFWEAVFTGLTHIDAHF
2iCON VSRSQRRGRTGRGRHGIYRYVSSGERPSGMFDSVVLCECYDAGAAWYELTPAETTVRLRAYLNTPGLPVCQDHLEFWEAVFTGLTHIDAHF
2jCON VSRSQRRGRTGRGRLGIYRYVSSGERASGMFDSVVLCECYDAGLAWYELTPAETTVRLRAYFNTPGLPVCQDHLEFWEAVFTGLTHIDAHF
2kCON VSRSQRRGRTGRGRLGIYRYVTSGERASGMFDSVVLCECYDAGAAWYELTPAETTVRLRAYLNTPGLPVCQDHLEFWEAVFTGLTHIDAHF
2lCON VSRSQRRGRTGRGRLGIYRYVSQGERPSGMFDSVVLCECYDAGLSWYELTPGETTVRLRAYFNTPGLPVCQDHLEFWEAVFTGLTHIDAHF
2mCON VSRSQRRGRTGRGRLGIYRYVSSGERASGMFDTVVLCECYDAGAAWYELTPAETTVRLRAYFNTPGLPVCQDHLEFWEAVFTGLTHIDAHF
3aCON VSRSQRRGRTGRGRLGTYRYVTPGERPSGMFDSVVLCECYDAGCSWYDLQPAETTVRLRAYLSTPGLPVCQDHLDFWESVFTGLTHIDAHF
3bCON VSRSQRRGRTGRGRQGTYRFVTPGERPSGMFDSVVLCECYDAGCSWYDLQPAETTVRLRAYLSTPGLPVCQDHLDFWESVFTGLTHIDAHF
3gCON VSRSQRRGRTGRGRTGIYRFVTPGERPSGMFDSVVLCECYDAGCAWYELAPSETTVRLRAYLSTPGLPVCQDHLDFWESVFTGLTHIDAHF
3iCON VSRSQRRGRTGRGRQGIYRFVTPGERPSGMFDTVVLCECYDAGCAWYGLTPAETTVRLRAYLSTPGLPVCQDHLDFWEGVFTGLTHIDAHF
3kCON VSRSQRRGRTGRGKSGTYRYVSPGERPSGMFDSVVLCECYDAGCAWYELTPSETTVRLRAYLSTPGLPVCQDHLEFWEGVFTGLTHIDAHF
4aCON VSRSQRRGRTGRGRLGIYRYVTPGERPSGIFDTSVICECYDAGCAWYELTPAETTTRLRAYFNTPGLPVCQDHLEFWESVFTGLTQIDGHF
4dCON VSRSQRRGRTGRGRLGIYRYVTPGERPSGIFDTSVLCECYDAGCAWYELTPAETTIRLRAYFNTPGLPVCQDHLEFWESVFTGLTHIDGHF
4fCON VSRSQRRGRTGRGRLGIYRFVTPGERPSGMFDTSVLCECYDAGCAWYELTPAETTTRLRAYFNTPGLPVCQDHLEFWESVFTGLTNIDAHF
4gCON VSRSQRRGRTGRGRLGIYRYVTPGERPSGMFDTSVLCECYDAGCAWYELTPAETTARLRAYFNTPGLPVCQDHLEFWESVFTGLTHIDAHF
4kCON VSRSQRRGRTGRGRLGIYRYVTPGERPSGMFDTSVLCECYDAGCAWYELTPAETTTRLRAYFNTPGLPVCQDHLEFWESVFTGLTHIDAHF
4lCON VSRSQRRGRTGRGKLGTYRFVTPGERPSGIFDTSVLCECYDAGCAWYELTPAETTTRLRAYYNTPGLPVCQDHLEFWESVFTGLVNIDGHF
4mCON VSRSQRRGRTGRGKLGIYRFVTPGERPSGIFDTSVLCECYDAGCAWYELTPAETTLRLRAYFNTPGLPVCQDHLEFWESVFTGLTHIDGHF
4nCON VSRSQRRGRTGRGRLGIYRFVTPGERPSGIFDSSVLCECYDAGCAWYELTPAETTARLRAYFNTPGLPVCQDHLEFWESVFTGLTNIDGHF
4oCON VSRSQRRGRTGRGRLGIYRYVTPGERPSGIFDTSVLCECYDAGCAWYELTPAETTTRLRAYFNTPGLPVCQDHLEFWESVFTGLVHIDGHF
4pCON VSRSQRRGRTGRGRLGVYRFVTPGERPSGMFDTSVLCECYDAGCAWYELTPAETTTRLRAYFNTPGLPVCQDHLEFWESVFTGLTNIDAHF
4qCON VSRSQRRGRTGRGRLGVYRFVTPGERPSGIFDTSVLCECYDAGCAWYELTPAETTTRLRAYFNTPGLPVCQDHLEFWESVFTGLTNIDGHF
4rCON VSRSQRRGRTGRGRLGIYRYVTPGERPSGMFDTAVLCECYDTGCSWYELTPAETTARLRAYFNTPGLPVCQDHLEFWESVFTGLTHIDAHF
4sCON VSRSQRRGRTGRGKLGIYRFVTPGERPSGIFDTAVLCECYDAGCAWYELTPAETTTRLRAYFNTPGLPVCQDHLEFWESVFTGLVHIDGHF
4vCON VSRSQRRGRTGRGRLGTYRFVTPGERPSGIFDTSVLCECYDAGCAWYELTPAETTTRLRAYFNTPGLPVCQDHLEFWESVFTGLTNIDAHF
5aCON VSRSQRRGRTGRGRHGIYRYVSSGERPSGIFDSVVLCECYDAGCAWYDLTPAETTVRLRAYLNTPGLPVCQDHLEFWEGVFTGLTNIDAHM
6aCON VSRSQRRGRTGRGKPGVYRFVSQGERPSGMFDTVVLCEAYDTGCAWYELTPSETTVRLRAYMNTPGLPVCQDHLEFWEGVFTGLTHIDAHF
6bCON VSRSQRRGRTGRGKPGVYRFVSQGERPSGMFDSVVLCEAYDTGCAWYELTPAETTVRLRAYLNTPGLPVCQDHLEFWEGVFTGLTHIDAHF
6dCON VARSQRRGRTGRGKPGVYRYVSQGERPSGMFDTVVLCEAYDVGCAWYELTPSETTVRLRAYLNTPGLPVCQDHLEFWEGVFTGMTHIDAHF
6eCON VARSQRRGRTGRGKPGIYRYVSQGERPSGMFDSIVLCEAYDTGCAWYELTPSETTVRLRAYLNTPGLPVCQDHLEFWEGVFTGLTHIDAHF
6fCON VARSQRRGRTGRGKPGVYRYVSQGERPSGTFDTIILCEAYDTGCAWYELTPSETTVRLRAYLNTPGLPVCQDHLEFWEGVFTGLTHIDAHF
6gCON VARSQRRGRTGRGKPGVYRYVSQGERPSGMFDTVVLCEAYDTGAAWYELTPAETTVRLRAYLNTPGLPVCQDHLEFWEAVFTGLTHIDAHF
6hCON VSRSQRRGRTGRGKHGVYRYVSQGERPSGMFDSVILCEAYDTGCAWYELTPAETTVRLRAYLNTPGLPVCQDHLEFWEGVFTGLTHIDAHF
6iCON VSRSQRRGRTGRGKHGVYRYVSLGERPSGLFDSVVLCEAYDTGCAWYELTPAETTVRLRAYLNTPGLPVCQDHLEFWEGVFTGLTHIDAHF
6kCON VSRSQRRGRTGRGKHGEYRYVSQGERPSGIFDSVVLCEAYDTGCAWYELTPSETTVRLRAYLNTPGLPVCQDHLEFWEGVFTGLTHIDAHF
6lCON VSRSQRRGRTGRGKHGVYMYVSQGERPSGIFDTVVLCEAYDTGCAWYELTPAETTVRLRAYLNTPGLPVCQDHLEFWEGVFTGLTHIDAHF
6nCON VSRSQRRGRTGRGKHGEYRYVSQGERPSGMFDSVVLCEAYDTGCAWYELTPAETTVRLRAYLNTPGLPVCQDHLEFWEGVFTGLTHIDAHF
6oCON VARSQRRGRTGRGKPGVYRYVSQGERPSGTFDTVVLCEAYDTGCAWYELTPSETTVRLRAYLNTPGLPVCQDHLEFWEGVFTGLTHIDAHF
6pCON VARSQRRGRTGRGKPGVYRYVSQGERPSGTFDTVVLCEAYDVGCAWYELTPSETTVRLRAYLNTPGLPVCQDHLEFWEGVFTGLTHIDAHF
6rCON VARSQRRGRTGRGKPGVYRYVSQGERPSGTFDTIVLCEAYDTGCAWYELTPSETTVRLRAYLNTPGLPVCQDHLEFWEGVFTGLTHIDAHF
6tCON VVRSQRRGRTGRGKPGVYRYVSQGERPSGMFDSIVLCEAYDTGCAWYELTPSETTVRLRAYLNTPGLPVCQDHLEFWEGVFTGLTHIDAHF
6uCON VSRSQRRGRTGRGKPGIYRFVSKGERPSGMFDTVVLAEAYDTGLAWYELAPSETTVRLRAYLNTPGLPVCQDHLEFWEGVFTGLTHIDAHF
6vCON VSRSQRRGRTGRGKPGVYRFVAQGERPSGMFDTVVLCEAYDTGLSWYELTPSEITVRLRAYMNTPGLPVCQDHLEFWEGVFTGLTHIDAHF
6wCON VSRSQRRGRTGRGKPGVYRYVSQGERPSGTFDTVILCEAYDTGCSWYELTPSETTVRLRAYLNTPGLPVCQDHLEFWEAVFTGLTHIDAHF
```

```
             550       560       570       580       590       600       610       620       630
1aCON  LSQTKQSGENFPYLVAYQATVCARAQAPPPSWDQMWKCLIRLKPTLHGPTPLLYRLGAVQNEVTLTHPVTKYIMTCMSADLEVVT
1bCON  LSQTKQAGDNFPYLVAYQATVCARAQAPPPSWDQMWKCLIRLKPTLHGPTPLLYRLGAVQNEVTLTHPITKYIMACMSADLEVVT
1cCON  LSQTKQSGENFPYLVAYQATVCARAKAPPPSWDQMWKCLIRLKPTLTGATPLLYRLGGVQNEITLTHPITKYIMACMSADLEVVT
1eCON  LSQTKQGGENFPYLVAYQATVCARAQAPPPSWDQMWKCLIRLKPTLHGPTPLLYRLGAVQNEVTLTHPVTKYIMTCMAADLEVVT
1gCON  LSQTKQGGENFPYLVAYQATVCARAQAPPPSWDQMWKCLIRLKPTLHGPTPLLYRLGAVQNEVTLTHPITKYIMTCMSADLEVVT
1lCON  LSQTKQAGDNFPYLTAYQATVCAKALAPPPSWDQQWKCLIRLKPTLHGPTPLLYRLGAVQNDITLTHPITKYIMTCMSADLEVVT
2aCON  LSQTKQAGENFAYLVAYQATVCARAKAPPPSWDVMWKCLTRLKPTLVGPTPLLYRLGSVTNEVTLTHPVTKYIATCMQADLEVMT
2bCON  LSQTKQGGDNFAYLTAYQATVCARAKAPPPSWDVMWKCLTRLKPTLTGPTPLLYRLGAVTNEVTLTHPVTKYIATCMQADLEIMT
2cCON  LSQTKQAGENFPYLVAYQATVCARAKAPPPSWDVMWKCLIRLKPTLVGPTPLLYRLGSVTNEVTLTHPVTKYIATCMQADLEIMT
2fCON  LSQTKQAGENFPYLVAYQATVCARAKAPPPSWDTMWKCLIRLKPTLVGPTPLLYRLGAVSNEVTLTHPVTKYIATCMQADLEIMT
2iCON  LSQTKQAGENFPYLVAYQATVCARAKAPPPSWDTMWKCLIRLKPTLTGPTPLLYRLGAVSNEVTLTHPVTKYISTCMQADLEIMT
2jCON  LSQTKQSGENFPYLVAYQATVCARAKAPPPSWDTMWKCLIRLKPTLVGPTPLLYRLGAVSNEVTLTHPVTKYIATCMQADLEVMT
2kCON  LSQTKQAGENFPYLVAYQATVCARAKAPPPSWDVMWKCLIRLKPTLTGPTPLLYRLGAVTNEVTLTHPITKYIAACMQADLEVVT
2lCON  LSQTKQSGENFAYLVAYQATVCARARAPPPSWDIMWKCLTRLKPTLNGPTPLLYRLGAVSNEVTLTHPVTKYIATCMQADLEVLT
2mCON  LSQTKQAGDNFAYLVAYQATVCARAKAPPPSWDTMWKCLIRLKPTLNGPTPLLYRLGPVTNEVTLTHPVTKYIATCMQADLEIMT
3aCON  LSQTKQQGLNFSYLTAYQATVCARAQAPPPSWDETWKCLVRLKPTLHGPTPLLYRLGPVQNEICLTHPVTKYIMACMSADLEVTT
3bCON  LSQTKQQGLNFAYLVAYQATVCARAKASPPSWDEMWKCLIRLKPTLQGPTPLLYRLGAIQNDICTTHPITKYIMACMSADLEVTT
3gCON  LSQTKQQGLNFPYLVAYQATVCARAQAPPPSWDEMWKCLIRLKPTLHGQTPILYRLGPIQNEVCTTHPVTKYIMACMSADLEVTT
3iCON  LSQTKQQGLNFAYLVAYQATVCARAQASPPSWDEMWKCLIRLKPTLHGPTPLLYRLGPVQNDVCTSHPVTKYIMTCMSADLEVTT
3kCON  LSQTKQQGLNFPYLTAYQATVCARAAALPPSWDETWKCLIRLKPTLHGPTPLLYRLGAVQNEICTTHPVTKYIATCMAADLEVAT
4aCON  LSQTKQSGENFPYLVAYQATVCARALAPPPSWDTMWKCLIRLKPTLHGPTPLLYRLGSVQNEVTLTHPITKYIMACMSADLEVVT
4dCON  LSQTKQAGDNYPYLVAYQATVCAKALAPPPSWDTMWKCLLRLKPTLRGPTPLLYRLGSVQNEVVLTHPITKYIMACMSADLEVVT
4fCON  LSQTKQAGDNYPYLVAYQATVCARALAPPPSWDTMWKCLIRLKPTLHGPTPLLYRLGAVQNEVTLTHPITKYIMACMSADLEVVT
4gCON  LSQVKQAGENYPYLVAYQATVCARAQAPPPSWDVMWKCMIRLKPTLHGPTPLLYRLGSVQNEVTLTHPVTKYIMACMSADLEVVT
4kCON  LSQTKQSGENYPYLVAYQATVCARAQAPPPSWDAMWKCLIRLKPTLHGPTPLLYRLGSVQNEVTLTHPVTKYIMACMSADLEVVT
4lCON  LSQTKQSGENFPYLVAYQATVCARALAPPPSWDTMWKCLIRLKPTLRGPTPLLYRMGSVQNEVTLTHPITKYIMACMSADLEVVT
4mCON  LSQTKQQGENYPYLIAYQATVCAKALAPPPSWDTMWKCLIRLKPTLHGPTPLLYRLGGVQNEVTLTHPVTKYIMACMSADLEVVT
4nCON  LSQAKQAGENYPYLVAYQATVCARALAPPPSWDTMWKCLIRLKPTLHGPTPLLYRLGSVQNEVTLTHPITKYIMACMSADLEVVT
4oCON  LSQTKQAGENFPYLVAYQATVCAKALAPPPSWDTMWKCLIRLKPTLHGPTPLLYRLGAVQNEVTLTHPITKYIMACMSADLEVVT
4pCON  LSQTKQAGDNYPYLVAYQATVCARALAPPPSWDTMWKCLIRLKPTLHGPTPLLYRLGAVQNEVTLTHPITKYIMACMSADLEVVT
4qCON  LSQTKQAGENYPYLVAYQATVCARALAPPPSWDTTWKCLTRLKPTLHGPTPLLYRLGSVQNEVTLTHPITKYIMACMSADLEVVT
4rCON  LSQTKQAGDNFAYLVAYQATVCARAKAPPPSWDTMWKCLIRLKPTLQGATPLLYRLGAVQNEVTLTHPITKYIMACMSADLEVVT
4sCON  LSQTKQAGENYPYLVAYQATVCARAKAPPPSWDTMWKCLSRLKPTLQGPTPLLYRLGSVQNEVTLTHPITKYIMACMSADLEVVT
4vCON  LSQTKQAGDNYPYLVAYQATVCARALAPPPSWDTMWKCLIRLKPTLHGPTPLLYRLGSVQNEVTLTHPITKYIMACMSADLEVVT
5aCON  LSQTKQGGENFPYLVAYQATVCVRAKAPPPSWDTMWKCMLRLKPTLTGPTPLLYRLGAVQNEITLTHPITKYIMACMSADLEVIT
6aCON  LSQTKQGGENFAYLVAYQATVCARAKAPPPSWDTMWKCLIRLKPTLTGPTPLLYRLGAVQNEIITTHPITKYIMTCMSADLEVIT
6bCON  LSQTKQGGENFAYLVAYQATVCARAKAPPPSWDVMWKCLTRLKPTLTGPTPLLYRLGAVQNEIVTTHPITKYIMTCMSADLEVIT
6dCON  LSQTKQGGENFAYLVAYQATVCARAKAPPPSWDTMWKCLIRLKPMLTGPTPLLYRLGAVQNEIITTHPITKYIMTCMAADLEVIT
6eCON  LSQTKQGGENLAYLVAYQATVCARAKAPPPSWDNMWKCLIRLKPVLTGPTPLLYRLGAVQNEITTTHPITKYIMTCMSADLEVIT
6fCON  LSQCKQSGENLSYLVAYQATVCARAKAPPPSWDTMWKCLIRLKPMLTGPTPLLYRLGPVNNEITTTHPITKYIMTCMSADLEVIT
6gCON  LSQTKQGGENFAYLVAYQATVCARAKAPPPSWDTMWKCLLRLKPTLTGPTPLLYRLGAVQNEVTPTHPVTKYIMACMSADLEVIT
6hCON  LSQTKQAEENFAYLVAYQATVCARAKAPPPSWDTMWKCLIRLKPMLTGPTPLLYRLGPVQNEVVTTHPITKYIMTCMSADLEVIT
6iCON  LSQTKQAGESLAYLVAYQATVCARAKAPPPSWDVMWKCLLRLKPTLTGPTPLLYRLGAVQNEICLTHPITKYIMTCMSADLEVVT
6kCON  LSQTKQSGENFAYLVAYQATVCARAKAPPPSWDTMWKCLIRLKPMLTGPTPLLYRLGAVQNEITTTHPVTKYIMTCMSADLEVIT
6lCON  LSQTKQSGENFAYLVAYQATVCARAKAPPPSWDTMWKCLIRLKPMLTGPTPLLYRLGAVQNEITTTHPITKYIITCMSADLEVIT
6nCON  LSQTKQGGENFAYLVAYQATVCARAKAPPPSWDTMWKCLIRLKPMLTGPTPLLYRLGAVQNDIITTHPITKYIMTCMSADLEVIT
6oCON  LSQTKQGGENFAYLVAYQATVCARAKAPPPSWDNMWKCLIRLKPVLTGPTPLLYRLGAVQNEITTTHPITKYIMTCMSADLEVIT
6pCON  LSQTKQGGENFAYLVAYQATVCARAKAPPPSWDNMWKCLLRLKPMLVGPTPLLYRLGAVQNEVTTTHPITKYIMTCMSADLEVIT
6rCON  LSQCKQSGENLAYLVAYQATVCARAKAPPPSWDTMWKCLQRLKPMLTGPTPLLYRLGAVQNEIVTTHPITKYIMTCMSADLEVIT
6tCON  LSQTKQGGENFAYLVAYQATVCARAKAPPPSWDNMWKCLLRLKPVLTGPTPLLYRLGAVQNEITTTHPITKYIMTCMSADLEVIT
6uCON  LSQTKQAGENFSYLVAYQATVCARAKAPPPCWDVMWKCLSRLKPMLVGPTPLLYRLGPVQNDIITTHPITKYIMTCMSADLEVIT
6vCON  LSQTKQAGENFPYLVAYQATVCARAKAPPPSWDVMWKFLTRLKPMLTGPTPLLYRLGSVANETTTTHPITKYIMTCMAADLEVVT
6wCON  LSQTKQAGENFAYLVAYQATVCARAKAPPPSWDQMWKCLIRLKPTLTGPTPLLYRLGAVQNDITTTHPVTKYIMACMSADLEVIT
```

HCV NS5A

```
        1        10        20        30        40        50        60        70        80        90
1aCON   SGSWLRDIWDWICEVLSDFKTWLKAKLMPQLPGIPFVSCQRGYRGVWRGDGIMHTRCHCGAEITGHVKNGTMRIVGPKTCRNMWSGTFPI
1bCON   SGSWLRDVWDWICTVLTDFKTWLQSKLLPRLPGVPFFSCQRGYKGVWRGDGIMQTTCPCGAQITGHVKNGSMRIVGPKTCSNTWHGTFPI
1cCON   AGSWLKDVWDWICEVLSDFKSWLKAKLMPQLPGIPFVSCQRGYRGVWRGEGIMNTRCPCGADITGHVKNGSMRIVGPKTCSNTWRGSFPI
1eCON   SGSWLREIWDWVCTVLADFKVWLQSKLLPRMPGIPFLSCQRGYRGVWRGDGIMNTVCPCGAQISGHVKNGSMRIVGPRTCSNTWHGTFPI
1e2CON  SGSWLREIWEWVCTVLSDFKCWLKAKLMPQMPGIPFLSCQRGYRGVWRGDGVMHTVCSCGAQISGHVKNGSMRIVGPKTCSNTWHGTFPI
1gCON   SGSWLSEIWDWICTVLSDFKTWLRAKLLPQLPGVPFLSCQRGYRGVWRGDGVMNTTCPCGAQITGHVKNGTMKLVGPKTCSNTWHGSFPI
1lCON   SGSWLRDIWDWVCEVLGDFKRWLGAKLMPQMPGIPFFSCQRGYKGVWRGDGVMNTVCPCGAQIAGHVKNGTMRISGPKTCSNTWHGTFPV
2aCON   SGSWLRDVWDWVCTILTDFKNWLTSKLFPKMPGLPFISCQKGYKGVWAGTGIMTTRCPCGANISGNVRLGSMRITGPKTCMNTWQGTFPI
2bCON   SGSWLRDIWDWVCSILTDFKNWLSSKLLPKMPGLPFISCQKGYRGVWAGTGVMTTRCPCGANISGHVRMGTMKITGPKTCLNLWQGTFPI
2cCON   SGSWLRDVWDWVCSILTDFKNWLSAKLCPKLPGLPFISCQKGYRGVWAGTGIMTTRCPCGANITGNVRLGTMRLSGPKTCLNTWQGTFPI
2eCON   SGSWLRDVWDWICTILADFKNWLSAKLFPKMPGIPFISCQKGYRGVWAGTGIMTTRCSCGANISGHVRFGSMRITGPKTCMNTWQGTFPI
2iCON   AGSWLWDVWDWICSILTDFKNWLSSKLFPKMPGLPFVSCQKGYKGVWAGTGIMTTRCPCGANISGNVRLGSMRITGPKTCMNTWQGTFPI
2jCON   SGSWLRDIWDWICTILTDFKNWLSSKLLPKMPGIPFISCQKGYRGVWSGTGIMTTRCPCGANISGNVRLGSMRITGPKTCMNTWQGTFPI
2kCON   AGSWLRDIWDWICTILTDFKTWLQTKLLPKLPGLPFISCQRGYKGVWRGTGIMTTTCPCGAQITGNVKNGSMRIVGPKTCSNTWQGTFPI
2lCON   SESWLRDIWDWVCTVLSDFKTWLSAKLLPKLPGIPFLSCQKGYHGTWSGTGVMTTRCPCGALISGHVRLGTMRITGPRTCLNTWQGSFPI
2l2CON  SDSWLRDIWDWVCTVLSDFKTWLSSKLLPKLPGVPFLSCQKGYRGTWSGTGVMTTRCPCGAYISGHVRLGTMRITGPRTCLNTWQGSFPI
2mCON   AGSWLSDIWDWVCTILTDFKNWLSSKLLPKMPGIPFISCQKGYRGVWAGTGIMTTKCPCGAAISGNVRLGSMRITGPKTCMNTWQGAFPI
2qCON   AGSWLSDVWDWVCTILTDFKNWLSSKLLPKMPGLPFISCQKGYRGVWAGTGIMTTRCPCGANISGNVRLGTMRITGPKTCMNTWQGTFPI
3aCON   SGDWLRDIWDWVCTVLSDFKTWLSAKIMPALPGLPFISCQKGYKGVWRGDGVMSTRCPCGASITGHVKNGSMRLAGPRTCANMWYGTFPI
3bCON   NGDWLHDIWDWVCTVLSDFKTWLSAKIMPKMPGVPFFSCQRGYKGVWRGDGVMTTRCPCGKELTGHVKNGSMRIAGSGLCANMWYGTFPI
3b2CON  NGDWLHDIWDWVCTVLSDFKTWLSAKIMPKMPGVPFFSCQRGYKGVWRGDGVMTTRCPCGKDLTGHVKNGSMRIAGSGLCANMWYGTFPI
3gCON   SGDWLRDVWDWVCNVLSDFKTWLTAKIMPKMPGIPFISCQKGYKGVWRGDGVTTTRCPCGAILTGHVKNGSMRMVGPRLCANMWLGTFPI
3g2CON  SGDWLRDIWDWVCTVLSDFKTWLSAKIMPKVPGIPFISCQKGYRGVWRGDGVTTTRCPCGAILTGHVKNGSMRVVGPRLCANMWHGTFPI
3hCON   AGEWLCDVWSWICTVLSDFKTWLSAKLMPALPGIPFLSCQKGFKGIWRGDGVMTTRCPCGVTISGHVKNGSMRLTGPRRCANLWHGTFPI
3iCON   AGDWLYDIWNWICTVLSDFKTWLSSKIMPKLPGVPFLSCQKGYKGVWRGDGVMTTRCPCGKMLTGHVKNGSMRMVGSSLCANMWHGTFPI
3kCON   DGNWLYDIWNWVCTVLADFKLWLGAKILPKMPGIPFLSCQKGYRGTWRGDGVVSTRCPCGALLSGHVKNGTMRLVGPRWCANTWHGTFPI
4aCON   AESWLWEVWDWVCTVLSDFKTWLKAKLLPLMPGIPFLSCQRGYKGEWRGDGVMHTTCPCGAELAGHIKNGSMRITGPKTCSNTWHGTFPI
4bCON   DTSWLREIWDWVCTVLSDFKTWLKAKLLPAMPGLPFLSCQRGYKGEWRGDGVMHTKCPCGADIAGHIKNGSMRIVGPRTCSNTWHGTFPI
4b2CON  DASWLREIWDWVCTVLSDFKSWLKAKLMPSMPGIPFLSCQRGYRGVWRGDCVMHTKCPCGAEIAGHIKNGSMRIVGPKTCSNTWHGTFPI
4cCON   DASWLHDIWDWVCTVLSDFKTWLKAKLLPRMPGIPFLSCQRGYRGEWRGDGVMHTTCPCGAQLAGHIKNGSMRITGPKTCSNTWHGTFPI
4dCON   DRSWLWEIWDWVCTVLSDFKTWLKAKLLPRMPGVPFFSCQRGYRGVWRGDGVMHTTCTCGAELAGHVKNGSMRIVGPKTCSNTWHGTFPI
4fCON   DSSWLWEIWDWVCTVLSDFKTWLKAKLLPRMPGIPFLSCQKGYKGEWRGDGVMHTTCPCGAELAGHIKNGSMRIVGPKTCSNTWHGTFPI
4gCON   DSSWLREIWDWVCTVLSDFKTWLKAKLLPLMPGLPFLSCQKGFRGEWRGDGVMHTTCPCGASIAGHIKNGSMRIVGPKTCSNTWHGTFPI
4kCON   DSTWLREVWEWVCTVLTDFKTWLKAKLLPRLPGIPFLSCQKGFKGEWRGDGVMHTTCPCGAEIAGHVKNGSMRIVGPKTCSNTWHGTFPI
4lCON   DTSWLWEVWSWVCTVLSDFKTWLKAKLLPRMPGVPFLSCQRGYKGEWRGDGVMHTTCPCGAELAGHIKNGSMRITGPKTCSNTWHGTFPI
4l2CON  DTSWLWEVWSWVCTVLSDFKTWLKAKLLPRMPGVPFLSCQRGYKGAWRGDGVMHTTCPCGAVLAGHIKNGSMRITGPKTCSNTWHGTFPI
4mCON   DRSWLWEVWDWVCTVLSDFKTWLKAKLLPSMPGIPFFSCQKGYKGEWRGDGVVHTKCPCGARLAGHIKNGSMRIVGPKTCSNTWQGTFPI
4m2CON  DRSWLWEVWDWVCTVLSDFKTWLKAKLLPSMPGVPFLSCQKGYKGEWRGDGVVHTQCRCGARLAGHIKNGSMRIVGPKTCSNTWQGTFPI
4nCON   DTSWLREVWDWVCTVLSDFKTWLKAKLLPRMPGIPFLSCQKGYKGEWRGDGVMHTTCTCGAEIAGHIKNGSMRIVGPSTCSNTWHGTFPI
4oCON   TLDWFWELWDWVCTVLSDFKTWLKAKLMPTMPGIPFLSCQRGFKGEWRGDGVMHTICPCGANLAGHIKNGSMRIVGPKTCSNTWHGTFPI
4pCON   GNSWLWEVWDWVCTVLSDFKTWLKAKLLPRMPGLPFLSCQRGYKGEWRGDGVMHTTCPCGAELAGHIKNGSMRITGPKTCSNTWHGTFPI
4qCON   CTSWLRDVWDWVCTVLSDFKTWLKAKLLPRMPGIPFLSCQKGYKGEWRGDGVMHTTCPCGAELAGHIKNGSMRIVGPKTCSNTWHGTFPI
4rCON   DSSWLREVWEWVCTVLTDFKTWLKAKLMPRLPGIPFLSCQRGFRGEWRGDGVMHTVCPCGASLAGHVKNGSMRIVGPKTCSNVWHGTFPI
4sCON   DTSWLREIWEWVCTVLSDFKTWLKAKLLPRLPGIPFLSCQKGYKGEWRGDGLMHTMCPCGAELAGHVKNGTMRIAGPKTCSNTWHGTFPI
4vCON   ATSWLWDVWDWVCTVLSDFKTWLKAKLLPRMPGVPFLSCQRGYRGEWRGDGVMHTTCPCGAELAGHIKNGSMRITGPKTCSNTWHGTFPI
5aCON   DGTWLRAIWDWVCTALTDFKAWLQAKLLPQLPGVPFLSCQRGYKGVWRGDGVNSTKCPCGATISGHVKNGTMRIVGPKLCSNTWHGTFPI
6aCON   ATSWLRDVWDWVCTVLSDFKVWLQAKLLPRLPGIPFLSCQTGYRGVWAGDGVCHTTCTCGAVIAGHVKNGTMKITGPKTCSNTWHGTFPI
6bCON   ASSWLRDVWDWVCTVLSDFKTWLKAKITPRIPGIPFISCQAGYRGVWAGDGVCHTTCSCGAQIAGHVKNGSMKITGPRMCSNTWHGTFPI
6dCON   SCSWLKDVWDWVCTVLSDFKTWLRAKLVPTLPGIPFISCQRGFRGVWRGDGVNYTTCSCGANITGHVKNGSMKIVGPKMCSNVWNNRFPI
6eCON   ATSWLRDVWDWVCTVLSDFKTWLRAKLMPSLPGIPFLSCQRGFRGTWRGDGICCTSCPCGARITGHVRNGSMRITGPRTCSNVWHGTFPI
6e2CON  ATSWLRDVWDWVCTVLSDFKTWLRAKLMPSLPGIPFLSCQRGFRGTWRGDGICCTSCPCGARITGHVRNGSMRITGPRTCSNVWHGTFPI
6fCON   ATSWLRDIWDWVCTVLSDFKTWLKAKITPSLPGIPFLSCQRGYRGTWRGDGICHTTCPCGATITGHVKNGTMKIVGPRTCSNTWHGTFPI
6gCON   GSTWLRDIWDWVCTVLSDFRVWLKSKLMPSLPGVPFFSCQRGYRGTWRGDGICNTTCPCGASIAGHVKNGTMRIVGPRTCSNVWNGTFPI
6hCON   SSSWLRDIWDWVCEVLSDFKTWLKAKLVPALPGVPFLSCQRGFRGTWRGDGICHTTCPCGSEITGHVKNGTMKISGPRWCSNVSHRTFPI
6iCON   SGSWLRDIWDWVCMVLSDFKTWLKAKLVPALPGVPFFSCQRGFRGVWRGDGVCHTTCPCGANITGHVRNGTMKIAGPKWCSNIWHGTFPI
6kCON   SGSWLREIWDWVCTVLSDFKTWLRTKLVPALPGLPFLSCQRGFRGVWRGDGICHTTCPCGADIVGHVKNGSMRISGPRWCSNIWHGTFPI
6lCON   AGSWLRDIWDWICTVLSDFKSWLKTKLVPALPGVPFLSCQRGFKGLWRGDGICRTTCPCGADIVGHVKNGSMRISGSRWCSNMWHGTFPI
6mCON   SGSWLRDIWDWVCTVLSDFKTWLKAKLVPSLPGVPFLSCQRGFRGVWRGDGVCHTTCTCGAVIAGHVRNGTMKISGSRWCSNVWHGTFPI
6m2CON  SGSWLRDIWDWVCTVLSDFKTWLKAKLVPSLPGVPFLSCQRGFRGVWRGDGVCHTTCTCGAVIAGHVRNGTMKISGSRWCSNVWHGTFPI
6nCON   SGSWLRDIWDWVCTVLSDFKTWLKAKLVPSLPGVPFLSCQRGFRGTWRGDGICHTTCTCGAMIVGHVKNGSMRISGSRWCSNIWHGTFPI
6oCON   STSWLRDIWDWVCTVLSDFKTWLKVKLLPALPGIPFLSCQRGFRGVWRGDGICYTTCACGASITGHVKNGTMKIVGPKTCSNVWHGTFPI
6pCON   STTWLRDIWDWVCTVLSDFKTWLKAKLVPSLPGIPFLSCQRGFRGVWRGEGINHTTCPCGAMITGHVKNGSMKISGPKTCSNVWHGRFPI
6rCON   ATSWLKDIWDWVCVVLSDFKEWLKGKLTPALPGIPFLSCQRGYQGVWRGDGVCTTTCPCGAAITGHVKNGSMRIVGSKWCSNVWHGRFPI
6r2CON  ATSWLRDIWDWVCVVLSDFKEWLKGKLTPALPGIPFLSCQRGYQGVWRGDGVCTTTCPCGAAITGHVKNGSMRIVGSKWCSNVWHGRFPI
6tCON   ATSWLRDIWDWVCTVLSDFKTWLKAKLVPSLPGIPFLSCQRGFRGVWRGDGVCYTTCGCGANITGHVKNGSMKIAGPRTCSNVWHGRFPI
6uCON   ALSWLRDVWDWVCTVLSDFKTWLKAKLVPSLPGIPFFSCQRGFRGTWRGDGICYTTCPCGGHITGHVRNGTMKITGPKTCN.VWHGTFPI
6vCON   SGSWLRDIWDWVCTVLSDFKVWLKSRLVPSLPGIPFLSCQKGFRGEWRGDGICTTTCPCGAQITGHVKNGTMKLHGPRTCSNIWHGTFPI
6wCON   STSWLKDVWDWVCTVLSDFKTWLKAKLFPALPGIPFYSCQRGYKGTWKGDGVCHTTCPCGADIAGHVKNGSMKILGPRTCSNVWHGTFPI
6w2CON  STSWLKDIWDWVCTVLSDFKTWLKAKLFPALPGIPFYSCQRGYKGTWRGDGVCHTSCPCGADIAGHVKNGSMKIVGPRTCSNVWHGTFPI
```

```
         100       110       120       130       140       150       160       170       180
1aCON    NAYTTGPCTPLPAPNYTFALWRVSAEEYVEIRQVGDFHYVTGMTTDNLKCPCQVPSPEFFTELDGVRLHRFAPPCKPLLREEVSFRVGLH
1bCON    NAYTTGPCTPSPAPNYSRALWRVAAEEYVEVTRVGDFHYVTGMTTDNVKCPCQVPAPEFFTEVDGVRLHRYAPACKPLLRDEVTFQVGLN
1cCON    NAYTTGPCTPSPAPNYTFALWRVSAEEYVEVRRLGDFHYITGVTTDKIKCPCQVPSPEFFTEVDGVRLHRYAPPCKPLLREEVTFSIGLN
1eCON    NTYTTGPCTPAPAPNYTRALWRVSAEEYVEIRRVGDFHYVTGMTTDNIKCPCQVPSPEFFTELDGVRLHRYAPPCKPLLRDEVSFRVGLN
1e2CON   NTYTTGPCTPSPAPNYTRALWRVSAEEYVEVRRVGDSHYVTGMTSDNLKCPCQVPSPEFFTELDGVRIHRYAPPCKPLLRDEVVFTVGLN
1gCON    NAFTTGPCTPTPAPNYSRALWRVSAEEYVEVRRVGDFHYVTGMTADNIKCPCQVPSPEFFTELDGVRLHRYAPPCKPLLREEVTFQVGLN
1lCON    NAYTTGPCVPVPTPDYSRALWRVSAEEYVEIRRVGDFHYVTGMTTDNIKCPCQVPSPEFFTEVDGVRLHRYAPPCKPLLREEVSFQVGLH
2aCON    NCYTEGQCVPKPAPNFKTAIWRVAASEYAEVTQHGSYSYITGLTTDNLKVPCQLPSPEFFSWVDGVQIHRFAPTPKPFFRDEVSFCVGLN
2bCON    NCYTEGPCVPKPPPNYKTAIWRVAASEYVEVTQHGSFSYVTGLTSDNLKVPCQVPAPEFFSWVDGVQIHRFAPTPGPFFRDEVTFTVGLN
2cCON    NCYTEGPCVPKPAPNFKTAIWRVAASEYAEVTQHGSHAYVTGLTADNLKVPCQLPCPEFFSWVDGVQIHRFAPTPKAFMRDEVSFSVGLN
2eCON    NSYTEGQCVPKPSPNFKTAIWRVAATEYAEVTQHGQYAYVTGLTADNLKVPCQIPQPEFFSWVDGVQIHRHAPQPKPFFRDEVSFSVGLN
2iCON    NCYTEGQCVPKPAPNFKTAIWRVAATEYAEVTRHGSYAYVTGLTTDNLKVPCQIPSPEFFSWVDGVQIHRFAPTPKPFIRDEVSFSVGLN
2jCON    NCYTEGQCVPKPAPNFKTAIWRVAASEYAEITQHGPYAYVTGLTTDNLKVPCQLPAPEFFSWVDGVQIHRFAPTPKPLIRDEVSFSVGLN
2kCON    NCYTEGQCSPQPAHNYKTALWRVAAEEYAEVTRVGSFHYVTGLTTDNLKVPCQLPAPEFFTEVDGVRLHRYAPAPKPLLRDEVSFTVGLN
2lCON    NSYTEGPCAPRPPPNYKTAIWRVAARDYAEVTRVGDNHYVTGLTTDDLKCPCQVPSPEFFSWIDGVQIHRFAPPPGPFFRDEVNFSVGLH
2l2CON   NSYTEGPCVPRPPPNYKTAIWRVAAQDYAEVTRVGDNHYVTGLTTDDLKCPCQVPSPEFFSWVDGVQIHRFAPPPGPFFRDEVNFRVGLH
2mCON    NSYTEGQCVPKPSPNFKTAIWRVAATEYAEVTRHGQYAYVTGLTTDNLKVPCQLPAPEFFSWVDGVQIHRFAPTPNPLIRDEVSFSVGLN
2qCON    NCYTEGQCVPKPAPSYRTAIWRVAAADYVEVTRHGSYSYVTGLTNDNLKVPCQLPSPEFFSWVDGVQIHRFAPTPKPFIRDEVTFSVGLN
3aCON    NEYTTGPSTPCPSPNYTRALWRVAANSYVEVRRVGDFHYITGATEDELKCPCQVPAAEFFTEVDGVRLHRYAPPCKPLLRDEITFMVGLN
3bCON    NEYTTGPSTPVPAYNYSRALWRVASDSYVEVRREGDTHFVVGATNDGLKIPCQVPAPEFFTELDGVRLHRYAPPCKPLLRDEITFSVGLH
3b2CON   NEYTTGPSTPVPAYNYSRALWRVASDSYVEVRREGDTHFVVGATNDGLKIPCQVPAPEFFTELDGVRLHRYAPPCKPLLRDEITFSVGLH
3gCON    NEYTTGPSTPAPAYNYSRALWRVASDSYVEVRRVGDTHYVTGATDDGLKCPCQVPAPEFFTEVDGVRLHRYAPPCKPLLRDEICFSVGMH
3g2CON   NEYTTGPSTPAPAHNYSRALWRVASDSYVEVRRVGDAHYVIGATDDGLKCPCQVPAPEFFTELDGVRLHRYAPPCKPLLRDEICFSVGLH
3hCON    NEYTVGPCVPIPTENYSRALWRVAAEDYVEVKRVGDCHYIVGTTTDGLECPCQVPTAEFFTEVDGVRLHRYAPPCKPLLRDEVTFTVGLH
3iCON    NEYTTGPCVPTPAYNYSRGLWRVASDSYVEVRRVGDAHYIVGATDDNLKCPCQVPAPEFFTELDGVRLHRYAPPCRPLLRDEISFSVGLH
3kCON    NGYTTGPSTPAPSYAYSRALWRVASDSYVEVRKVGDFHYVTGTTDDGLKCPCQVPLPEFFTELDGVRLHRYAPVCRPLLRDDVTFTVGLN
4aCON    NAYTTGPGVPIPAPNYKFALWRVSAEEYVEVRRVGDFHYVTGVTQDNIKCPCQVPAPEFFTEVDGIRLHRHAPKCKPLLRDEVSFSVGLN
4bCON    NATTTGSGVPVPAPNYKFALWRVSAEEYVEVRRVGDFHYITGMTQDNIKCPCQVPAPEFFTEVDGVRLHRYAPDCKPLLREEVSFSVGLN
4b2CON   NASTTGAGVPIPAPNYKFALWRVSAEEYVEIRRVGEFHYVTGMTQDNIKCPCQVPAPEFFTEVDGVRLHRYAPPCKPLLRDEVQFTVGLN
4cCON    NAYTTGPGIPIPAPNYKFALWRVSAEEYVEVRRVGDFHYITGVTQDNIKCPCQVPAPEFFTEVDGIRIHRHAPACKPLLREEVTFSVGLN
4dCON    NAYTTGPSVPIPAPDYKVALWRVSAEEYVEVRRVGDFHYVTGVTQDNIKCPCQVPAPEFFTEVDGVRLHRHAPACKPLLRDEVTFSVGLN
4fCON    NAYTTGPGVPIPAPNYTFALWRVSAEEYVQVRRVGDFHYITGVTQDKIKCPCQVPAPEFFTEVDGVRIHRHAPECKPLLTEEVTFSVGLN
4gCON    NARTTGPGVPVPAPNYKFALWRVSAEEYVEVRRVGDYHYVVGVTQDNIKCPCQVPAPEFFTEVDGVRLHRYAPPCKPLLRDEVSFSVGLN
4kCON    NAYTSGPSVPIPAPNYKFALWRVSAEEYVEVRRVGDYHYVVGVTQDNIKCPCQVPSPEFFTEVDGVNLHRYAPPCKPLLRDEVTFSVGLN
4lCON    NAYTTGPGVPIPAPNYKFALWRVSAEEYVEVRRVGEFHYITGVTQDNIKCPCQVPAPEFFSEVDGVRLHRFAPPCKPLLRDEVAFTVGLN
4l2CON   NAYTTGPGIPIPAPNYEFALWRVSAEEYVEVRRVGEFHYITGVTQDNIKCPCQVPAPEFFTEVDGVRLHRFAPPCKPLLRDEVTFTVGLN
4mCON    NAYTTGPGVPIPAPNYKFALWRVAAEEYVEVRRVGDFHYITGVTQDTIKCPCQVPSPEFFTEVDGVRIHRHAPDCKPLLRDEVTFTVGLN
4m2CON   NAYTTGPGVPVPAPNYKFALWRVSAEDYVEVRRVGDFHYITGVTQDNIKCPCQVPSPEFFTEVDGVRIHRHAPDCKPLLRDEVTFTVGLN
4nCON    NAYTTGPGVPIPAPNYKFAMWRVSAEEYVEVRRVGDYHYITGVTQDNIKCPCQVPAPEFFTEVDGVRLHRFAPPCKPLLRDEVTFSVGLN
4oCON    NAYTTGPGVPIPAPNYEFALWRVSAEEYVEVRRVGEFHYVTGMTQDNIKCPCQVPAPEFFTEVDGVRLHRHAPKCKPLLREEVSFSVGLN
4pCON    NAYTTGPGVPIPAPNYKFALWRVAAEEYVEVRKVGDSHYVTGVTQDNIKCPCQVPSPEFFTEVDGVRLHRHAPSCKPLLREEVCFTVGLN
4qCON    NAYTTGPGVPIPAPNYKFALWRVSAEEYVEVRRVGDFHYITGVTQDNIKCPCQVPAPEFFTEVDGVRLHRHAPPCKPLLRDEVAFMVGLN
4rCON    NAYTTGPGVPIPAPNYKFALWRVSAEEYVEVRRVGDYHYVTGVTQDNIKCPCQVPSPEFFTEVDGVALHRYAPPCKPLLRDEVSFSVGLN
4sCON    NAYTTGPSIPIPAPNYQFALWRVSAEEYVEVRRVGEFHYITGMTQDHLKCPCQVPAPEFFTEVDGVRLHRHAPPCKPLLRDEVVFTVGLN
4vCON    NAYTTGPGVPIPAPNYKFALWRVSAEEYVEVRRVGDFHYITGVTQDNIKCPCQVPAPEFFTEVDGVRLHRHAPPCKPLLRDEVAFTVGLN
5aCON    NATTTGPSVPAPAPNYKFALWRVGAADYAEVRRVGDYHYITGVTQDNLKCPCQVPSPEFFTELDGVRIHRFAPPCNPLLREEVSFSVGLH
6aCON    NATTTGPSTPRPAPNYQRALWRVSAEDYVEVRRLGDCHYVVGVTAEGLKCPCQVPAPEFFTEVDGVRIHRYAPPCKPLLRDEVTFSVGLS
6bCON    NATTTSPSVPVPAPNYKRALWRVSAEEYVEVERHGDRHYVVGVTADGLKCPCQVPGPEFFTEVDGVRIHRYAPPCKPLLRDEVSFSVGLL
6dCON    NAITTGPSVPVPEPNYHKALWRVSAEDYVEVVRVNDHHYIVGATADNLKCPCQVPAPEFFTEVDGVRLHRFAPPCRPLMRDDITFSVGLS
6eCON    NATTTGPSIPIPEPNYKRALWRVSAEDYVEVVQVKDCHYIVGATTDNLKCPCQVPAPEFFTEVDGVRLHRYAPACGPLLRDEVSFSVGLH
6e2CON   NATTTGPSVPIPEPNYKRALWRVSAEDYVEVVQIKDCHYIVGATTDNLKCPCQVPAPEFFTEVDGVRLHRYAPACGPLLRDEISFSVGLH
6fCON    NATTTGPSVPIPSPNYHKALWRVAAEEYVEVVRHGDNHYIVAATSDNLKCPCQVPSPEFFTEVDGVRLHRYAPACRPLLRDEITYSVGLS
6gCON    NATTTGPSIPIPAPNYKKALWRVSATEYVEVVRVGDSHYITGVTAENTKCPCQVPAPEFFTEVDGVRLHRYAPECKPILRDEVTFTVGLS
6hCON    NATTTGPSVPIPEPNYTRALWRVSAEEYVEVKRVGDSHFVVGATTDNLKCPCQVPAPEFFTEVDGVRLHRYAPRCKPLLRDEVSFSVGLS
6iCON    NATTTGPSVPIPEPNYTRALWRVSAEDYVEVKRLGDSHFVVGATEDNLKCPCQVPAPEFFTEVDGVRLHRYAPKCKPLLRDEITFSVGMS
6kCON    NATTTGPSVPIPEPNYKRALWRVSAEEYVEVVRMGDSHFVVGATNQDLKCPCQVPAPEFFTEVDGVRLHRYAPPCKPLLRDEISFSVGLN
6lCON    NATTTGPSVPIPEPNYKRALWRVSAEEYVEIARVGDSHFVVGATNQDLKCPCQVPAPEFFTEVDGVRLHRYAPPCKPLLRDEISFSVGLN
6mCON    NASTTGPSVPIPEPNYKRALWRVAAEEYVEVVRVGDCHFVVGATNQDLKCPCQVPAPEFFTEVDGVRIHRYAPPCKPLLRDEVSFSVGLS
6m2CON   NASTTGPSVPIPEPNYKRALWRVAAEEYVEVVRVGDCHFVVGATNQDLKCPCQVPAPEFFTEVDGVRIHRYAPPCKPLLRDEVSFSVGLS
6nCON    NASTTGPSVPIPEPNYTRALWRVSAEEYVEVARKGDSHFIVGVTNQDLKCPCQVPAPEFFTEVDGVRIHRYAPECKPMLRDEVTFSVGLS
6oCON    NASTTGPSVPIPEPNYRKALWRVAAEEYVEVLHINDCHYVVGATADNLKCPCQVPAPEFFTEVDGVRLHRYAPACRPLLREEVTFSVGLN
6pCON    NATTTGPSVPIPDPNYKRALWRVSAEDYVEVVRIHDCHYVVGATADNLKCPCQVPAPEFFTEVDGIRLHRYAPTCRPLLRDELTFSVGLN
6rCON    NATTTGPSVPIPSPNYRRALWRVAAEEYVEVLKHQDNHYIVGVTSDNLKCPCQVPAPEFFTEVDGVRLHRYAPTCRPLLREEVTFSVGLS
6r2CON   NATTTGPSVPIPSPNYRRALWRVAAEEYVEVLKHQDNHYIVGVTSENLKCPCQVPAPEFFTEVDGVRLHRYAPTCRPLLREEVTFSVGLS
6tCON    NATTTGPSVPIPEPNYRTALWRVSAEDYVEVVRINDCHYVVGATADNLKCPCQVPAPEFFTMVDGVRLHRYAPECRPLIRDEVTFSVGLS
6uCON    NASTTGPSVPVPEPNYKRALRRVSAEDYVEVIRVKDCHYVVGATADNLKCPCQVPAPEFFTEVDGVRIHRYAPACRPLLRDEVSFSVGLH
6vCON    NASTTGPSVPIPTPDYNRALWRVSAEEYVEIRRIGDSHFVVGLTNDNLKCPCQVPAPEFFTEVDGVRLHRYAPPCKPLMRDEVTFSVGLN
6wCON    NATTTGPSVPTPSPNYTRALWRVAAEEYVEVLRVEDSHYVVGVTADNVKCPCQVPAPEFFTEIDGVRIHRYAPRCKPLLKDEVSFSVGLN
6w2CON   NATTTGPSIPTPSPNYTRALWRVAAEEYVEVLKVRDNHYVVGVTADNVKCPCQVPAPEFFTEIDGVRIHRYAPRCKPLLKDEVSFSVGLN
```

```
                    190       200       210       220       230       240       250       260       270
1aCON     EYPVGSQLPCEPEPDVAVLTSMLTDPSHITAEAAGRRLARGSPPSVASSSASQLSAPSLKATCTANHDSPDAELIEANLLWRQEMGGNIT
1bCON     QYLVGSQLPCEPEPDVAVLTSMLTDPSHITAETAKRRLARGSPPSLASSSASQLSAPSLKATCTTHHDSPDADLIEANLLWRQEMGGNIT
1cCON     EYLVGSQLPCEPEPDVAVLTSMLTDPSHITAETAARRLKRGSPPSLASSSASQLSAPSLKATCTTHHDSPDADLIEANLLWRQEMGGNIT
1eCON     TYVVGSQLPCEPEPDVTVLTSMLTDPSHITAETAKRRLGRGSPPSLASSSASQLSAPSLKATCTTHHDSPDAELIEANLLWRQEMGGNIT
1e2CON    TFVVGSQLPCEPEPDVAVLTSMLTDPSHITAETAKRRLGRGSPPSLASSSASQLSAPSLKATCTTHHDSPDADLIEANLLWRQEMGGNIT
1gCON     SYVVGSQLPCEPEPDVAVLTSMLTDPSHITAETARRRLTRGSPPSLASSSASQLSAPSLKATCTTHHDSPDADLIEANLLWRQEMGGNIT
1lCON     SYVVGSQLPCEPEPDVTVLTSMLTDPSHITAEAAKRRLARGSPPSLASSSASQLSAPSLKATCTIAHDSPDAELIEANLLWRQEMGGNIT
2aCON     SFVVGSQLPCDPEPDTDVLMSMLTDPSHITAEAAARRLARGSPPSEASSSASQLSAPSLRATCTTHGKAYDVDMVDANLF....MGGDVT
2bCON     SFVVGSQLPCDPEPDTEVLASMLTDPSHITAEAAARRLARGSPPSQASSSASQLSAPSLKATCTTHKMAYDCDMVDANLF....MGGDVT
2cCON     SYVVGSQLPCEPEPDTEVLASMLTDPSHITAEAAARRLARGSPPSEASSSASQLSAPSLRATCTTHAKGPDIDMVDANLF....MGGSVT
2eCON     TFVVGSQLPCDPEPDTEVLASMLTDPSHITAEAAARRLARGSPPSEASSSASQLSAPSLRANCATHAKACDLDMVDANLFCWCSMGGDIT
2iCON     SFAVGSQLPCDPEPDVEVLASMLTDPSHITAEAAARRLARGSPPSEASSSASQLSAPSLRATCTTHARNPDIDMVDANLF....MGGEVT
2jCON     SFVVGSQLPCEPEPDTEVLASMLTDPSHITAEAAARRLARGSPPSEASSSASQLSAPSLRATCTTHGRGPDIDMVDANLF....MGGDVT
2kCON     QYVVGSQLPCEPEPDTEVLASMLTDPSHITAEAAKRRLARGSPPSLASSSASQLSAPSLKATCTTHAKSPAAELIEANLLWGQEVGGIIT
2lCON     SYVVGSQLPCEPEPDVAALTSMLTDPSHITAEAAARRLARGTAPSEASSSASQLSAPSLKASCTHYKGGPDTDMVDANLF....LRGSVT
2l2CON    SYVVGSQLPCEPEPDVAVLTSMLTDPSHITAETAARRLARGTTPSEASSSASQLSAPSLKASCTHYKGGPDTDMVDANLF....LGGSVT
2mCON     SFVVGSQLPCEPEPDTEVLASMLTDPSHITAEAAARRLARGSPPSEASSSASQLSAPSLRATCTTHGKGYDIDMVDASLF....MGGDVT
2qCON     SFVVGSQLPCEPEPDTEVLASMLTDPSHITAEAAARRLARGSPPSEASSSASQLSAPSLRATCTTHGKNYDIDMVDANLF....MGGDVT
3aCON     SYAIGSQLPCEPEPDVSVLTSMLRDPSHITAETAARRLARGSPPSEASSSASQLSAPSLKATCQTHRPHPDAELVDANLLWRQEMGSNIT
3bCON     SYAIGSQLPCEPEPDVAVLTSMLRDPAHITAATAARRLARGSPPSEASSSASQLSAPSLKATCQTHRPHPDAELIDANLLWRQEMGSNIT
3b2CON    SYAIGSQLPCEPEPDVAVLTSMLRDPAHITAATAARRLARGSPPSEASSSASQLSAPSLKATCQTHRPHPDAELIDANLLWRQEMGSNIT
3gCON     TYTIGSMLPCEPEPDVAVLTSMLQDPSHITAQTAARRLARGSPPSEASSSASQLSAPSLKATCQSYHPHPDVELVEANLLWRQEMGSNIT
3g2CON    TYTIGSRLPCEPEPDVAVLTSMLRDPSHITAQTAARRLARGSPPSEASSSASQLSAPSLKATCKSHRPHPDAELVDANLLWRQEMGSNIT
3hCON     TYVIGAQLPCEPEPDVTVLTSMLRDPSHITAEAAARRLARGSPPSLASSSASQLSAPSLYATCNTHGKLPDAELVDANLLWRQEMGSNIT
3iCON     SYAIGSQLPCEPEPDVSVLTSMLRDPSHITAETAARRLARGSPPSEASSSASQLSAPSMKATCKTHGPHPDVELIDANLLWRQEMGSNIT
3kCON     SYVIGSQLPCEPEPDVAVVTSMLQDPSHITVETAKRRLDRGSPPSLASSSASQLSAPSRKATCTTHGRHPDAELITANLLWRQEMGSNIT
4aCON     SFVVGSQLPCEPEPDVAVLTSMLTDPSHITAETASRRLARGSPPSLASSSASQLSAPSLKATCTARHDSPGTDLLEANLLWGQE..STAT
4bCON     TFVVGSQLPCEPEPDVHVLTSMLTDPSHLTAETARRRLARGSPPSLASSSASQLSAPSLKATCTAHHDSPDADLIEANLLWRQEMGSNIT
4b2CON    TFVVGSQLPCEPEPDVQVLTSMLTDPSHVTAEAAARRLARGSPPSEASSSASQLSAPSLKATCTTNHHSPDADLIEANLLWRQEMGGNIT
4cCON     SFVVGSQLPCEPEPDVAVLTSMLTDPSHITAEAARRRLTRGSPPSLASSSASQLSAPSLKATCAA.HDSPGTDLIEANLLWG....SNAT
4dCON     TFVVGSQLPCEPEPDVAVLTSMLTDPSHITAEAARRRLGRGSPPSLASSSASQLSAPSLKATCTDHKDSPGVDLIESNLLWG....ANAT
4fCON     SFVVGSQLPCEPEPDVAVLTSMLTDPSHITAETARRRLARGSPPSLASSSASQLSAPSLKATCTAHHSSPGQDLIEANLLWG....ANAT
4gCON     TFVVGSQLPCEPEPDVAVLTSMLTDPSHITAESAARRLARGSPPSLASSSASQLSAPSLKATCTAHHDSPDADLIEANLLWRQEMGSNIT
4kCON     TFVVGSQLPCEPEPDVAVLTSMLTDPSHITAATAARRLARGSPPSLASSSASQLSAPSLKATCTTHHESPDADLIEANLLWRQEMGSNIT
4lCON     SFVVGSQLPCEPEPDVTVVTSMLTDPSHLTAETARRRLARGSPPSLASSSASQLSAPSLKATCTAHPDFPGSDLIEANLLWCSEYGAGAK
4l2CON    SFAVGSQLPCEPEPDVAVLTSMLTDPSHLTAETARRRLARGSPPSLASSSASQLSAPSLKATCTAHHDSPGNDLIEANLLWG....TKAT
4mCON     SFVVGSQLPCEPEPDVAVLTSMLTDPSHITAETARRRLARGSPPSLASSSASQLSAPSLKATCPADHDSPGTDLIEANLLWG....ASAT
4m2CON    SFVVGSQLPCEPEPDVTVLTSMLTDPSHMTAETARRRLARGSPPSLASSSASQLSAPSLKATCPAGHDSPGADLIEANLLWG....VGAA
4nCON     TFVVGSQLPCEPEPDVSVLTSMLTDPSHITAEAAGRRLARGSPPSLASSSASQLSAPSLKATCTAHHDSPGTDLIESNLLWG....TGVT
4oCON     SFVVGSQLPCEPEPDVAVLTSMLTDPSHVTAEAASRRLARGSPPSLASSSASQLSAPSLKATCAA.HDTPGSDLIEANLLWG....SNVT
4pCON     TFVVGSQLPCEPEPDVSVLTSMLTDPSHITAEAAGRRLARGSPPSLASSSASQLSAPSLKATCPANHDSPGTDLIEANLLWG....NSVT
4qCON     SFVVGSQLPCEPEPDVAVLTSMLTDPSHITAETAGRRLARGSPPSLASSSASQLSAPSLKATCTKHHDSPGTDLIEANLLWG....TGVI
4rCON     TFVVGSQLPCEPEPDVAVLTSMLTDPSHVTAEAAKRRLDRGSPPSLASSSASQLSAPSLKATCTTHHDSPDADLIEANLLWRQEMGGNIT
4sCON     SYVVGSQLPCEPEPDVAVLTSMLTDPGHMTAETAKRRLARGSPPSLASSSASQLSAPSLKATCTTHHDSPDADLIEANLLWRQEMGSNIT
4vCON     TFVVGSQLPCEPEPDVAVLTSMLTDPSHVTAETARRRLARGSPPSLASSSASQLSAPSLKATCTAHQDSPGADLIEANLLWG....TAAT
5aCON     SYVVGSQLPCEPEPDVTVLTSMLSDPAHITAETAKRRLDRGSPPSLASSSASQLSAPSLKATCTTQGHHPDADLIEANLLWRQCMGGNIT
6aCON     NYAIGSQLPCEPEPDVTVVTSMLTDPTHITAETAARRLKRGSPPSLASSSASQLSAPSLKATCTTSKDHPDMELIEANLLWRQEMGGNIT
6bCON     EFVVGSQLPCEPEPDVTVVTSMLTDPSHITAETASRRLKRGSPPSLASSSASQLSAPSLKATCTANGDHPDAELIEANLLWRQEMGSNIT
6dCON     TYVVGSQLPCEPEPDVVILTSMLTDPDHITAETAARRLARGSPPSLASSSASQLSAPSLKATCTTAGKHPDAELIEANLLWRQEVGGNIT
6eCON     SYAMGSQLPCEPEPDTMVLTSMLSDPTHITAETAGRRLARGSPPSLASSSASQLSAPSLKATCTTAVDHPDAELIEANLLWRQEVGGNIT
6e2CON    SYAMGSQLPCEPEPDAIVLTSMLSDPTHITAETAGRRLARGSPPSLASSSASQLSAPSLKATCTTAVDHPDAELIEANLLWRQEVGGNIT
6fCON     TYIIGSQLPCEPEPDVAVLTSMLTDPAHITAETAGRRLARGSPPSLASSSASQLSAPSLKATCTTADRHPDAELIEANLLWRQEVGGNIT
6gCON     TYVVGSQLPCEPEPDVLVVTSMLRDPDHITAEEASRRLKRGSPPSLASSSASQLSAPSLKATCTTHADHPDAELVEANLLWRQEMGGNIT
6hCON     SYAVGSQLPCEPEPDVTVVTSMLIDPSHVTAEAAARRLARGSPPSLASSSASQLSAPSLKATCTMHGAHPDAELIEANLLWRQEMGGNIT
6iCON     SYAMGSQLPCEPEPDVVVATSMLTDPSHMTAETAARRLARGSPPSLASSSASQLSAPSLKATCTTHGPHPDAELIEANLLWRQEMGNNIT
6kCON     SYAIGSQLPCEPEPDVTVVTSMLVDPSHITAETAARRLGRGSPPSLASSSASQLSAPSLKATCTTHGVHADADLIEANLLWRQEVGGNIT
6lCON     PYAIGSQLPCEPEPDVMVVTSMLVDPSHITAEAAARRLGRGSPPSLASSSASQLSAPSLKATCTTHGAHPDADLIEANLLWRQEVGGNIT
6mCON     SYAIGSQLPCEPEPDVMVVTSMLTDPSHVTAEAAGRRLARGSPPSLASSSASQLSAPSLKATCTTHGPHPDAELIEANLLWRQEVGGNIT
6m2CON    SYAIGSQLPCEPEPDVMVVTSMLTDPSHVTAEAAGRRLARGSPPSLASSSASQLSAPSLKATCTTHGPHPDAELIEANLLWRQEMGSNIT
6nCON     SYAVGSQLPCEPEPDVMVVTSMLTDPSHITAESAARRLARGSPPSLASSSASQLSAPSLKATCTTHGQHPDAELIEANLLWRQEMGSNIT
6oCON     SYAMGSQLPCEPEPDVMVLTSMLTDPDHITAEEARRRLARGSPPSLASSSASQLSAPSLRATCTAAGDHPDAELIEANLLWRQEVGGNIT
6pCON     SYAMGSQLPCEPEPDVMILTSMLSDPSHITAETAARRLARGSPPSLASSSASQLSAPSLKATCTTARDHPDAELVEANLLWRQEMGGNIT
6rCON     TYVIGSQLPCEPEPDVAVLTSMLTDPAHITAETARRRLGRGSPPSQASSSASQLSAPSLKATCTTAGRHPDAELLEANLLWRQEVGGNIT
6r2CON    TYVIGSQLPCEPEPDVAVLTSMLTDPAHITAETARRRLGRGSPPSQASSSASQLSAPSLKATCTTAGRHPDAELLEANLLWRQEVGGNIT
6tCON     TYAIGSQLPCEPEPDVVTVTSMLVDPAHITAETARRRLNRGSPPSLASSSASQLSAPSLKATCTTAGAHPDAELIEANLLWRQEVGGNIT
6uCON     SYVVGSQLPCEPEPDEMTLTSMLTDPSHITAETARRRLARGSPPSLASPSASQLSAISLKATCTVPAGHPDAELLEANLLWGKELGGNIT
6vCON     TFAVGSQLPCDPEPDVVALTSMLTDPSHVTAEAARRRLARGSPPSLASSSASQLSAPSLKGTCTTHRPHPDAELLEANLLWRQEVGGNIT
6wCON     TYVVGSRLPCDPEPDVLVVTSMLTDPDHITAEAAGRRLARGSPPSLASSSASQLSAPSLKATCTTHCDDPDAELISANLLWRQEMGGNIT
6w2CON    TYVVGSQLPCDPEPDVLVVTSMLTDPDHITAEAAGRRLARGSPPSLASSSASQLSAPSLKATCTTHREHPDAELISANLLWRQEMGGNIT
```

```
                280       290       300       310       320       330       340       350       360
1aCON    RVESENKVVILDSFDPLVAEEDEREISVPAEILRKSRRFAPALPIWARPDYNPPLLETWKKPDYEPPVVHGCPLPPPQSPPVPPPRKKRT
1bCON    RVESENKVVILDSFDPLRAEEDEREVSVPAEILRKSRKFPPAMPIWARPDYNPPLLESWKDPDYVPPVVHGCPLPPTKAPPIPPPRKKRT
1cCON    RVESENKIVILDSFDPLVAEEDDREISVPAEILRKSKKFPPAMPIWARPDYNPPLVEPWKRPDYEPPLVHGCPLPPPKPTPVPPPRKKRT
1eCON    RVESENKVVILDSFDPLVAAQDDREISVPAEILRKTKKFPASIPVWASPDYNPPLLETWKKPDYEPPVVHGCPLPPAKPPPVPPPRRRRQ
1e2CON   RVESENKVVILDSFDPLVAEEDDREVSVPAEILRKTKRFPSCLPVWASPSYNPPLLETWKEPDYEPPVVHRCPLPPAKPPPVPPPRRRKQ
1gCON    RVESENKVVILDSFDPLVPEYDDREVSVPAEILRKTKKFPSSLPVWASPSYNPPLLEPWKKPGYEPPVVHGCPLPPAKTPPIPPPRRRKQ
1lCON    RVESENKVVILDSFDPLKAEDDDREVSVPAEILRKTKKFPQALPVWASPSYNPPLLEVWKRPDYEPPVVHGCALPPPGQPPVPPPRRKKT
2aCON    RIESESKVVVLDSLDPMAEEESDLEPSIPSEYMLPKKRFPPALPAWARPDYNPPLVESWKRPDYQPPTVAGCALPPPKKTPTPPPRRRRT
2bCON    RIESDSKVIVLDSLDSMTEVEDDREPSVPSEYLIRRKKFPPALPPWARPDYNPPVIETWKRPDYEPPTVLGCALPPTPQAPVPPPRRRRA
2cCON    RIESQSKVLVLDSFDPVVDEEDEREPSIPSEYLLPRSKFPPALPPWARPDYNPPLLETWKRPDYQPPVVAGCALPPSGTTPVPPPRRRRA
2eCON    RVESESKVLILDSLDPVVEKESELEPSIPSEYLLPKKRFPPALPPWARPDYNPPLLETWKRPDYKPPVVAGCALPPPKQAPVPPPRRRRA
2iCON    RIESESKVIMLDSLDSMAEEEDEREPSVPSEYLLPREKFPLAIPLWARPDYNPPVVETWKRPDYQPPTVAGCALPPPGQTPVPPPRRRRA
2jCON    RVESDAKVLILDSLDPMVEEEDEREPSVPSEYLLPRKRFPPALPPWARPDYNPPLLENWKKPDYQPPTVAGCALPPSQQAPVPPPRRRRA
2kCON    EVKVLNLVVLLPSVEEEDEEEPEREVEVLAEILKKSRKLPRAARIDARPPYNEPLLRPDKDPPTVSGVALGPPLPPTTAPPIRRRRKLT
2lCON    RIESDSKVVVLDSFDPPSGEEDERGPSIPSEYLLPRKAFPAALPVWARPDYNPPTVESWKRADYTAPVVHGCAIAPPKPAPVPPPRRRRT
2l2CON   RIESNSKVVVLDSFDPPSGEEDEREPSIPSEYLLPRKAFPAALPVWARPDYNPPTVESWKRADYTAPVVHGCAIAPPKPVPVPPPRRRRT
2mCON    RVESESKVLILDSLDPMLEEEDEREPSIPSEYLLPGKKFPPALPVWARPDYNPPVLETWKKPDYEPPTVAGCALPPSTAAPTPPPRRRRA
2qCON    RIESESKVLVLDSLDPSIEEEDEREPSIPSEYLLPRKKFPPALPVWAQPSYNPPLIESWKKPDYEPPTVAGCALPPPTKAPTPPPRRRRA
3aCON    RVESETKVVILDSFEPLRAETDDAELSVAAECFKKPPKYPPALPIWARPDYNPPLLDRWKAPDYEPPTVHGCALPPRGAPPVPPPRRKRT
3bCON    RVESETKVVILDSFEPLRAEEDDTELSVPAECFKKPPKYPPALPIWARPDYNPPLLPPWKDPTYEPPVVHGCALPPTRPTPVPPPRRKKT
3b2CON   RVESETKVVILDSFEPLRAEEDDTELSVPAECFKKPPKYPPALPIWARPDYNPPLLPPWKDPTYEPPVVHGCALPPTRPTPVPPPRRKKT
3gCON    RVESETKVVILDSFEPLRAEEGDNELSVAADCFKKPPKYPPALPIWARPDYNPPLLELWKAPDYKPPTVYGCPLPPERPTPVPPPRKKRT
3g2CON   RVESETKVVILDSFEPLRAEEGDSELSVAAECFKKPPKYPPALPIWARPDYNPPLLETWKDPDYEPPTVHGCPLPPGRPTPVPPPRKKRT
3hCON    RVESETKVVILDSFEPLHAVHDDHEISEAADCFKKPPSYPPALPVWARPDYNPPLLEPWKAPGYEAPVVHGCPLPPASTTPVPPPRRKRT
3iCON    RVESETKVVILDSFEPLRAEAGGDEPSVAAECFKKPPKYPPALPIWARPDYNPPLIENWKDPGYEPPVVHGCSLPPARPTPVPPPRKKRT
3kCON    RVESESKVVILDSFEPLRACDDEDELSVAAECFKKPPKYPPALPIWARPDYNPPLVEPWKDPDYVPPTVHGCALPPQKLPPVPPPRRKRT
4aCON    RVETDEKVIILDSFEPCVAEDDDREVSVAAEILRPTKKFPPALPIWARPDYNPPLTETWKQQDYKPPTVHGCALPPSKQPPVPPPRRKRT
4bCON    RVESENKVVVLDSFEPLVAEPDDVEISTAAEVMRRVKKFPPALPIWARPDYNPPLLEPWKQPDYIPPVVHGCALPPNKPAPVPPPRRKRV
4b2CON   RVESENKVVVLDSFEPLVAEQDSGEVSVAAEILRRSKRFPSPLPIWARPDYNPPLIQPWKQPDYIPPVVHGCALPPSKPTPVAPPRRKRA
4cCON    RIETDNKVVILDSFEPLVAEDD.RETSVAAEILRPTKRFPPALPVWAQPSYNPPLVEKWKQPDYDPPVVHGCALPPSGQAPIPPPRRKRV
4dCON    RVESEDKVLILDSFEPLVAEDDDREISVPAEILRTSRKFPRAIPIWAQPAYNPPLIETWKQPDYKPPVVHGCALPPDKPTPVPPPRRKRA
4fCON    RVELGDKVMVLDSFEPLVAE.EDKEVSVAAEILRPTKKFPPAIPIWARPDYNPPLVESWKQPDYEPPVVHGCALPPSNQPPVPPPRRKKT
4gCON    RVESETKVVILDSFDPLVAETDDKEVSVAAEILRPKKKFPAAMPVWARPDYNPPLLETWKKPDYDPPVVHGCALPAGGLPPVPPPRRKRT
4kCON    RVESETKVVVLDSFDPLVAEPDDGEISVPAECLRPTKRFPKPLPVWARPDYNPPLLEVWKKPEYDPPAVHGCALPPGGLPPVPPPRRKRA
4lCON    RIETEDKVVVLDSFEPLVAEEDDRETSVAAEILQPKKRFPSALPVWARPEYNPPLLETWKRQDYSPPAVHGCALPPNKPTPVPPPRRKRT
4l2CON   RVETEDKVLVLDSFEPLVAEDEDREISVAAEILQPRKRFPPALPIWARPEYNPPLVETWKQQDYDPPAVHGCALPPSKPTPVPPPRRKRA
4mCON    RVETDAKVVVLDSFEPLRAEPDDREISVAAEVLRPSKRFPPALPIWARPDYNPPLVEPWKQPDYDPPVVHGCALPPSKTTPVPPPRRKRA
4m2CON   RVETDAKVVVLDSFEPLRAEADDREVSVAAEILRPSKRFPPALPIWARPDYNPPLVEPWKQPDYDPPVVHGCALPPSKPTPVPPPRRKRA
4nCON    RVESEGKVMVLDSFEPLVAEDD.REISGEAEILRPSRKFPPALPIWARPDYNPPLVERWKQPSYDPPVVHGCALPSEKPTPVPPPRRKRT
4oCON    RIESETKVVTLDSFEPIVAEDD.REVSVEAEILRTAKRFPAAIPIWARPEYNPPLVDTWKRPDYQPPVVHGCALPPSKPAPVPPPRRKRT
4pCON    RVESESKVMILDSFEPLVAEDD.REISVAAEILCPTKKFPIALPVWARADYNPPLVETWKRPDYEPPVVHGCALPPAGPAPVPPPRRKRM
4qCON    RIESEDKVLVLESFEP.MAEREGKEISVAAEILRPSKKFPPALPVWARADYNPPLVEAWKQPGYDPPVVHGCPLPPSSSAPVPPPRRKRT
4rCON    RVESENKVVVLDSFEPLVAEPDDREISTAADALRRVKKFPPALPIWARPDYNPPLLETWKRQDYDPPVVHGCALPPGKPPPVPPPRRKRV
4sCON    RVESESKVVILDSFDPLVAEPEDNEISAAAEILRRTKKFPPALPIWARPDYNPPLLETWKQPSYDPPVVHGCALPPSKPTPVPPPRRRRV
4vCON    RIETESKVVVLDSFEPLVAEDDDREVSVAAEILRPSKRFPPAMPIWARPEYNPPLVEKWKQQDYEPPVVSGCALPPSGATPVPPPRRKRT
5aCON    RVEAENKVVILDSFEPLKAEDDDREISVSADCFRRGPAFPPALPVWARPGYDPPLLETWKRPDYDPPQVSGCPLPPAGLPPVPPPRRKRT
6aCON    RVESENKVVVLDSFEPLTAEYDEREISVSAECHRPPHKFPPALPVWARPDYNPPLLQAWQMPGYEPPVVSGCAVAPPKPAPIPPPRRKRL
6bCON    RVESETKVVILDSFDPLVAEYDDREISVSAECHRPPPKFPPALPIWARPDYNPPLLQKWQMPGYEPPVVSGCALPPAKPTPIPPPRRKRL
6dCON    RVESENKIIVLDSFDPLIAETDDREISVGAECFNPPPKFPPALPVWARPDYNPPLLQPWKAPDYEPPLVHGCALPPKGLPPVPPPRKKRV
6eCON    RVESENKIIVLDSFDPLVAETDDREISVAAECHRPPPKFPPALPIWARPDYNPPLMETWKAPDYDPPTVSGCALPPKGLPPIPPPRRKRV
6e2CON   RVESENKIIVLDSFDPLVAETDDREISVSAECHRPPLKFPPALPIWARPDYNPPLIETWRAPDYDPPTVSGCALPPKGLPPIPPPRRKRV
6fCON    RVESENKVIVLDSFDPLVAETDDREISVAAECHRPPPKYPPALPIWARPDYNPPLLETWKKPDYDPPTVAGCALPPSGQPPVPPPRRKRT
6gCON    RVESENKIVILDSFEPLKAEFDDREISVAAECHRPPFKYPPALPVWARPDYNPPLLETWKAPDYDPPVVSGCALPPQGLPPVPPPRRKKL
6hCON    RVESENKVVILDSFDPLVPEFEEREMSVPAECHRPRPKFPPALPIWATPGYNPPVLETWKSPTYEPPVVHGCALPPSGPPPIPPPRRKKV
6iCON    RVESENKVIILDSFDPLVPEYDDREPSVSAECHRPTPKYPPALPVWARPDYNPPILEPWKSPDYNPPMVHGCAMPPPDKLPVPPPRRRKV
6kCON    RVESENKVIVLDSFDPLVPEYDDREPSVPAECHRPPPKFPPALPIWARPDYNPPLLETWKKPDYTPPLVHGCALPPAGQPPVPPPRRKKV
6lCON    RVESENKVIVLDSFDPLTPEYDDREPSVPAECHRPKPKYPPALPIWARPDYNPPLLETWKKPDYDPPLVHGCALPPPGPAPIPPPRRKKV
6mCON    RVESESKVIVLDSFDPIAPEYDDREPSIPAECHRPSPEYPPALPIWARPDYNPPLLETWRAPDYEPPQVHGCALPPPGNTPVPPPRRRKV
6m2CON   RVESESKVIVLDSFDPIAPEYDDREPSIPAECHRPPPEYPPALPIWARPNYNPPLLETWRAPDYEPPQVHGCALPPPGNTPVPPPRRRKV
6nCON    RVESETKVIVLDSFDPIIPEYDDREPSVPAECHRPNPKYPPALPIWARPDYNPPLLETWKAPDYEPPVVHGCALPPQKVTPIPPPRRKRT
6oCON    RVESENKVIVLDSFDPLVAEVDDREISVAAECHRPPPKFPPALPIWARPDYNPPLLETWKAPDYNPPTVSGCALPPKGLPPVPPPRRKKV
6pCON    RVESENKVVVLDSFDPLVAEPDDREISVAAECLRPSPKFPPALPIWARPDYNPPLLESWKAPDYSPPTVSGCALPPKGIPPVPPPRRKKV
6rCON    RVESENKIIVLDSFDPLVAETDDREISVAAECHRPPPKYPPALPIWARPDYNPPLLESWKAPDYDPPVVAGCALPPPGRPPVPPPRRKRM
6r2CON   RVESENKIIVLDSFDPLVAETDDREISVAAECHRPPPKYPPALPIWARPDYNPPLLESWKAPDYDPPVVAGCALPPPGRPPVPPPRRKRM
6tCON    RVESENKVIVLDSFDPLVAEVDDREISTAAECHRPPSKYPPALPIWARPDYNPPLLEVWKSPTYDPPLVSGCALPPKGLPPIPPPRRKKV
6uCON    RVESENKIIILDSFDPLVAEPDDREISTAAECHRPPPKFPPALPIWARPDYNPPLIETWKAPDYDPPTVSGCALPPKGLPPVPPPRRKKV
6vCON    RVESENKVIVLDSFDPITPEYDEREISVAASCHRPPPRFPPALPIWARPDYNPPLIESWKSPEYEPPMVAGCALPPAKQTPIPPPRRKRL
6wCON    RVESENKVVVLDSFEPLTAEYDDREISTAAECHRPRPAFPAALPVWARPDYNPPLLEPWKAPDYKPPMVSGCALPPTPSPPIPPPRRKKL
6w2CON   RVESENKVVVLDSFEPLKAEYDDREISTAAECHRPRPAFPPALPVWARPDYNPPLLEPWKAPDYEPPMVSGCALPPAPSPPVPPPRRKKL
```

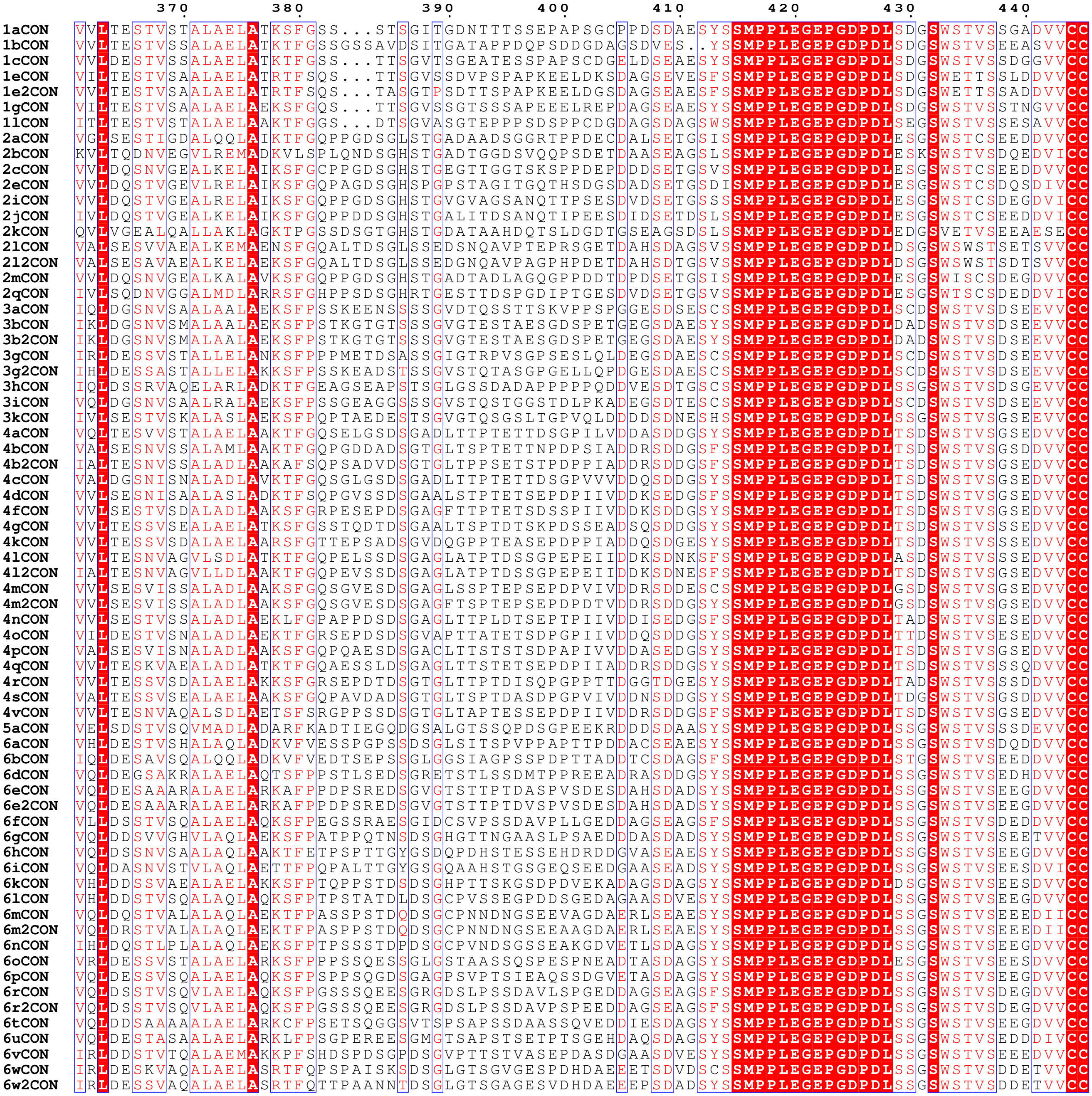
370 380 390 400 410 420 430 440
1aCON VVLTESTVSTALAELATKSFGSS...STSGITGDNTTTSSEPAPSGCPPDSDAESYSSMPPLEGEPGDPDLSDGSWSTVSSGADVVCC
1bCON VVLTESTVSSALAELATKTFGSSGSSAVDSITGATAPPDQPSDDGDAGSDVES..YSSMPPLEGEPGDPDLSDGSWSTVSEEASVVCC
1cCON VVLDESTVSSALAELATKTFGSS...TTSGVTSGEATESSPAPSCDGELDSEAESYSSMPPLEGEPGDPDLSDGSWSTVSSDGGVVCC
1eCON VILTESTVSAALAELATRTFSQS...TTSGVSSDVPSPAPKEELDKSDAGSEVESFSSMPPLEGEPGDPDLSDGSWETTSSLDDVVCC
1e2CON VVLTESTVSAALAELATRTFSQS...TASGTPSDTTSPAPKEELDGSDAGSEAESFSSMPPLEGEPGDPDLSDGSWETTSSADDVVCC
1gCON VILTESTVSAALAELAEKSFGQS...TTSGVSSGTSSSAPEEELREPDAGSEAESYSSMPPLEGEPGDPDLSDGSWSTVSSTNGVVCC
1lCON ITLTESTVSTALAELAAKTFGGS...DTSGVASGTEPPPSDSPPCDGDAGSDAGSWSSMPPLEGEPGDPDLSEGSWSTVSSESAVVCC
2aCON VGLSESTIGDALQQLAIKTFGQPPGDSGLSTGADAADSGGRTPPDECDALSETGSISSMPPLEGEPGDPDLESGSWSTCSEEDDVVCC
2bCON KVLTQDNVEGVLREMADKVLSPLQNDSGHSTGADTGGDSVQQPSDETDAASEAGSLSSMPPLEGEPGDPDLESKSWSTVSDQEDVICC
2cCON VVLDQSNVGEALKELAIKSFGCPPGDSGHSTGEGTTGGTSKSPPDEPDDDSETGSVSSMPPLEGEPGDPDLESGSWSTCSEEDDVVCC
2eCON VVLDQSTVGEVLRELAIKSFGQPAGDSGHSPGPSTAGITGQTHSDGSDADSETGSDISMPPLEGEPGDPDLESGSWSTCSDQSDIVCC
2iCON VVLDQSTVGEALRELAIKSFGQPPGDSGHSTGVGVAGSANQTTPSESDVDSETGSSSSMPPLEGEPGDPDLESGSWSTCSDEGDVICC
2jCON IVLDQSTVGEALKELAIKSFGQPPDDSGHSTGALITDSANQTIPEESDIDSETDSLSSMPPLEGEPGDPDLESGSWSTCSEEDDVICC
2kCON QVLVGEALQALLAKLAGKTPGSSDSGTGHSTGDATAAHDQTSLDGDTGSEAGSDSLSSMPPLEGEPGDPDLEDGSVETVSEEAESECC
2lCON VALSESVVAEALKEMAENSFGQALTDSGLSSEDSNQAVPTEPRSGETDAHSDAGSVSSMPPLEGEPGDPDLDSGSWSWSTSETSVVCC
2l2CON VALSESAVAEALKELAEKSFGQALTDSGLSSEDGNQAVPAGPHPDETDAHSDTGSVSSMPPLEGEPGDPDLDSGSWSWSTSDTSVVCC
2mCON VVLDQSNVGEALKALAVKSFGQPPGDSGHSTGADTADLAGQGPPDDTDPDSETGSISSMPPLEGEPGDPDLESGSWISCSDEGDVVCC
2qCON IVLSQDNVGGALMDLARRSFGHPPSDSGHRTGESTTDSPGDIPTGESDVDSETGSVSSMPPLEGEPGDPDLESGSWTSCSDEDDVICC
3aCON IQLDGSNVSAALAALAEKSFPSSKEENSSSGVDTQSSTTSKVPPSPGGESDSESCSSMPPLEGEPGDPDLSCDSWSTVSDSEEVVCC
3bCON IKLDGSNVSMALAALAEKSFPSTKGTGTSSSGVGTESTAESGDSPETGEGSDAESYSSMPPLEGEPGDPDLDADSWSTVSDSEEVVCC
3b2CON IKLDGSNVSMALAALAEKSFPSTKGTGTSSSGVGTESTAESGDSPETGEGSDAESYSSMPPLEGEPGDPDLDADSWSTVSDSEEVVCC
3gCON IRLDESSVSTALLELANKSFPPPMETDSASSGIGTRPVSGPSESLQLDEGSDAESCSSMPPLEGEPGDPDLSCDSWSTVSDSEEVVCC
3g2CON IHLDESSASTALLELAKKSFPSSKEADSTSSGVSTQTASGPGELLQPDGESDAESCSSMPPLEGEPGDPDLSCDSWSTVSDSEEVVCC
3hCON IQLDSSRVAQELARLADKTFGEAGSEAPSTSGLGSSDADAPPPPPQDDVESDTGSCSSMPPLEGEPGDPDLSSGSWSTVSDSGEVVCC
3iCON VQLDGSNVSAALRALAEKSFPSSGEAGGSSSGVSTQSTGGSTDLPKADEGSDTESCSSMPPLEGEPGDPDLSCDSWSTVSDSEEVVCC
3kCON IVLSESTVSKALASLAEKSFPQPTAEDESTSGVGTQSGSLTGPVQLDDDDSDNESHSSMPPLEGEPGDPDLSSGSWSTVSGSEEVVCC
4aCON VQLTESVVSTALAELAAKTFGQSELGSDSGADLTTPTETTDSGPILVDDASDDGSYSSMPPLEGEPGDPDLTSDSWSTVSGSEDVVCC
4bCON VALSESNVSSALAMLAAKTFGQPGDDADSGTGLTSPTETTNPDPSIADDRSDDGSFSSMPPLEGEPGDPDLTSDSWSTVSGSDDVVCC
4b2CON IALTESNVSSALADLAAKAFSQPSADVDSGTGLTPPSETSTPDPPIADDRSDDGSFSSMPPLEGEPGDPDLTSDSWSTVSGSEDVVCC
4cCON VALDGSNISNALADLAVKTFGQSGLGSDSGADLTTPTETTDSGPVVVDDQSDDGSYSSMPPLEGEPGDPDLTSDSWSTVSGSEDVVCC
4dCON VVLSESNISAALASLADKTFSQPGVSSDSGAALSTPTETSEPDPIIVDDKSEDGSFSSMPPLEGEPGDPDLTSDSWSTVSGSEDVVCC
4fCON VVLSESTVSDALADLAAKSFGRPESEPDSGAGFTTPTETSDSSPIIVDDKSDDGSYSSMPPLEGEPGDPDLTSDSWSTVSGSEDVVCC
4gCON VVLTESSVSEALAELATKSFGSSTQDTDSGAALTSPTDTSKPDSSEADSQSDDGSYSSMPPLEGEPGDPDLTSDSWSTVSSSEDVVCC
4kCON VVLTESSVSDALAELAARSFGTTEPSADSGVDQGPPTEASEPDPPIADDQSDGESYSSMPPLEGEPGDPDLTSDSWSTVSSSEDVVCC
4lCON VVLTESNVAGVLSDLATKTFGQPELSSDSGAGLATPTDSSGPEPEIIDDKSDNKSFSSMPPLEGEPGDPDLASDSWSTVSSSEDVVCC
4l2CON IALTESNVAGVLLDLAAKTFGQPEVSSDSGAGLATPTDSSGPEPEIIDDKSDNESFSSMPPLEGEPGDPDLTSDSWSTVSGSEDVVCC
4mCON VVLSESVISSALADLAAKSFGQSGVESDSGAGLSSPTEPSEPDPVIVDDRSDDESCSSMPPLEGEPGDPDLGSDSWSTVSGSEDVVCC
4m2CON VVLSESVISSALADLAAKSFGQSGVESDSGAGFTSPTEPSEPDPDTVDDRSDDGSYSSMPPLEGEPGDPDLGSDSWSTVSGSEDVVCC
4nCON VVLSESTVSSALADLAEKLFGPAPPDSDSGAGLTTPLDTSEPTPIIVDDISEDGSFSSMPPLEGEPGDPDLTADSWSTVSESEDVVCC
4oCON VILDESTVSNALADLAEKTFGRSEPDSDSGVAPTTATETSDPGPIIVDDQSDDGSYSSMPPLEGEPGDPDLTTDSWSTVSESEDVVCC
4pCON VALSESVISNALADLAAKSFGQPQAESDSGACLTTSTSTSDPAPIVVDDASEDGSYSSMPPLEGEPGDPDLTSDSWSTVSGSEDVVCC
4qCON VVLTESKVAEALADLATKTFGQAESSLDSGAGLTTSTETSEPDPIIADDRSDDGSYSSMPPLEGEPGDPDLTSDSWSTVSSSQDVVCC
4rCON VVLTESSVSDALAELAKKSFGRSEPDTDSGTGLTTPTDISQPGPPTTDGGTDGESYSSMPPLEGEPGDPDLTADSWSTVSSSDDVVCC
4sCON VALTESSVSEALAELAAKSFGQPAVDADSGTGLTSPTDASDPGPVIVDDNSDDGSYSSMPPLEGEPGDPDLTDGSWSTVSGSDDVVCC
4vCON VVLTESNVAQALSDLAETSFSRGPPSSDSGTGLTAPTESSEPDPIIVDDRSDDGSFSSMPPLEGEPGDPDLTSDSWSTVSGSEDVVCC
5aCON VELSDSTVSQVMADLADARFKADTIEGQDGSALGTSSQPDSGPEEKRDDDSDAASYSSMPPLEGEPGDPDLSSGSWSTVSSSDEVVCC
6aCON VHLDESTVSHALAQLADKVFVESSPGPSSDSGLSITSPVPPAPTTPDDACSEAESYSSMPPLEGEPGDPDLSSGSWSTVSDQDDVVCC
6bCON IQLDESAVSQALQQLADKVFVEDTSEPSSGLGGSIAGPSSPDPTTADDTCSDAGSFSSMPPLEGEPGDPDLSTGSWSTVSEEDDVVCC
6dCON VQLDEGSAKRALAELAQTSFPPSTLSEDSGRETSTLSSDMTPPREEADRASDDGSYSSMPPLEGEPGDPDLSSGSWSTVSEDHDVVCC
6eCON VQLDESAAARALAELARKAFPPDPSREDSGVGTSTTPTDASPVSDESDAHSDADSYSSMPPLEGEPGDPDLSSGSWSTVSEEGDVVCC
6e2CON VQLDESAAARALAELARKAFPPDPSREDSGVGTSTTPTDVSPVSDESDAHSDADSYSSMPPLEGEPGDPDLSSGSWSTVSEEGDVVCC
6fCON VLLDSSTVSQALAELAQKSFPEGSSRAESGIDCSVPSSDAVPLLGEDDAGSEAGSFSSMPPLEGEPGDPDLSSGSWSTVSDEDDVVCC
6gCON VQLDDSVVGHVLAQLAEKSFPATPPQTNSDSGHGTTNGAASLPSAEDDDASDADSYSSMPPLEGEPGDPDLSSGSWSTVSSEETVVCC
6hCON VQLDSSNVSAALAQLAAKTFETPSPTTGYGSDQPDHSTESSEHDRDDGVASEAESYSSMPPLEGEPGDPDLSSGSWSTVSEEGDVVCC
6iCON VQLDASNVSTVLAQLAETTFPQPALTTGYGSGQAAHSTGSGEQSEEDGAASEADSYSSMPPLEGEPGDPDLSSGSWSTVSEESDVICC
6kCON VHLDDSSVAEALAELAKKSFPTQPPSTDSDSGHPTTSKGSDPDVEKADAGSDAGSYSSMPPLEGEPGDPDLDSGSWSTVSEESDVVCC
6lCON VHLDDSTVSQALAQLAQKSFPTPSTATDLDSGCPVSSEGPDDSGEDAGAASDVESYSSMPPLEGEPGDPDLSSGSWSTVSEEGDVVCC
6mCON VQLDQSTVALALAQLAEKTFPASSPSTDQDSGCPNNDNGSEEVAGDAERLSEAESYSSMPPLEGEPGDPDLSSGSWSTVSEEEDIICC
6m2CON VQLDRSTVALALAQLAEKTFPASPPSTDQDSGCPNNDNGSEEAAGDAERLSEAESYSSMPPLEGEPGDPDLSSGSWSTVSEEEDIICC
6nCON IHLDQSTLPLALAQLAEKSFPTPSSSTDPDSGCPVNDSGSSEAKGDVETLSDAGSYSSMPPLEGEPGDPDLSSGSWSTVSEEGDVVCC
6oCON VRLDESSVSTALAELARKSFPPPSSQESSGLGSTAASSQSPESPNEADTASDAGSYSSMPPLEGEPGDPDLESGSWSTVSEESDVVCC
6pCON VQLDESSVSQALAELAQKSFPSPPSQGDSGAGPSVPTSIEAQSSDGVETASDAGSYSSMPPLEGEPGDPDLSSGSWSTVSEEGDVVCC
6rCON VQLDSSTVSQVLAELAQKSFPGSSSQEESGRGDSLPSSDAVLSPGEDDAGSEAGSFSSMPPLEGEPGDPDLSSGSWSTVSDEGDVVCC
6r2CON VQLDSSTVSQVLAELAQKSFPGSSSQEESGRGDSLPSSDAVPSPEEDDAGSEAGSFSSMPPLEGEPGDPDLSSGSWSTVSDEGDVVCC
6tCON VQLDDSAAAAALAELARKCFPSETSQGGSVTSPSAPSSDAASSQVEDDIESDAGSYSSMPPLEGEPGDPDLSSGSWSTVSEEGDVVCC
6uCON VQLDESTASAALAELARKLFPSGPEREESGMGTSAPSTSETPTSGEHDAQSDAGSYSSMPPLEGEPGDPDLSSGSWSTVSEDDDIVCC
6vCON IRLDDSTVTQALAEMAKKPFSHDSPDSGPDSGVPTTSTVASEPDASDGAASDVESYSSMPPLEGEPGDPDLSSGSWSTVSEEEDVVCC
6wCON IRLDESKVAQALAELASRTFQPSPAISKSDSGLGTSGVGESPDHDAEEETSDVDSCSSMPPLEGEPGDPDLSSGSWSTVSDDETVVCC
6w2CON IRLDESSVAQALAELASRTFQTTPAANNTDSGLGTSGAGESVDHDAEEEPSDADSYSSMPPLEGEPGDPDLSSGSWSTVSDDETVVCC

HCV NS5B

```
         1        10        20        30        40        50        60        70        80        90       100
1aCON    SMSYSWTGALVTPCAAEEQKLPINALSNSLLRHHNLVYSTTSRSACQRQKKVTFDRLQVLDSHYQDVLKEVKAAASKVKANLLSVEEACSLTPPHSAKSK
1bCON    SMSYTWTGALITPCAAEESKLPINALSNSLLRHHNMVYATTSRSASQRQKKVTFDRLQVLDDHYRDVLKEMKAKASTVKAKLLSVEEACKLTPPHSARSK
1cCON    SMSYSWTGALITPCAAEEAKLPINALSNSLLRHHNLVYSTTSRSAGQRQKKVTFDRLQVLDDHYRDVLKEAKAKASTVKAKLLSVEEACSLTPPHSARSK
1eCON    SMSYSWTGALVTPCAAEESKLPINALSNSLLRHHNMVYSTTSRSASQRQKKVTFDRVQVLDDHYKDVLKEVKAKASTVKADLLSVEDACALTPPHSARSK
1gCON    SMSYSWTGALVTPCAAEETKLPINALSNSLLRHHNMVYSTTSRSAGQRQKKVTFDRLQVLDSHYKDVVKEIKAKASTVKANLLSVEEACALTPPHSARSK
1hCON    SMSYSWTGALITPCAAEEEKLPINALSNTLLRHRNMVYATTSRSASQRQKKVTFDRMQVLDSHYQDVLKEAKAKASTVKARLLSVEEACRLTPPHSARSK
1lCON    SMSYTWTGALITPCAAEEASLPINALSNSLLRHRNLVYSTTSRSAGQRQKKVTFDRLQVLDDHYRDVLKEAKAKASTVKANMLSVEEACDLTPPHSAKSK
2aCON    SMSYSWTGALITPCSPEEEKLPINPLSNSLLRYHNKVYCTTSKSASLRAKKVTFDRMQVLDAHYDSVLKDIKLAASKVSARLLTLEEACQLTPPHSARSK
2bCON    SMSYSWTGALITPCGPEEEKLPINPLSNSLMRFHNKVYSTTSRSASLRAKKVTFDRVQVLDAHYDSVLQDVKRAASKVSARLLSVEEACALTPPHSAKSR
2cCON    SMSYSWTGALITPCSPEEEKLPINPLSNSLLRYHNKVYCTTSRSASQRAKKVTFDRVQLLDSHYDSVLKDVKQAASKVSAKLLSIEEACALTPPHSARSK
2fCON    SMSYSWTGALITPCSPEEEKLPINPLSNSLLRYHNKVYCTTSRSASQRAKKVTFDRVQLLDSYYDSTLKDIKLAASKVSAKLLSIEEACALTPPHSARSK
2iCON    SMSYSWTGALITPCSPEEEKLPINPLSNSLLRYHNKVYCTTSKSASLRAKKVTFDRVQLLDSHYDEVLKDVKLAASKVSARLLSVEEACALTPPHSARSK
2jCON    SMSYSWTGALITPCASEEEKLPINPLSNSLLRFHNKVYCTTSKSASLRAKKVTFDRVQLLDSYYDSTLKDIKLAASKVSARLLTIEEACALTPPHSARSK
2kCON    SMSYSWTGALITPCGPEEEKLPINPLSNSLLRYHNKVYSTTSRSASQRAKKVTFDRVQLLDSHYDQVLKDIKLAASKVSAKLLSIEEACALTPPHSARSK
2lCON    SMSYSWTGALITPCSPEGEKLPINPLSNSLLRYHNKVYSTTSKSAAARAKKVTFDRVQVLDSHYETVLQDVKQAASKVSANLLSLEEACGLTPPHSARSK
2mCON    SMSYSWTGALITPCSREEEKLPINPLSNSLLRYHNKVYCTTSRSASLRAKKVTFDRVQLLDSHYDSVLKDVKLAASKVSAQLLTVEEACSLTPPHSARSK
2m2CON   SMSYSWTGALITPCSREEEKLPINPLSNSLLRYHNKVYCTTSRSASLRAKKVTFDRVQLLDCHYDSALKDVKLAASKVSARLLTLEEACSLTPPHSARSK
3aCON    SMSYSWTGALITPCSAEEEKLPISPLSNSLLRHHNLVYSTSSRSASQRQKKVTFDRLQVLDDHYKTALKEVKERASRVKARMLTIEEACALVPPHSARSK
3bCON    SMSYSWTGAIITPCSAEEEKLPISPLSNSLLRHHNLVYSTSSRSAAARQKKVTFDRLQVLDDHYKNVLKEVKERASGVKGRLLSFEEACSLVPPHSGRSK
3b2CON   SMSYSWTGAIITPCSAEEEKLPISPLSNSLLRHHNLVYSTSSRSAAARQKKVTFDRLQVLDDHYKNVLKEVKERASGVKGRLLSFEEASSLVPPHSARSK
3b3CON   SMSYSWTGAIVTPCSAEEEKLPISPLSNSLLRHHNLVYSTSSRSAAARQKKVTFDRLQVLDDHYKNVLKEVKERASGVKGRLLSFEEACSLVPPHSARSK
3gCON    SMSYSWTGAIITPCSAEEEKLPISPLSNSLLRHHNLVYSTSSRSAALRQKKVTFDRMQVLDDHYKSTLKEVKERASGVKGRLLSFEEACALVPPHSAKSK
3g2CON   SMSYSWTGAIITPCSAEEEKLPISPLSNSLLRHHNLVYSTSSRSAAQRQKKVTFDRMQVLDDHYKSVLKEVKERAAGVKGRLLSFEEACALVPPHSARSK
3hCON    SMSYSWTGALITPCTAEEEKLPISPLSNSLLRHHNLVYSTSSRSAGLRQKKVTFDRLQVLDDHYKAVVKEIKEKASEVKADLLSVEEACRLTPPHSARSK
3iCON    SMSYSWTGAIITPCSAEEEKLPISPLSNSLLRHHNLVYSTSSRSAALRQKKVTFDRLQVLDDHYKSVLKEVKERASEVKGRLLTFEEACALVPPHSARSK
3kCON    SMSYSWTGALITPCAAEEEKLPISPLSNSLLRHHNLVYSTSSRSAAQRQKKVTFDRLQVLDDHYNTTLKEIKELASGVKAELLSVEEACRLVPSHSARSK
4aCON    SMSYSWTGALVTPCAAEESKLPISPLSNSLLRHHNMVYATTTRSAVTRQKKVTFDRLQVVDSHYNEVLKEIKARASRVKARLLTTEEACDLTPPHSARSK
4bCON    SMSYSWTGALVTPCAAEEPKLPISPLSNSLLRHHNMVYATTSRSATARQKKVTFDRLQVVDDHYHATLKEIKARASGVKAPALSVEEACSLTPPHSARSK
4cCON    SMSYSWTGALVTPCAAEESKLPISPLSNSLLRHHNMVYATTTRSATTRQKKVTFDRMQVVDNHYNEVLKEIKARASKVKARLLTVEEACDLTPPHSARSK
4dCON    SMSYSWTGALVTPCAAEETKLPINPLSNSLLRHHNMVYSTTSRSAVTRQKKVTFDRMQVVDNHYNDVLKEIKARASTVKARLLTVEEACSLTPPHSARSK
4fCON    SMSYSWTGALVTPSAAEESKLPINPLSNSLLRHHNMVYATTSKSATTRQKKVTFDRVQVLDNHYNETLKEIKARASRVKARTLSVEEACDLTPPHSARSK
4gCON    SMSYTWTGALVTPCAAEETKLPISPLSNSLLRHHNMVYATTSRSATTRQKKVTFDRLQVVDNHYHDVLKEIKARASGVKARTLSTEEACDLTPPHSAKSK
4g2CON   SMSYSWTGALVTPCAAEEAKLPINPLSNSLLRHHNMVYATTSRSATTRQKKVTFDRLQVVDAHYHEVLKEIKARASGIKARTLSTEEACDLTPPHSARSK
4kCON    SMSYSWTGALITPCAAEETKLPISPLSNSLLRHHNMVYATTSRSATARQKKVTFDRLQVLDNHYHDVLKEIKARATRVKARLLSTEEACDLTPPHSAKSK
4k2CON   SMSYSWTGALITPCAAEETKLPISPLSNSLLRHHNMVYATTSRSATARQKKVTFDRLQVLDNHYHDVLKEIKARATRVKARLLSTEEACDLTPPHSAKSK
4lCON    SMSYSWTGALVTPCAAEESKLPISPLSNSLLRHHNMVYATTSRSAVTRQKKVTFDRLQMVDDHYNAVLKEIKARASSVKARVLSVEEACDLTPPHSARSK
4l2CON   SMSYSWTGALVTPCAAEETKLPISPLSNSLLRHHNMVYATTSRSAVTRQKKVTFDRLQVVDDHYNAVLKEIKARASTVKARVLSVEEACDLTPPHSAKSK
4mCON    SMSYSWTGALVTPCAAEETKLPISPLSNSLLRHHNMVYATTSRSAVTRQKKVTFDRMQVVDSHYNDTLKEIKARASGVKARALSVEEACDLTPPHSARSK
4m2CON   SMSYSWTGALVTPCAAEETKLPISPLSNSLLRHHNMVYATTSRSAATRQKKVTFDRMQVIDSHYNDTLKEIKARASGVKARTLSVEEACDLTPPHSAKSK
4oCON    SMSYSWTGALVTPCAAEESKLPISPLSNSLLRHHNMVYATTSRSAVTRQKKVTFDRMQVVDSHYYDVLKEIKAKASTVKARLLTTEEACSLTPPHSARSK
4o2CON   SMSYSWTGALVTPCAAEESKLPISPLSNSLLRHHNMVYATTSRSAVIRQKKVTFDRMQVVDSHYNEVLKEIKAKASTVKARVLTTEEACSLTPPHSARSK
4pCON    SMSYTWTGALVTPCAAEESKLPISPLSNSLLRHHNMVYATTSRSAVARQKKVTFDRMQVVDSHYNAVLKEIKARASKVKARTLSVEEACDLTPPHSARSK
4qCON    SMSYSWTGALVTPCAAEESKLPISPLSNSLMRHHNMVYATTSRSAVTRQKKVTFDRLQVVDQHYYDVLKEIKARASTVQARTLSVEEACDLTPPHSARSK
4rCON    SMSYSWTGALVTPCAAEEAKLPINPLSNSLLRHHNLVYATTSRSATARQKKVTFDRLQVVDSHYHAVLKEIKQRASGVKARTLSTEEACALTPPHSARSK
4r2CON   SMSYSWTGALVTPCAAEENKLPINPLSNSLLRHHNLVYATTSRSATARQKKVTFDRLQVVDDHYKAVLKEIKHRASGVKARTLSTEEACALTPPHSARSK
4sCON    SMSYSWTGALVTPCAAEETKLPISPLSNSLLRHHNMVYSTTSRSAAIRQKKVTFDRLQVVDSHYNEVLKEIKARASKVKARVLSTEEACDLTPPHSARSK
4vCON    SMSYSWTGALVTPCAAEESKLPISPLSNSLMRHHNMVYSTTSRSAVTRQKKVTFDRMQVVDSHYNDVLKEIKARASTVKARALSVEEACDLTPPHSARSK
5aCON    SMSYTWTGALITPCSAEEEKLPINPLSNTLLRHHNLVYSTSSRSAGLRQKKVTFDRLQVLDDHYREVVDEMKRLASKVKARLLPLEEACGLTPPHSARSK
6aCON    SMSYSWTGALITPCAAEEEKLPINPLSNSLIRHHNMVYSTTSRSASLRQKKVTFDRVQVFDQHYQDVLKEIKLRASTVQAKLLSIEEACDLTPSHSARSK
6bCON    SMSYTWTGALITPCAAEEEKLPINPLSNSLIRHHNMVYSTTSRSAGLRQKKVTFDRLQVVDQHYQDVLKEIKLRASTVHARLLSTEEACSLTPPHSARSR
6dCON    SMSYSWTGALITPCAAEEEKLPISPLSNALIRHHNLVYSTTSRSASLRQKKVTFDRVQVVDQHYYDVLKEIKTKASGVSAKLLSVEEACALTPPHSARSK
6eCON    SMSYSWTGALITPCAAEEEKLPISPLSNSLIRHHNMVYSTTTRSAALRQKKVTFDRVQVLDQHYQDILKEVKDRASSVSARLLSTEEACKLTPPHSARSK
6fCON    SMSYSWTGALITPCAAEEEKLPISPLSNSLIRHHNMVYSTTSRSAPLRQKKVTFDRIQILDQHYSDVLKEVKARASQVSARLLSTEEACDLTPAHSARSK
6gCON    SMSYSWTGALITPCAAEEEKLPISPLSNTLIRHHNMVYSTTSRSAALRQKKVTFDRQQVVDQHYYDTLKEMKARASTVSAKLLSVEEACDLTPAHSARSK
6hCON    SYSYSWTGALVTPCAAEEEKLPINPLSNSLIRHHNLVYSTSSRSAATRQKKVTFDRVQLLDQHYYDTVKEIKLRASHVKAQLLSTEEACDLTPPHSARSK
6iCON    SYSYSWTGALITPCAAEEEKLPINPLSNSLIRHHNLVYSTSSKSACLRQKKVTFDRVQLVDQHYHDTLKEIKLQASQVQARLLTTEEACDLTPPHSARSK
6kCON    SMSYSWTGALVTPCAAEEEKLPINPLSNSLIRHHNLVYSTTTRSAAMRQKKVTFDRLQILDQHYNNVVKEVKLRASGVTAKLLSVEEACSLTPPHSARSK
6nCON    SMSYSWTGAIVTPCAPEEEKLPINPLSNSLIRHHNLVYSTTSRSAAMRQKKVTFDRMQVLDKHYDDVLKEVKLRASQVTAKLLSIEDACDLTPPHSARSK
6oCON    SYSYSWTGALVTPCAAEEEKLPISPLSNSLIRHHNLVYSTTSRSAAIRQKKVTFDRIQLLDQHYHDTLKEIKERASTVSARLLTTEEACDLTPPCSARSK
6rCON    SMSYSWTGALVTPCAAEEEKLPISPLSNSLIRHHNLVYSTTSRSASLRQKKVTFDRVQVVDQHYHDVLKEIKARASKVSARLLSTEEACDLTPAHSARSK
6uCON    SYSYSWTGALITACAAEEEKLPISPLSNSLIRHHNLVYSTTSRSAALRQKKVTFDRLQVVDQYYHDTLKEIKLRASKVSAGLLSVEEACDLTPPHSARSK
6vCON    SYSYSWTGNVITPCVPEEEKLPINPLSNSLLRHHNLVYSTTTRSASVRQKKVTFDRLQVLDQHYHDTLKEIKLRASTVTARLLSVEEACDLTPPHSARSK
6wCON    SMSYSWTGAPITPCAAEEEKLPISPLSNGLLRHHNLVYSTTSRSAPLRQKKVTFDRLQVLDSYYYDTVKEIKTLASGVKAKLLSIEEACDLTPPRSARSK
6w2CON   SMSYSWTGAPITPCAAEEEKLPISPLSNGLLRHHNLVYSTTSRSAPLRQKKVTFDRMQVLDSFYHDVVKEVKTLASGVKARLLSTEEACDLTPPRSARSK
```

```
                 110       120       130       140       150       160       170       180       190       200
1aCON    FGYGAKDVRCHARKAVNHINSVWKDLLEDSVTPIDTTIMAKNEVFCVQPEKGGRKPARLIVFPDLGVRVCEKMALYDVVSKLPLAVMGSSYGFQYSPGQR
1bCON    FGYGAKDVRNLSSKAVNHIRSVWKDLLEDTETPIDTTIMAKNEVFCVQPEKGGRKPARLIVFPDLGVRVCEKMALYDVVSTLPQAVMGSSYGFQYSPGQR
1cCON    FGYGAKDVRSHSSKAIRHINSVWQDLLEDNTTPIDTTIMAKNEVFCVKPEKGGRKPARLIVYPDLGVRVCEKRALYDVVKQLPIAVMGASYGFQYSPAQR
1eCON    FGYGAKDVRSLASKAVRHINSVWQDLLEDNETPIDTTIMAKNEVFCVQPEKGGRKPARLIVFPDLGVRVCEKRALYDITKKLPIAVMGSSYGFQYSPAQR
1gCON    FGYGAKDVRSLSSKAVRHINSVWQDLLEDNQTPIDTTIMAKNEVFCVQPEKGGRKPARLIVFPDLGVRVCEKRALYDVVKKLPLAVMGGSYGFQYSPAQR
1hCON    FGYGAKDVRDLTSKAVRHINSVWQDLLEDNHTPIDTTIMAKNEVFCVDPSKGGRKPARLIVFPDLGVRVCEKMALYDVISKLPQAVMGESYGFQYSPGQR
1lCON    FGYGAKDVRSHAPKAVRHIKSVWQDLLEDNETPLDTTIMAKNEVFCVAPEKGGKKPARLIVYPDLGVRVCEKMALYDVVKKLPIAVMGDAYGFQYSPSQR
2aCON    YGFGAKEVRSLSGRAVNHIKSVWKDLLEDSQTPIPTTIMAKNEVFCVDPTKGGKKAARLIVYPDLGVRVCEKMALYDVTQKLPQAVMGASYGFQYSPAQR
2bCON    YGFGAKEVRSLSRRAVNHIRSVWEDLLEDQHTPIDTTIMAKNEVFCVDPTKGGKKPARLIVYPDLGVRVCEKMALYDIAQKLPKAIMGPSYGFQYSPAER
2cCON    YGFGAKEVRSLSRRAVNHIKSVWEDLLEDHCSPIDTTIMAKNEVFCVDPAKGGKKPARLIVYPDLGVRVCEKMALYDITQKLPVAVMGRSYGFQYSPAQR
2fCON    YGFGAKEVRSLSRRAVNHIKSVWEDLLEDQETPIPTTIMAKNEVFCVNPAKGGKKAARLIVYPDLGVRVCEKMALYDITQKLPIAVMGASYGFQYSPAER
2iCON    YGFGAKEVRSLSRRAVDHIKSVWKDLLEDPQTPIQTTIMAKNEVFCVDPAKGGKKAARLIVYPDLGVRVCEKMALYDIAQKLPQAVMGQSYGFQYSPAQR
2jCON    YGFGAKEVRSLAKRAVNHIKSVWKDLLEDSDTPIPTTIMAKNEVFCVDPAKGGKKAARLIVYPDLGVRVCEKIALYDITQKLPQAVMGASYGFQYSPAQR
2kCON    YGFGAKEVRSLSRKAVNHIKSVWKDLLEDQQTPIPTTIMAKNEVFCVDPTKGGKKAARLIVYPDLGVRVCEKMALYDITQKLPQAVMGASYGFQYSPAQR
2lCON    FGYGAKDVRGLSRRAVNHIKSVWEDLLEDSSTPISTTIMAKNEVFCVDPAKGGKKPARLIVFPDLGVRVCEKRALYDIAQKLPQAIMGASYGFQYSPSQR
2mCON    YGYGAKEVRSLSKRAVDHIRSVWKDLLEDSQTPIDTTIMAKNEVFCVDPAKGGKKAARLIVFPDLSVRVCEKMALYDITQKLPQAVMGRSYGFQYSPAQR
2m2CON   YGYGAKEVRSLSKRAVNHIRSVWEDLLEDQQTPINTTIMAKNEVFCVDPAKGGKKAARLIVFPDLSVRVCEKMALYDITQKLPQAVMGSSYGFQYSPAQR
3aCON    FGYSAKDVRSLSSKAINQIRSVWEDLLEDTTTPIPTTIMAKNEVFCVDPVKGGRKPARLIVYPDLGVRVCEKRALYDVIQKLSIETMGSAYGFQYSPQQR
3bCON    YGYSAKDVRSLSSKAMNQIRSVWEDLLEDNSTPIPTTIMAKNEVFSVNPAKGGRKPARLIVYPDLGVRVCEKRALYDVIQKLSIATMGPAYGFQYSPKQR
3b2CON   YGYSAKDVRSLSSKAMNQIRSVWEDLLEDNATPIPTTIMAKNEVFSVNPAKGGRKPARLIVYPDLGVRVCEKRALYDVLQKLSITTMGSAYGFQYSPKQR
3b3CON   YGYSARDVRSLSSKAMNQIRSVWEDLLEDNTTPISTTIMAKNEVFSVNPAKGGRKPARLIVYPDLGVRVCEKRALYDVIQKLSTATMGAAYGFQYSPSQR
3gCON    HGYSAKDVRSLSSKAMNQIRSVWEDLLEDNSTPIPTTIMAKNEVFSVNPSKGGRKPARLIVYPDLGVRVCEKRALYDVIQQLSLAIMGPAYGFQYSPKQR
3g2CON   YGYSAKDVRSLSSKAINQIHSVWEDLLEDNITPIPTTIMAKNEVFSVNPSKGGRKPARLIVYPDLGVRVCEKRALYDVIQQLSLAVMGSAYGFQYSPKQR
3hCON    YGYGAKEVRSLAGKAVNHIHSVWEDLLADSTTPIPTTIMAKNEVFAVNPSKGGRKPARLIVFPDLSVRVCEKRALYDVTQKLPTAVMGPAYGFQYSPAQR
3iCON    YGYSAKDVRSLASKAVNQIRSVWEDLLEDTTTPIPTTIMAKNEVFSVNPAKGGRKPARLIVYPDLGVRVCEKRALYDVIQKLSTATMGAAYGFQYSPKQR
3kCON    FGYGAKEVRSLSSKAINHINSVWEDLLEDNTTPIPTTIMAKNEVFAVAPHKGGRKPARLIVYPDLGVRICEKRALYDVIQKLPSAIMGSAYGFQYSPKQR
4aCON    FGYGAKDVRSHSRKAINHISSVWKDLLDDNNTPIPTTIMAKNEVFAVNPAKGGRKPARLIVYPDLGVRVCEKRALHDVIKKLPEAVMGAAYGFQYSPAQR
4bCON    FGYGAKDVRSLSRKAIDHINSVWEDLREDSVTPIATTIMAKNEVFSVKPEKGGRKPARLIVFPDLGVRVCEKRALYNVIQKLPQAIMGEAYGFQYSPAQR
4cCON    FGYGAKDVRSHSRKAVNHINSVWEDLLEDNTTPIPTTIMAKNEVFAVKPEKGGRKPARLIVYPDLGVRVCEKRALHDAVKQLPEAVMGAAYGFQYSPAQR
4dCON    FGYGAKEVRSHARKAINHINSVWEDLREDTTTPIPTTIMAKNEVFSVKPEKGGRKPARLIVYPDLGVRVCEKRALYDAVKQLSLAVMGDSYGFQYSPSQR
4fCON    FGYGAKDVRSHSRKAVNHINSVWEDLLEDNTTPIPTTIMAKNEVFSVNPQKGGRKPARLIVYPDLGVRVCEKMALHDAVKKLSTAVMGDAYGFQYSPAQR
4gCON    FGYGAKDVRSHSRKAINHINSVWEDLLEDSVTPIPTTIMAKNEVFSVKPEKGGRKPARLIVYPDLGVRVCEKKALYDVIQQLPKAVMGEAYGFQYSPAQR
4g2CON   FGYGAKDVRSHSRKAINHINSVWEDLLEDSVTPIPTTIMAKNEVFSVKPEKGGRKPARLIVYPDLGVRVCEKKALYNVIQELPKAIMGEAYGFQYSPAQR
4kCON    FGYGAKDVRSHSRKAINHINSVWEDLLEDTATPIPTTIMAKNEVFSVKPEKGGRKPARLIVYPDLGVRVCEKKALHDAIQQLPKAVMGDAYGFQYSPAQR
4k2CON   FGYGAKDVRSHSRKAINHINSVWEDLLEDTATPIPTTIMAKNEVFSVKPEKGGRKPARLIVYPDLGVRVCEKKALHDAIQQLPKAVMGDAYGFQYSPAQR
4lCON    FGFGAKDVRGHSRRAINHINSVWEDLLEDNCTPIPTTIMAKNEVFSVKPEKGGRKPARLIVYPDLGVRVCEKRALYDVVQRLPEAVMGAAYGFQYSPAQR
4l2CON   FGFGAKDVRSHSRRAINHINSVWEDLLEDNNTPIPTTIMAKNEVFSVKSEKGGRKPARLIVYPDLGVRVCEKRALHDVVKKLPEAVMGAAYGFQYSPAQR
4mCON    FGYGAKDVRSHARKAVNHINSVWEDLLGDNITPIPTTIMAKNEVFSVKPEKGGRKPARLIVYPDLGVRVCEKRALYDVVQKLPKAVMGAAYGFQYSPQQR
4m2CON   FGYGAKDVRSHSRKAVNHINSVWEDLLGDNNTPISTTIMAKNEVFSVKPEKGGRKPARLIVYPDLGVRVCEKRALYDAIQKLPKAVMGAAYGFQYSPQQR
4oCON    FGYGAKDVRSHSRKAINHINSVWEDLLEDTTTPIPTTIMAKNEVFSVKPEKGGRKPARLIVYPDLGVRVCEKRALYDALQKLPGAVMGAAYGFQYSPAQR
4o2CON   FGYGAKDVRSHSRKAVNHINSVWEDLLEDQVTPIPTTIMAKNEVFSVKPEKGGRKPARLIVYPDLGVRVCEKKALYDALQKLPGAVMGAAYGFQYSPAQR
4pCON    FGYGAKDVRSHSRKAINHINSVWEDLLEDNTTPIPTTIMAKSEVFSVKPEKGGRKPARLIVYPDLGVRVCEKRALHDAVKKLAEATMGASYGFQYSPAQR
4qCON    FGYGAKDVRSHSRKAINHINSVWEDLLGDNNTPIPTTIMAKNEVFSVKPEKGGRKPARLIVYPDLGVRVCEKRALYDVVQKLPQAVMGAAYGFQYSPAQR
4rCON    FGYGAKDVRSLSRKAVNHINSVWEDLLEDSATPIPTTIMAKNEVFAVRPEKGGRKPARLIVYPDLGVRVCEKKALYNVIQELPKAVMGDAYGFQYSPAQR
4r2CON   FGYGAKDVRSLSRKAVNHINSVWEDLLEDSVTPIPTTIMAKNEVFAVRPEKGGRKPARLIVFPDLGVRVCEKKALYNVIQTLPKAVMGDAYGFQYSPAQR
4sCON    FGYGAKDVRSHTRKAINHIDSVWEDLREDNNTPIPTTIMAKNEVFSVKPEKGGRKSARLIVYPDLSVRVCEKRALYDVVKQLPEAVMGAAYGFQYSPLQR
4vCON    FGYGAKDVRSHSRKAINHINSVWEDLLEDNNTPIPTTIMAKNEVFSVKPEKGGRKPARLIVYPDLGVRVCEKRALYDAVQQLPEAVMGASYGFQYSPAQR
5aCON    YGYGAKEVRSLDKKALKHIEGVWQDLLDDSDTPLPTTIMAKNEVFAVEPSKGGKKPARLIVYPDLGVRVCEKRALYDVAQKLPTALMGPSYGFQYSPAQR
6aCON    YGYGAQDVRSHASKAVDHIRSVWEDLLEDSDTPIPTTIMAKNEVFCVDPSKGGRKPARLIVYPDLGVRVCEKMALYDVTRKLPQAVMGSAYGFQYSPNQR
6bCON    YGYGARDVRSHTSKAVKHIDSVWEDLLEDNATPIPTTIMAKNEVFCVDPSKGGRKPARLIVYPDLSVRVCEKMALYDVTQKLPKTVMGSAYGFQYSPSQR
6dCON    FGYGAKEVRGLASKAVNHINSVWEDLLEDNSTPIPTTIMAKNEVFCVDAQKGGRKPARLIVYPDLGVRVCEKRALYDVTQKLPIAVMGAAYGFQYSPKQR
6eCON    FGYGAKEVRSLASKAINHINSVWEDLLEDNSTPIPTTIMAKNEVFCVDARKGGRKPARLIVYPDLGVRVCEKRALYDITCKLPVAVMGAAYGFQYSPKQR
6fCON    FGYGAKEVRAHASKAINHINSVWEDLLEDNSTPIPTTIMAKNEVFCVDPQKGGRKPARLIVYPDLGVRVCEKRALYDVTRKLPLAVMGPAYGFQYSPKQR
6gCON    FGYGAKDVRGRTSKALNHINSVWEDLLEDNVTPIPTTIMAKNEVFCVDVSKGGRKPARLIVYPDLSVRVCEKRALYDVTRKLPVAVMGAAYGFQYSPSQR
6hCON    FGYGAKDVRSHASKAINHINSVWADLLEDTQTPIPTTIMAKNEVFCVDASKGGRKSARLIVYPDLGVRVCEKRALFDVTRKLPTAIMGDAYGFQYSPQQR
6iCON    FGYGAKDVRGHTRKAIDHINSVWEDLLEDTQTPIPTTIMAKNEVFCVDTSKGGRKPARLIVYPDLGVRVCEKKALFDITRKLPVAIMGDAYGFQYSPKQR
6kCON    FGYGAKDVRSHTSKAINHINSVWEDLLEDNQTPIPTTIMAKNEVFCADVSKGGRKPARLIVYPDLGVRVCEKRALYDVTRKLPTAIMGDAYGFQYSPKQR
6nCON    FGYGAKDVRSHTSKAINHISSVWEDLLEDNQTPIPTTIMAKNEVFCVDPSRGGRKPARLIVYPDLGVRVCEKRALFDITQKLPLATMGDAYGFQYSPKQR
6oCON    FGYGAKEVRSHASKAINHINSVWEDLLEDNATPIPTTIMAKNEVFCVDAKKGGRKPARLIVYPDLGVRVCEKKALYDITRKLPQAVMGAAYGFQYSPKQR
6rCON    FGYGAKEVRAHASKAINHINSVWEDLLEDNSTPIPTTIMAKNEVFCVDAQKGGRKPARLIVFPDLGVRVCEKRALYDVTRKLPVAVMGPSYGFQYSPKQR
6uCON    FGYGAKDVRSHASKAVNHINSVWKDLLEDKTTPIPTTIMAKNEVFCVEPSKGGRKPARLIVYPDLGVRVCEKIALYDITRKLPTAVMGAAYGFQYSPKDR
6vCON    FGYGAKDVRSHSSKAINHINSVWEDLLEDTSTPIPTTIMAKNEVFCVNPAKGGRKPARLIVFPDLGVRVCEKRALYDISQKLPQAIMGGAYGFQYSPRDR
6wCON    FGYGAKDVRAHASKAVHHISSVWEDLLEDSSTPIPTTVMAKNEVFCVDSSKGGRKPARLIVYPDLSVRVCEKRALYDITQKLPAAIMGAAYGFQYSPNQR
6w2CON   FGYGAKDVRAHASKAVDHISSVWEDLLEDNSTPIPTTVMAKNEVFCVDSSKGGRKPARLIVYPDLSVRVCEKRALYDITQKLPVAIMGAAYGFQYSPNQR
```

```
                 210       220       230       240       250       260       270       280       290       300
1aCON     VEFLVQAWKSKKTPMGFSYDTRCFDSTVTESDIRTEEAIYQCCDLDPQARVAIKSLTERLYVGGPLTNSRGENCGYRRCRASGVLTTSCGNTLTCYIKAQ
1bCON     VEFLVNAWKSKKCPMGFAYDTRCFDSTVTESDIRVEESIYQCCDLAPEARQAIRSLTERLYIGGPLTNSKGQNCGYRRCRASGVLTTSCGNTLTCYLKAS
1cCON     VDFLLKAWKSKKVPMGFSYDTRCFDSTVTEADIRTEEDLYQSCDLAPEARIAIRSLTERLYIGGPLTNSKGQNCGYRRCRASGVLTTSCGNTITCYLKAS
1eCON     VKFLVDAWKSKKCPMGFSYDTRCFDSTVTEADIRTEESIYQCCDLHPEARVAIKSLTERLYVGGPLTNSKGENCGYRRCRASGVLTTSCGNTLTCYIKAL
1gCON     VEFLVKAWKSKKTPMGFSYDTRCFDSTVTEADIRTEESIYQCCDLHPDARVAIKNLTERLYVGGPLTNSKGENCGYRRCRASGVLTTSCGNTLTCYIKAL
1hCON     VEFLVKTWKSKANPMGFSYDTRCFDSTVTEHDIRVEESIYQSCDLDPDARKAIASLTERLYIGGPLTNSRGENCGYRRCRASGVLTTSCGNTLTCYLKAM
1lCON     VEFLVKTWKSKKTPMGFSYDTRCFDSTVTEADIRTEESLYQCCDLAPEARVAIKSLTERLYVGGPLTNSKGENCGYRRCRASGVLTTSCGNTLTCYLKAT
2aCON     VEFLLKAWAEKKDPMGFSYDTRCFDSTVTERDIRTEESIYQACSLPEEARTAIHSLTERLYVGGPMFNSKGQTCGYRRCRASGVLTTSMGNTITCYVKAL
2bCON     VDFLLKAWGSKKDPMGFSYDTRCFDSTVTERDIRTEESIYQACSLPQEARTVIHSLTERLYVGGPMTNSKGQSCGYRRCRASGVFTTSMGNTMTCYIKAL
2cCON     VDFLLQAWKEKKTPMGFSYDTRCFDSTVTERDIRTEESIYLSCSLPEEARTAIHSLTERLYVGGPMTNSKGQSCGYRRCRASGVLTTSMGNTLTCYVKAR
2fCON     VDFLLRAWKEKKDPMGFSYDTRCFDSTVTERDIRTEEAIYLSCSLPEQARLAIHSLTERLYVGGPMMNSKGQSCGYRRCRASGVLTTSMGNTITCYLKAS
2iCON     VDFLLRAWKEKRDPMGFSYDTRCFDSTVTERDIRVEESIYLSCSLPEEARTAIHSLTERLYVGGPMQNSKGQSCGYRRCRASGVLTTSMGNTLTCYVKAQ
2jCON     VEFLLKAWKEKKSPMGFSYDTRCFDSTVTERDIRTEESIYLACSLPEEARKAIHSLTERLYVGGPMLNSKGQSCGYRRCRASGVLTTSMGNTITCYVKAL
2kCON     VDFLLRAWKEKKDPMGFSYDTRCFDSTVTERDIRTEESIYLACSLPEEARVAIHSLTERLYVGGPMTNSKGQSCGYRRCRASGVLTTSMGNTITCYVKAL
2lCON     VDFLLNAWRSKKTPMGFSYDTRCFDSTVTERDIRTEESIYLACQLPEEARKAIKSLTERLYVGGPMENSKGQACGYRRCRASGVFTTSLGNTMTCYIKAK
2mCON     VEFLLQAWKEKKDPMGLSYDTRCFDSTVTERDIRVEESIYQACSLPEEARAAIHSLTERLYVGGPMSNSKGQSCGYRRCRASGVLTTSMGNTLTCYVKAQ
2m2CON    VEYLLQAWKGKKDPMGFSYDTRCFDSTVTERDIRVEESIYQACSLPEEARAAIHSLTERLYVGGPMSNSKGQSCGYRRCRASGVLTTSMGNTLTCYVKAQ
3aCON     VERLLKMWTSKKTPLGFSYDTRCFDSTVTEQDIRVEEEIYQCCNLEPEARKVISSLTERLYCGGPMFNSKGAQCGYRRCRASGVLPTSFGNTITCYIKAT
3bCON     VEHLLKMWTSKKTPLGFSYDTRCFDSTVTEHDIRTEEGIYQCCDLEPEARKAISALTERLYIGGPMYNSKGLQCGYRRCRASGVLPTSFGNTITCYIKAT
3b2CON    VEHLLKMWTSKKTPMGFSYDTRCFDSTVTEHDIRTEEEIYQCCDLEPEARKAISALTERLYIGGPMYNSKGIQCGYRRCRASGVLPTSFGNTITCHIKAT
3b3CON    VEHLLKMWSSKKTPFGFSYDTRCFDSTVTEHDIRMEEEIYQCCDLEPEARKAISALTERLYIGGPMYNSKGLQCGYRRCRASGVLPTSFGNTITCYIKAT
3gCON     VEHLLKMWTSKKTPLGFSYDTRCFDSTVTEQDIRVEEEIYQCCDLEPEARKAISSLTERLYVGGPMYNSKGLKCGYRRCRASGVLPTSFGNTVTCYIKAT
3g2CON    VEHLLKMWASKKTPLGFSYDTRCFDSTVTEQDIRVEEEIYQCCDLEPEARKAISSLTERLYIGGPMYNSKGLKCGYRRCRASGVLPTSFGNTLTCYIKAT
3hCON     VERLLDMWKSKKVPLGFSYDTRCFDSTVTEQDIRVEESIYQSCQLTPEAKVAIKALTERLYIGGPMFNSKGEKCGYRRCRASGVLPTSLGNTITCYIKAR
3iCON     VEQLLKMWTAKKTPLGFSYDTRCFDSTVTEQDIRVEEEIYQCCDLEPEARQAIKALTERLYVGGPMFNSKGLKCGYRRCRASGVLPTSFGNTITCYIKAK
3kCON     VEYLLKMWNSKKTPLGFSYDTRCFDSTVTEQDIRVEESIYQACDLKDEARRVITSLTERLYCGGPMFNSKGQHCGYRRCRASGVLPTSFGNTVTCYLKAK
4aCON     VEFLLTAWKSKKTPMGFSYDTRCFDSTVTEKDIRVEEEVYQCCDLEPEARKVITALTDRLYVGGPMHNSKGDLCGYRRCRASGVYTTSFGNTLTCYLKAT
4bCON     VEYLLKSWNSKKVPMGFSYDTRCFDSTVTERDIQVEEEIYQCCDLDPETRKVISSLTERLYKGGPMYNSKGDLCGYRRCRASGVLTTSFGNTLTCYLKAT
4cCON     VEYLLSSWKAKKTPMGFSYDTRCFDSTVTEKDIRVEEEVYQCCDLEPEARKAITALTERLYVGGPMHNSKGDLCGYRRCRASGVYTTSFGNTLTCYLKAS
4dCON     VEFLLNAWRSKKTPMGFSYDTRCFDSTVTEKDIRVEEEVYQCCDLEPEARKVISALTERLYVGGPMYNSKGDLCGIRRCRASGVFTTSFGNTLTCYLKAS
4fCON     VEYLLKAWGSKKVPMGFSYDTRCFDSTVTERDIRVEEEVYQCCDLEPETRKAISALTERLYVGGPMYNSRGDLCGYRRCRASGVYTTSFGNTLTCFLKAT
4gCON     VDFLLKSWNSKRVPMGFSYDTRCFDSTVTERDIRVEEEIYQCCDLEPDARKVISALTERLYVGGPMFNSRGDLCGQRRCRASGVYTTSFGNTVTCYLKAV
4g2CON    VEYLLKAWRAKKTPMGFSYDTRCFDSTVTERDIRTEEEIYQCCDLEPEARKVISALTERLYVGGPMYNSKGDLCGQRRCRASGVYTTSFGNTVTCYLKAV
4kCON     VDYLLKSWKAKKVPMGFSYDTRCFDSTVTERDIRVEEEIYQCCDLEPDARKAISALTERLYLGGPMYNSKGDLCGYRRCRASGVYTTSFGNTVTCYLKAV
4k2CON    VDYLLKSWKAKKVPMGFSYDTRCFDSTVTERDIRVEEEIYQCCDLEPDARKAISALTERLYLGGPMYNSKGDLCGYRRCRASGVYTTSFGNTVTCYLKAV
4lCON     VDYLLTAWRQKKSPMGFSYDTRCFDSTVTEKDIRVEEEVYQCCDLEPEARKVITALTERLYVGGPMYNSKGDLCGVRRCRASGVFTTSFGNTLTCYLKAT
4l2CON    VEYLLTAWKQKKSPMGFSYDTRCFDSTVTEKDIRVEEEVYQCCDLEPEARKVITALTERLYVGGPMYNSKGDLCGVRRCRASGVFTTSFGNTLTCYLKAM
4mCON     VEYLLEAWRSKKTPMGFSYDTRCFDSTVTEKDIRVEEEVYQCCDLEPETRKVITALTERLYVGGPMYNSRGDLCGYRRCRASGVYTTSFGNTLTCYLKAT
4m2CON    VEYLLDAWRSKKTPMGFSYDTRCFDSTVTEKDIRTEEEVYQCCDLDPEARKVITALTERLYVGGPMYNSKGDLCGYRRCRASGVYTTSFGNTLTCYLKAT
4oCON     VEYLLAAWRSKKVPMGFSYDTRCFDSTVTEKDIGVEEEVYQCCNLEPEARKVISALTERLYKGGPMYNSKGDLCGQRRCRASGVFTTSFGNTLTCYLKAT
4o2CON    VEYLLAAWRSKKVPVGFSYDTRCFDSTVTEKDIGVEEEVYQCCDLEPEARKVISALTERLYKGGPMYNSKGDLCGQRRCRASGVFTTSFGNTLTCYLKAT
4pCON     VEYLLNAWKQKKVPMGFSYDTRCFDSTVTEKDIRVEEEVYQCCDLEPDTRKVITALTERLYVGGPMYNSKGDLCGVRRCRASGVFTTSFGNTLTCYLKAT
4qCON     VEYLLAAWRSKKTPMGFSYDTRCFDSTVTEKDIRVEEEVYQCCNLDPETRKVITALTERLYVGGPMYNSKGDLCGVRRCRASGVFTTSFGNTLTCYLKAT
4rCON     VDYLLKSWNSKRVPMGFSYDTRCFDSTVTERDIRVEEEIYQCCDLDADARKAITALTERLYVGGPMHNSKGDLCGYRRCRASGVYTTSFGNTVTCYLKAT
4r2CON    VEYLLKSWNSKRVPMGFSYDTRCFDSTVTERDIRVEEEIYQCCDLDADARKAITALTERLYVGGPMHNSKGDLCGYRRCRASGVYTTSFGNTVTCYLKAT
4sCON     VEYLLDAWKSKKTPMGFSYDTRCFDSTVTEKDIRVEEEVYQCCNLEPEARKVITALTERLYVGGPMFNSRGDLCGYRRCRASGVFTTSFGNTLTCYLKAS
4vCON     VEYLLAAWRSKKSPMGFSYDTRCFDSTVTEKDIRTEGEVYQCCDLEPEARKVITALTERLYVGGPMYNSKGDLCGVRRCRASGVFTTSFGNTLTCYLKAT
5aCON     VDFLLKAWKSKKIPMAFSYDTRCFDSTITEHDIMTEESIYQSCDLQPEARVAIRSLTQRLYCGGPMYNSKGQQCGYRRCRASGVFTTSMGNTMTCYIKAL
6aCON     VEYLLKMWRSKKVPMGFSYDTRCFDSTVTERDIRTENDIYQSCQLDPVARRAVSSLTERLYVGGPMVNSKGQSCGYRRCRASGVLPTSMGNTLTCYLKAQ
6bCON     VEYLLKMWRSKKTPMGFSYDTRCFDSTVTERDIRTEEDIYQSCQLDPTARKAISSLTERLYCGGPMFNSKGESCGYRRCRASGVLTTSLGNTLTCYLKAQ
6dCON     VDYLLKMWRSKKTPMGFSYDTRCFDSTVTERDIRTEEDIYQCCQLDPVAKKAITSLTERLYCGGPMYNSRGQSCGYRRCRASGVLTTSLGNTLTCYLKAQ
6eCON     VDHLLRMWRSKKTPMGFSYDTRCFDSTVTERDIQTEHDIYQCCQLEPAARTAITSLTDRLYCGGPMFNSKGQACGYRRCRASGVLTTSLGNTLTCYLKAQ
6fCON     VDRLLRMWRAKKTPMGFSYDTRCFDSTVTERDIRVEESIYQCCQLDPEARKAITSLNERLYCGGPMFNSRNQSCGYRRCRASGVLTTSLGNTLTCYLKAQ
6gCON     VEYLLKIWRSKKTPMGFSYDTRCFDSTVTERDIRTEESIYQCCELDPVARKAISSLTERLYVGGPMYNSQGQSCGYRRCRASGVLPTSMGNTLTCYLKAM
6hCON     VDRLLKMWRSKKTPMGFSYDTRCFDSTVTERDIRTEQDIYLSCQLDPEARKVIESLTERLYVGGPMYNSKGQLCGQRRCRASGVLPTSMGNTVTCFLKAT
6iCON     VERLLAMWRSKKTPMGFSYDTRCFDSTVTERDICAERDIYLSCQLDPEAQQAIKSLTERLYVGGPMYNSKGQLCGQRRCRASGVLPTSMGNTITCFLKAK
6kCON     VDQLLKMWRSKKTPMGFSYDTRCFDSTVTEHDIKTERDVYLSCKLDPVARKAIESLTERLYIGGPMYNSRGQLCGTRRCRASGVLTTSLGNTMTCFIKAE
6nCON     VDRLLKMWRARKNPMGFSYDTRCFDSTVTERDIRTEHDIYQSCQLEPEARKAITSLTERLYCGGPMYNSRGQLCGIRRCRASGVLPTSLGNTMTCYIKAQ
6oCON     VDYLLKMWRSKKTPMGFSYDTRCFDSTVTERDIHTEHDIYQCCQLDPVARKAITSLTERLYCGGPMTNSRGQSCGYRRCRASGVLTTSLGNTLTCYLKAQ
6rCON     VDYLLSLWRSKKTPMGFSYDTKCFDSTVTERDIRTEESIYQCCQLDPTARKAITSLNERLYCGGPMFNSRGQSCGYRRCRASGVLTTSLGNTLTCYLKAQ
6uCON     VDHLLKLWRSKKTPMGFSYDTRCFDSTVTERDIRTEADIYLSCQLQPEARDAITSLTERLYCGGPMFNSKGQSCGYRRCRASGVLPTSMGNTITCYLKAQ
6vCON     VEYLLRLWRGKKTPMGFSYDTRCFDSTVTERDIKTEESIYQSCQIEPEAKKVIASLTERLYCGGPMFNSRGQNCGYRRCRASGVLPTSMGNTMTCYLKAM
6wCON     VEYLLKVWRSKKTPMGFSYDTRCFDSTVTERDIRTEESIYQCCQLDPQARKAITSLTERLYVGGPMYNSKGQCCGYRRCRASGVLPTSLGNTMTCYIKAM
6w2CON    VEYLLKVWRSKKIPMGFSYDTRCFDSTVTERDIRTEESIYQCCQLDPQARKAITSLTERLYVGGPMYNSKGQCCGYRRCRACGVLPTSLGNTMTCYIKAM
```

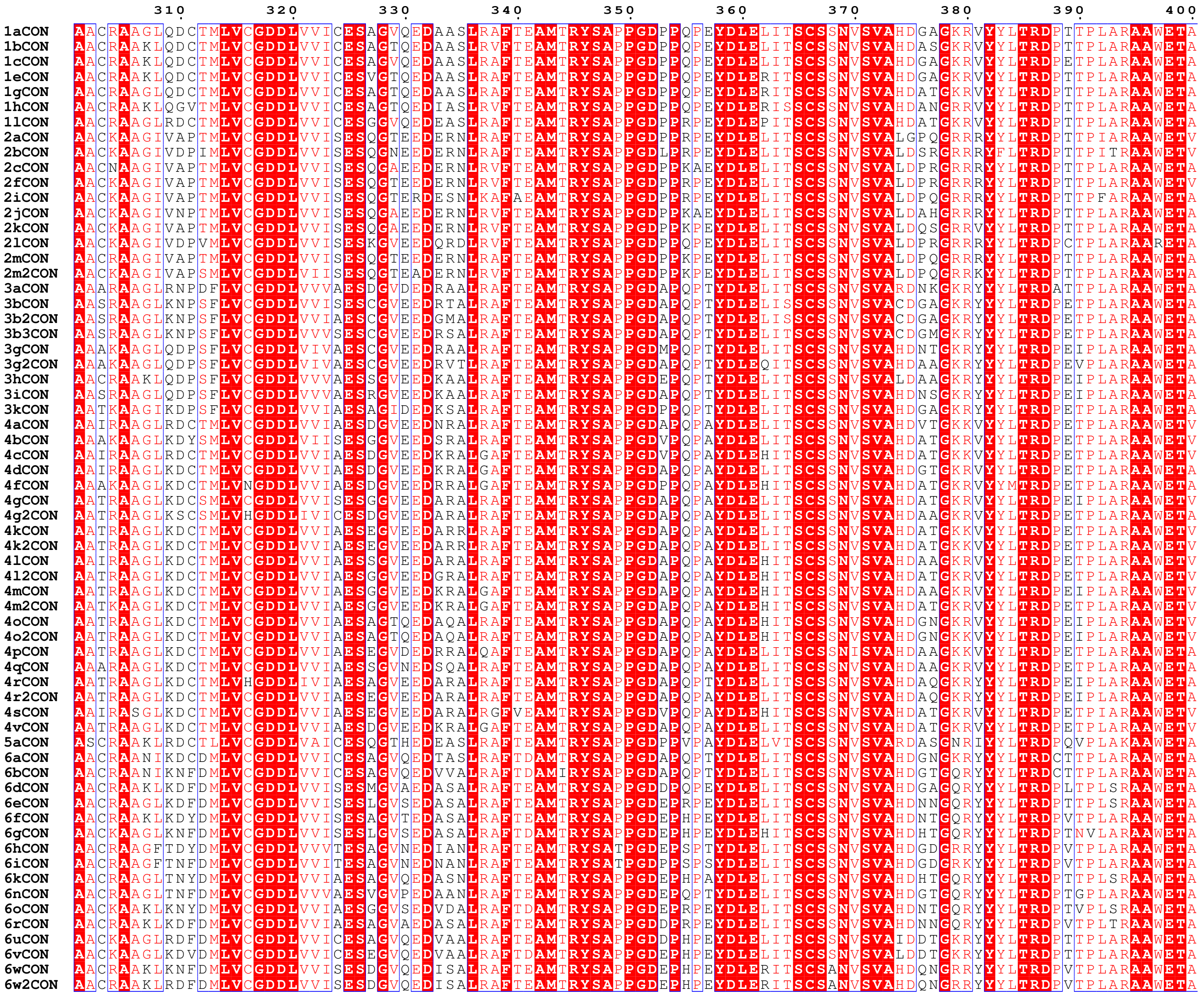

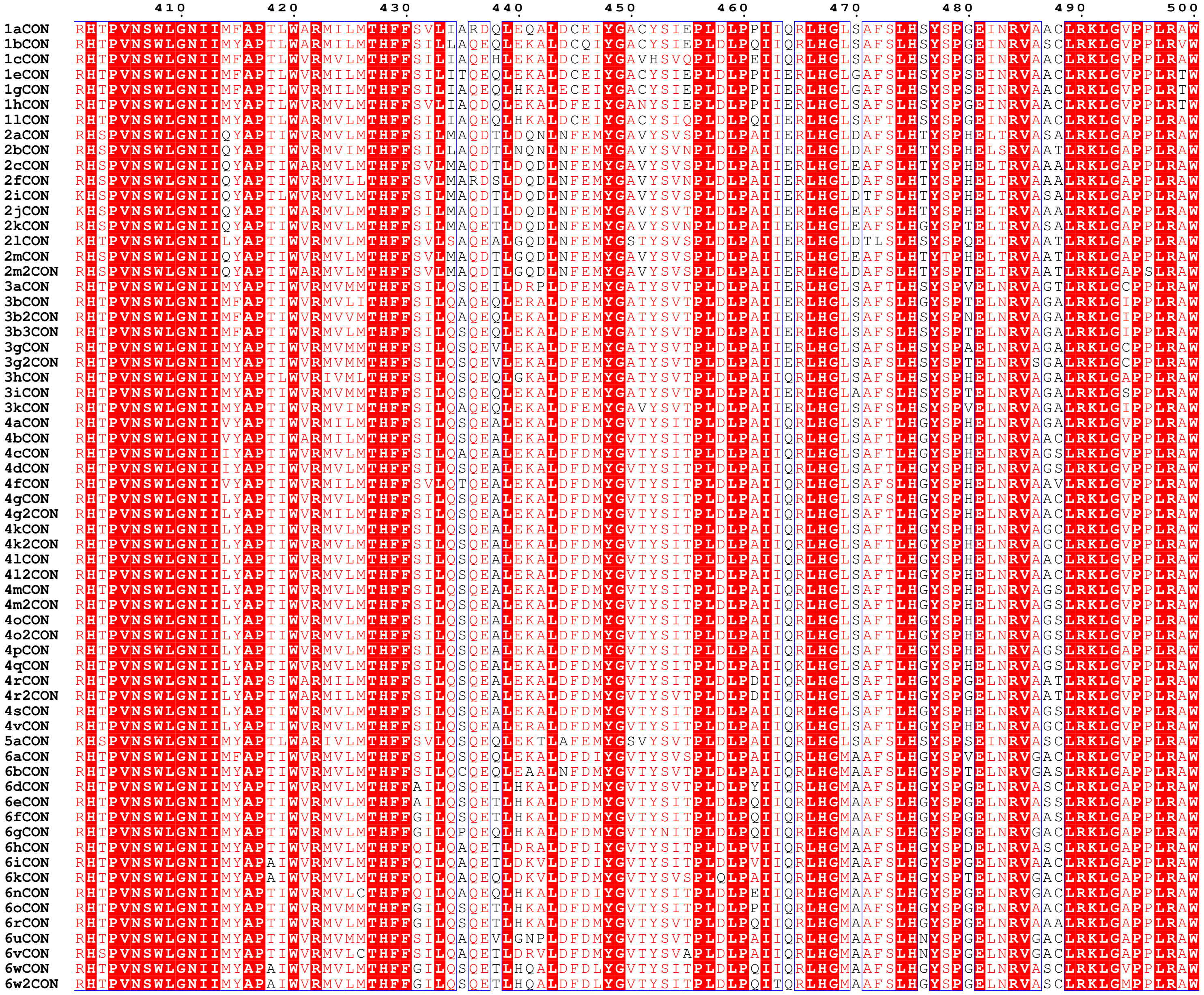
410
420
430
440
450
460
470
480
490
500
1aCON
1bCON
1cCON
1eCON
1gCON
1hCON
1lCON
2aCON
2bCON
2cCON
2fCON
2iCON
2jCON
2kCON
2lCON
2mCON
2m2CON
3aCON
3bCON
3b2CON
3b3CON
3gCON
3g2CON
3hCON
3iCON
3kCON
4aCON
4bCON
4cCON
4dCON
4fCON
4gCON
4g2CON
4kCON
4k2CON
4lCON
4l2CON
4mCON
4m2CON
4oCON
4o2CON
4pCON
4qCON
4rCON
4r2CON
4sCON
4vCON
5aCON
6aCON
6bCON
6dCON
6eCON
6fCON
6gCON
6hCON
6iCON
6kCON
6nCON
6oCON
6rCON
6uCON
6vCON
6wCON
6w2CON

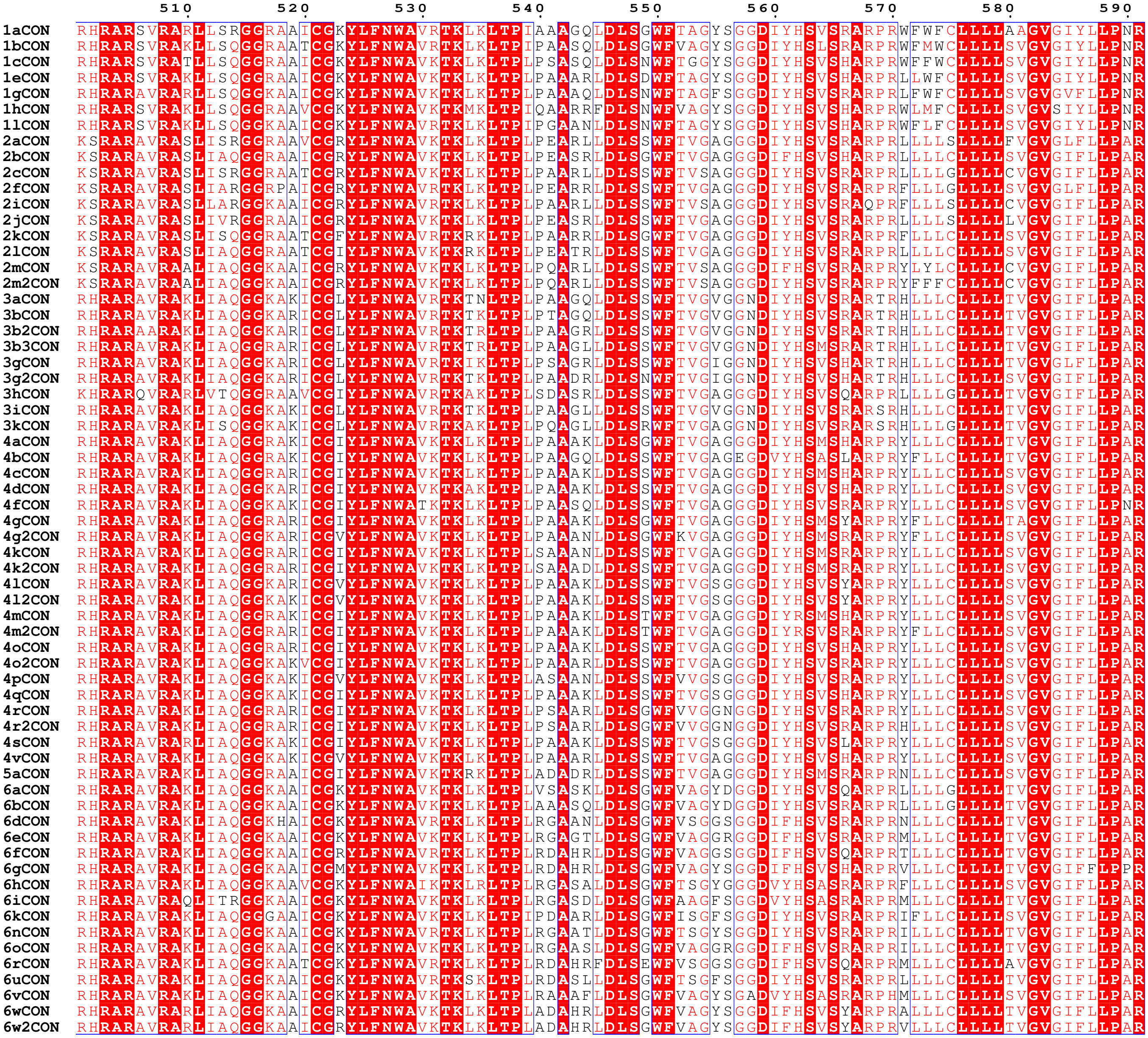

Supplement: Supplementary file 2 [file Table_1.pdf]
